# Soil acidification amendments change the rhizosphere bacterial community of tobacco in a bacterial wilt affected field

**DOI:** 10.1007/s00253-018-9347-0

**Published:** 2018-10-09

**Authors:** Guihua Shen, Shuting Zhang, Xiaojiao Liu, Qipeng Jiang, Wei Ding

**Affiliations:** Laboratory of Natural Products Pesticides, Plant Protection College of Southwest University, Chongqing, 400715 China

**Keywords:** Soil amendments, Oyster shell powder, Bacterial wilt, Soil pH, Microbial community

## Abstract

Application of soil amendments has been wildly used to increase soil pH and control bacterial wilt. However, little is known about causal shifts in the rhizosphere microbial community of crops, especially when the field naturally harbors the disease of bacterial wilt to tobacco for many years due to long-term continuous cropping and soil acidification. In this study, biochar (CP), lime (LM), oyster shell powder (OS) and no soil amendment additions (Control; CK) were assessed for their abilities to improve the soil acidification, change the composition of rhizosphere soil bacterial communities and thus control tobacco bacterial wilt. The results showed that oyster shell powder significantly increased soil pH by 0.77 and reduced the incidence of tobacco bacterial wilt by 36.67% compared to the control. The Illumina sequencing -based community analysis showed that soil amendment applications affected the composition of rhizosphere bacterial community and increased the richness and diversity. In contrast, the richness and diversity correlated negatively to disease incidence. Using LEfSe analyses, 11 taxa were found to be closely related with disease suppression, in which *Saccharibacteria*, *Aeromicrobium*, and *Pseudoxanthomonas* could be potential indicators of disease suppression. Our results suggested that the suppression of bacterial wilt after the application of soil amendments (especially oyster shell powder) was attributed to the improved soil pH and increased bacterial richness and diversity.

**Electronic supplementary material:**

The online version of this article (10.1007/s00253-018-9347-0) contains supplementary material, which is available to authorized users.

## Introduction

Soil amendments are widely used in agriculture to increase soil pH and are considered to have positive effects on soil health and plant growth. Moreover, the use of soil amendments as an alternative for bacterial wilt control has been studied. Earlier studies reported that a soil amendment composed of urea and calcium oxide (CaO) is effective for controlling bacterial wilt in tomato by affecting the pH and nitrite accumulation in the field (Michel et al. 1997; Michel and Mew 1998). Li and Dong (2013) demonstrated that rock dust additions under greenhouse conditions can effectively control tomato bacterial wilt by raising the soil pH and Ca content. A recent study showed that rice straw biochar application could reduce the incidence and severity of tobacco bacterial wilt disease (Zhang et al. 2017). However, little work has been done in acidic tobacco-growing soils where soil amendments are used to control tobacco bacterial wilt.

Tobacco bacterial wilt, which is caused by *Ralstonia solanacearum*, is a widespread and destructive soil-borne disease (Genin 2010). As is typical of soil-borne diseases, the occurrence and prevalence of bacterial wilt are closely related to soil quality. Long-term continuous cropping and excessive use of chemical fertilizers have led to the degradation of soil quality, which is reflected by soil acidification, pollutant accumulation, and biodiversity deterioration. Moreover, soil acidification is closely related to bacterial wilt. The average soil pH in fields infected by bacterial wilt disease was much lower than that in non-disease fields, and the proportion of infected soils with pH lower than 5.5 was much higher than that of non-infected soils in south China (Li et al. 2017). However, soil micro-ecology balance and microbial diversity are necessary to suppress plant soil-borne diseases (Raaijmakers et al. 2009). Therefore, there is a close relationship between soil microorganisms and occurrence of tobacco bacterial wilt.

Soil microbial communities play an important role in plant establishment and growth (Epelde et al. 2010). On the one hand, plants can alter soil microbes through the secretion of root exudates, and on the other hand, soil microbes have the ability to influence the productivity, diversity, and health of plants (Chaparro et al. 2012). Some studies found that the changes in soil microbial community structure were related to the occurrence of soil-borne wilt (Bernard et al. 2012; Niu et al. 2016; Wu et al. 2016).With manipulation of the rhizosphere microbial community, suppression of soil-borne diseases can be enhanced (Mazzola 2007; Qiu et al. 2012; Shen et al. 2015; Yao and Wu 2010). Furthermore, soil pH was the strongest factor that determines microbial community composition, and bacterial relative abundance and diversity is positively affected by soil pH and soil acidification amendments can regulate soil pH (Hartman et al. 2008; Lauber et al. 2009; Zhalnina et al. 2015). However, only a few studies have focused on the relationships between soil microbial community structure and the suppression of bacterial wilt from soil amendment applications.

In the current study, biochar, lime, and oyster shell powder were chosen as soil amendments for an acidic tobacco-growing soil, whereas the treatment without soil amendments was used as a control. We hypothesized that application of different soil amendments could improve the soil pH and change the composition of bacterial communities in the rhizosphere soil, with an increase in some beneficial bacteria, which would lead to a decrease in tobacco bacterial wilt incidence. Therefore, the effects of different soil amendments on the soil pH, incidence of tobacco bacterial wilt, and soil bacterial communities were investigated, and the latter was measured using the Illumina-based sequencing approach. This study aims to provide references for the selection of soil amendment types for acidic tobacco-growing soil and to serve as a theoretical basis for maintaining the sustainability of agricultural systems.

## Materials and methods

### Site description and experimental design

The field experiment was performed from March to July 2015 at a tobacco field in Pengshui Town, Chongqing city of China (38° 39′ N, 104° 04′ E). Tobacco has been cultivated for many years at this site. In previous years, soil acidification and tobacco bacterial wilt outbreaks were serious problems in the experimental field. The variety of tobacco was Yunyan 87, and seedlings were transplanted on May 16, 2015. Fertilizer was applied at a rate of 750 kg hm^−2^ (m(N):m(P _2_O_5_):m(K_2_O) = 8:10:22), according to local tobacco production technology. All fertilizers were applied once, as base manure, before transplanting the tobacco. Randomized block design and triplicated plot were used in the experiment. Each plot with an area of 66.7m^2^ was planted with 110 plants, consisting of five 15 m-long rows, spaced 1.1 m apart. The distance between adjacent plots was 1 m. The four treatments were as follows: (1) CK, control without soil amendment; (2) CP, application of biochar; (3) LM, application of lime; and (4) OS, application of oyster shell powder. The physiochemical characteristics of the biochar were as follows: pH 9.2, organic carbon 372.38 g/kg, total nitrogen 7.03 g/kg, total phosphorus (P_2_O_5_) 2.23 g/kg, and total potassium (K_2_O) 45.03 g/kg. The main component of the lime was CaO with pH 10.4. Oyster shell powder was purchased from Haixinghaizhiyuan Feedstuff Co., Ltd., Bohai New Area, Hebei, China. The physiochemical characteristics of the oyster shell powder were as follows: pH 9.5, 98.9% calcium, 0.5% protein, 0.1% crude fat, 81.0 mg/kg manganese, 2.9 mg/kg zinc and 285.0 mg/kg iron, 214.0 mg/kg potassium, 2040.0 mg/kg magnesium, and 48.0 mg/kg phosphorous. One thousand two hundred kilograms per square hectometer of each soil amendment was applied with the base manure.

### Soil sampling

Rhizosphere soil samples were collected on July 15, 2015 (at the topping stage of tobacco). At this time, tobacco bacterial wilt was at its peak. Five-point sampling method (three plants per point) was used to collect each soil sample from 15 plants per plot. After removing 0–5 cm topsoil, the soil around the root system was gently shaken off. Then, the soil attached to the root surface was evaluated as rhizosphere soil. The soil samples collected in the field were sealed in a new plastic bag and placed in an ice box. The frozen soil samples were taken back to the laboratory and were quickly sifted through a 2-mm sieve to remove debris and stones. Some soil samples were kept in a − 80 °C freezer until the determination of soil bacteria community structure. The other soil samples were air-dried and ground (< 2 mm) to determine soil pH. The pH of the soils was measured using a 1:2.5 (w: v) soil: water ratio. At the same time as the soil sampling, the incidence of disease was recorded based on observations of typical wilt symptoms, including leaves wilting, vascular bundle browning, and roots turn black and rot. Disease incidence (DI) was expressed as the percentage of diseased plants per total number of plants (Yuan et al. 2014).

### DNA extraction, PCR amplification, and sequencing

Soil total DNA was extracted from 0.4 g of soil using an Omega Biotek Soil DNA Kit (Omega Biotek, Norcross, GA, USA), according to the standard protocol. The extracted DNA was checked on 1% (*w*/*v*) agarose gels, and the purity and quality of the DNA were determined using a ThermoFisher SCIENTIFIC, Waltham, MA, USA. Primers 338 forward (5′-ACTCCTACGGGAGGCAGCAG-3′) and 806 reverse (5′-GGACTACHVGGGTWTCTAAT-3′) were used to amplify the V3–V4 region of the 16S rRNA gene (Xu et al. 2016). The PCR reaction procedure was as follows: an initial denaturation at 95 °C for 3 min; followed by 28 cycles of denaturation at 95 °C for 30 s, annealing at 55 °C for 30 s, and elongation at 72 °C for 45 s; and a final extension at 72 °C for 10 min. PCR products were evaluated using a 2% agarose gel electrophoresis for detection. Amplicons were pooled in equidensity ratios, purified using an AxyPrep DNA Gel Recovery Kit (AXYGEN, Waltham, MA, USA), and submitted to the next-generation sequencing laboratory at Majorbio Biopharm Technology Co., Ltd.(Shanghai, China) for Illumina paired-end library preparation, cluster generation, and 250-bp paired-end sequencing. Sequences are available in the NCBI short-reads archive database under Accession Number SRP135724.

### Bioinformatics and statistical analysis

After removing the adaptors and primer sequences, the raw sequences were quality filtered and assembled by each sample based on their unique barcode using QIIME v1.7.0 (Caporaso et al. 2010). Split sequences for each sample were merged using FLASH v1.2.7 (Mago and Salzberg 2011). The sequences retained for each sample were analyzed following the Uparse software v7.0.100 (Edgar et al. 2011). Briefly, sequences with a collective abundance of over 20 reads were retained and singletons were discarded, and then the remaining sequences were assigned to operational taxonomic units (OTUs) with 97% similarity, followed by chimeras, which were filtered using the UCHIME (Edgar et al. 2011). Finally, the RDP database was used to assign the representative OTUs to the microbial taxa (Wang et al. 2007).

We calculated the Chao, ACE, Shannon, and Simpson indices at an OTU distance of 0.03, using the algorithm of Mothur package (www.mothur.org).To compare bacterial community structures across all samples, the similarity among different treatments was assessed with ANOSIM on a Bray-Curtis distance matrix (Schloss et al. 2009). PCoA (Principal Coordinate Analysis) was performed on Bray-Curtis distance matrices, and the coordinates were used to draw 3D graphical outputs. A comparison of overall microbial distribution in all samples was conducted on the relative abundances of phyla and families using OTUs based on taxonomy.

The linear discriminant analysis (LDA) effect size (LEfSe) method was performed to identify bacterial taxa with significantly different abundances between groups. The Kruskal-Wallis (KW) sum-rank test (*α* = 0.05) was used in the LEfSe analysis to detect features with significantly different abundances between the specified categories, and this was followed by an LDA to estimate the effect size of each differentially abundant feature (logarithmic LDA score > 2.0) (Segata et al. 2011). Taxonomic cladograms illustrated the differences between sample classes on the website http://huttenhower.sph.harvard.edu/galaxy. Furthermore, the taxonomic levels were limited from domain to genus to prevent the interference of redundant data.

Analysis of disease incidence, soil pH, richness, and diversity indices, and the taxa (phyla and families) in amended and non-amended soils were compared using Tukey’s HSD multiple range test (*P* < 0.05). Linear regression analysis (Pearson correlation) was used to evaluate the relationships between bacterial communities and tobacco bacterial wilt incidence. All analyses were performed in SPSS v16.0 (SPSS Inc., Chicago, IL, USA).

## Results

### Bacterial wilt and soil pH

**Fig. 1:**
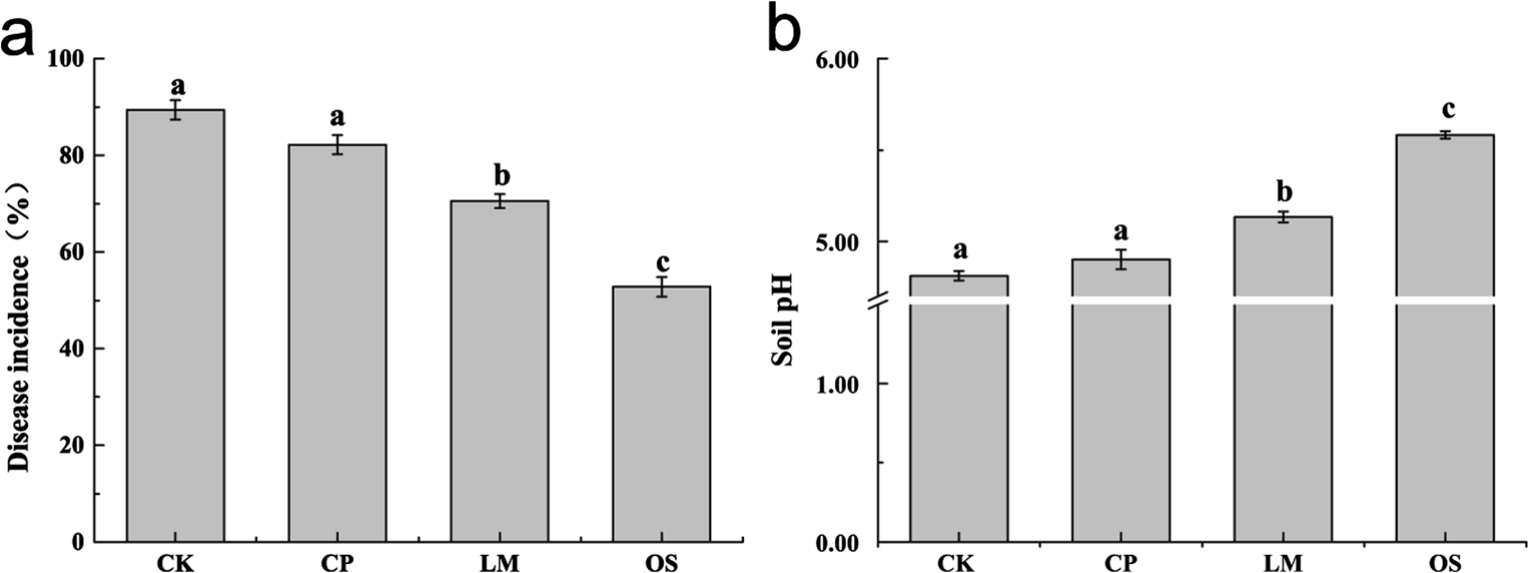
Disease incidence of tobacco bacterial wilt (**a**) and soil pH (**b**) in each treatment. Letters above the bars indicate a significant difference according to Tukey’s multiple comparison test at the *P* < 0.05 level

The disease incidence of tobacco bacterial wilt in the different treatments is shown in Fig. 1a. In general, soil amendments (apart from biochar) reduced the incidence of tobacco bacterial wilt. Compared with the control, the disease incidence in lime and oyster shell powder treatments decreased by 18.89 and 36.67%, respectively, but there were no significant differences between the biochar and control treatments. In addition, soil amendments (apart from biochar) improved the soil pH (Fig. 1b). Oyster shell powder had the highest pH value and increased the soil pH by 0.77 compared to the control; oyster shell powder was followed by lime, which increased the soil pH by 0.32. Linear regression analysis results showed that disease incidence was remarkably negatively correlated with soil pH (*r* = 0.96, *P* < 0.01).

### Bacterial community diversity

In this study, 12 samples were sequenced and compared, and we obtained 674,378 valid sequences, including 295,637,897 base pairs. The average length of each fragment was 438.42 bp. The rarefaction curves were shown in Supplemental Fig. S1. As the curves reached the plateau, it suggested that the sequencing capability was sufficient enough to capture the complete diversity of the bacterial communities. In order to perform downstream analysis at the same sequencing depth, we standardized each sample to 21,595 reads and clustered them into 19,438 OTUs with 97% sequence similarity, with 1454–1854 OTUs per sample. The control treatment had the lowest number of total OTUs (1492 OTUs), and the total number of OTUs in oyster shell powder (1727 OTUs) was the largest. The differences in microbial communities in soil samples were revealed by comparing the richness and diversity indices (Table 1). Richness indices in oyster shell powder treatments were highest and were significantly higher than the control. Soil amendments improved the diversity indices in soil microbial communities, but the difference was not significant.

**Table 1:** Number of observed OTUs, coverage, richness, and diversity (mean ± standard deviation) of soil microbes in each treatment

Variable		CK	CP	LM	OS
OTUs		1492 ± (23)^a^	1624 ± (28)^a^	1636 ± (112)^a^	1727 ± (20)^a^
Coverage		0.977 ± (0.000)^ab^	0.976 ± (0.000)^ab^	0.977 ± (0.001)^a^	0.975 ± (0.001)^b^
Richness	Chao	1909.90 ± (29.80)^a^	1975.40 ± (33.69)^ab^	1989.90 ± (87.04)^ab^	2140.20 ± (3.96)^b^
	ACE	1903.20 ± (23.36)^a^	2005.00 ± (20.82)^ab^	2008.70 ± (76.78)^ab^	2134.20 ± (12.18)^b^
Diversity	Simpson	0.018 ± (0.005)^a^	0.015 ± (0.001)^a^	0.009 ± (0.002)^a^	0.007 ± (0.001)^a^
	Shannon	5.69 ± (0.14)^a^	5.82 ± (0.05)^a^	6.06 ± (0.23)^a^	6.14 ± (0.08)^a^

*OTUs* operational taxonomic units (97% similarity)

Values followed by different superscript letters are significantly different (*P* < 0.05) according to Tukey’s multiple comparison test

**Fig. 2:**
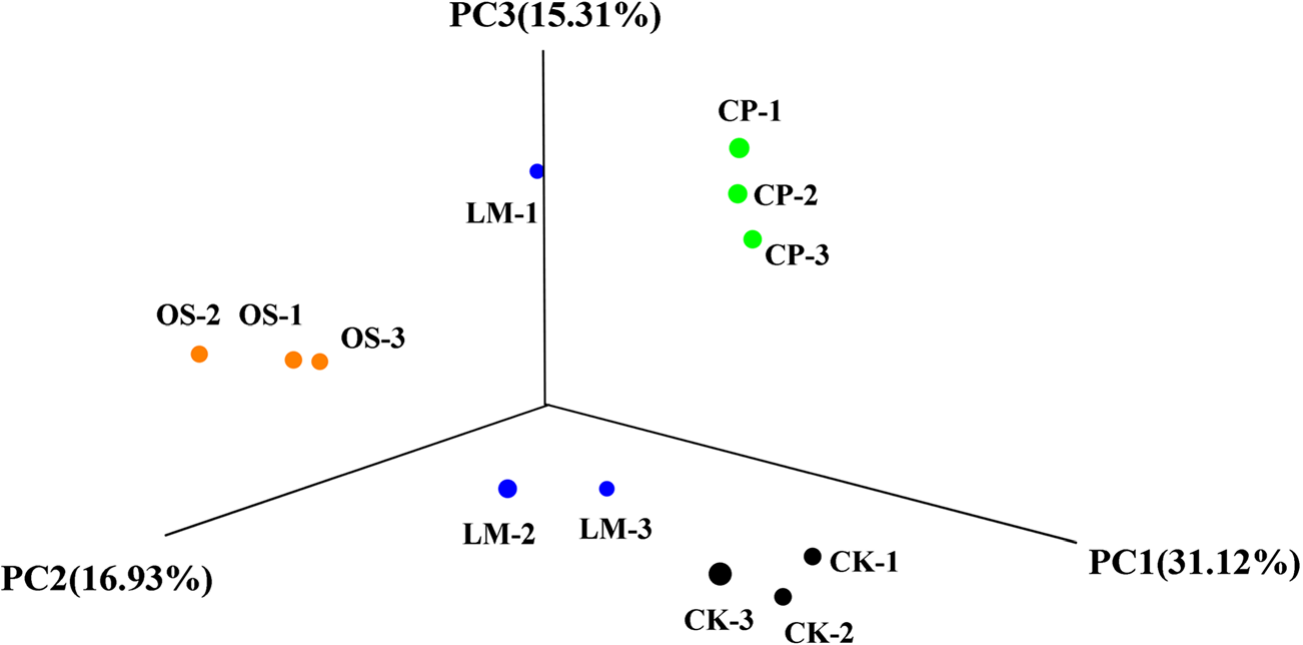
PCoA plot based on Bray-Curtis distances of microbial communities sampled from each treatment

In order to assess the effect of each soil amendment, we used PCoA based on beta-diversity metrics from the Bray-Curtis metric to compare microbial communities in each treatment (Fig. 2). PC1 explained the majority of the variance in the data, representing 30.43% of the variance. The other two principal coordinates explained 16.93% (PC2) and 15.37% (PC3) of the variance. For the PCoA based on the Bray-Curtis distance matrix, control and lime treatments were distributed on the bottom part of the PCoA graph, biochar was located in the upper part, and oyster shell powder was located in the left part. In addition, the microbial community dissimilarity test (Bray-Curtis analysis of similarity, ANOSIM *R* = 0.6111, *P* = 0.001) showed that the differences between groups were greater than those within groups, so the microbial community structure between different groups was distinct.

### Bacterial community composition

**Fig. 3:**
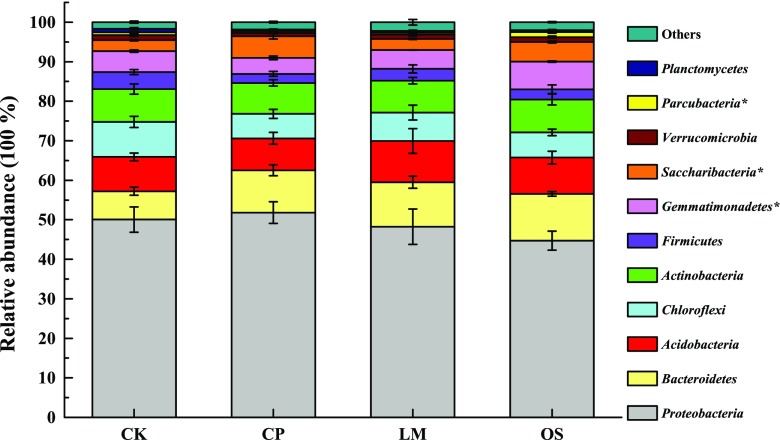
Bacterial community structure in the four treated soils at the phylum level. Other phyla represent < 1% of the total reads. Statistical significance (*P* < 0.05) using Tukey’s multiple comparison test is designated by an asterisk

Based on the Illumina platform analysis, there were 11 phyla in all soils whose average relative abundances were more than 1% (Fig. 3). *Proteobacteria*, *Bacteroidetes*, *Acidobacteria*, *Chloroflexi*, and *Actinobacteria* were the dominant bacteria in all treatments, accounting for 80.44–85.23% of the total OTUs. At the phylum level, the composition of the four treatments was similar, but the relative abundance of each phylum did differ in all samples. *Bacteroidetes* and *Saccharibacteria* were relatively more abundant in amended soil. Specifically, the relative abundance of *Saccharibacteria* showed the following trend: biochar > oyster shell powder > lime > control. In addition, oyster shell powder had the highest relative abundance of *Gemmatimonadetes* and *Parcubacteria*.

**Fig. 4:**
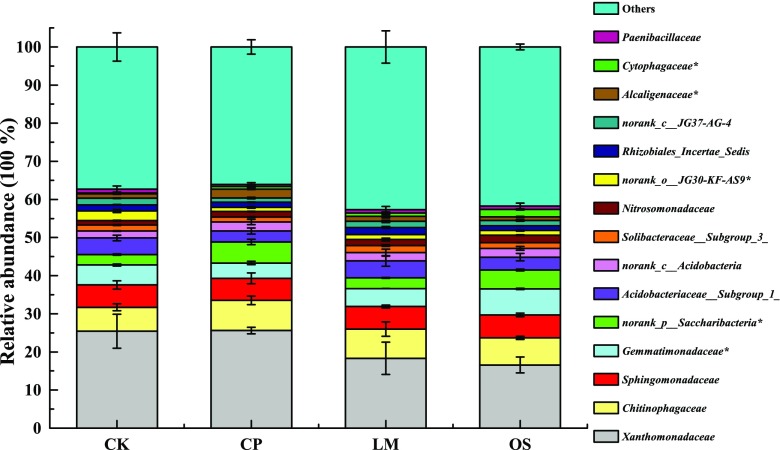
Bacterial community structure in the four treated soils at the family level. Other families represent < 2% of the total reads. Statistical significance (*P* < 0.05) using Tukey’s multiple comparison test is designated by an asterisk

The lower family taxonomic analysis demonstrated that *Xanthomonadaceae* (16.58–25.61%), *Chitinophagaceae* (6.27–7.91%), *Sphingomonadaceae* (5.78–5.96%), and *Gemmatimonadaceae* (4.03–6.85%) were the dominant families in all treatments (Fig. 4). Except for the unidentified family, the relative abundances of *Chitinophagaceae*, *Nitrosomonadaceae*, and *Cytophagaceae* in amended soil were higher than in the control. Furthermore, the relative abundance of *Gemmatimonadaceae* and *Cytophagaceae* in oyster-shell-powder-treated soil increased by 1.62% and 1.74%, respectively, compared to the control. The relative abundance of *Alcaligenaceae* in biochar- and lime-treated soils was higher.

**Fig. 5:**
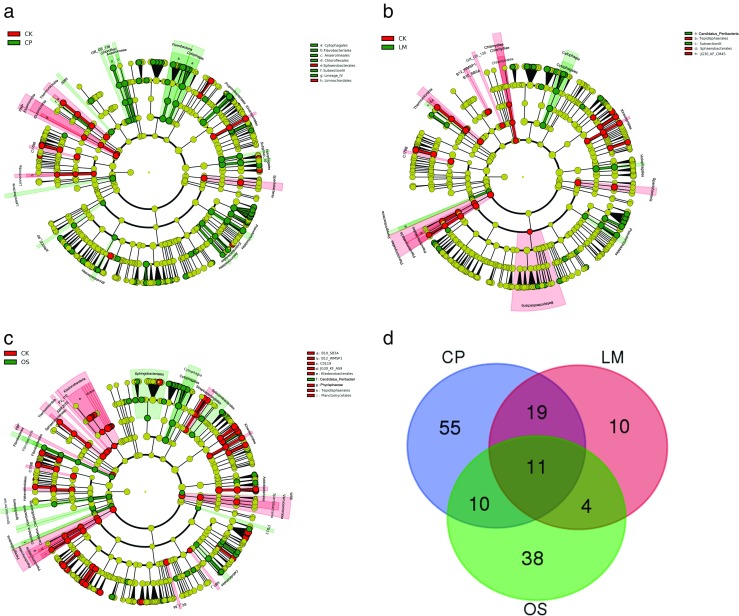
Cladogram indicating the phylogenetic distribution of bacterial lineages under each treatment: **a** biochar (CP), **b** lime LM, **c** oyster shell powder (OS), and control groups (CK); lineages with LDA values higher than 2.0 are displayed. **d** Venn diagram for biochar (94), lime (43), and oyster shell powder groups (62) screened out from LEfSe analyses at the genus level, and the shared 11 taxa are shown in Table 2

We performed LEfSe analyses to examine which taxa differed most between the treatment (biochar, lime, and oyster shell powder) and control groups (Fig. 5a–c). At the genus level, the biochar, lime, and oyster shell powder screened out 94, 43, and 62 major taxa (Supplemental Table S1), respectively. In order to explore the potential taxon indicators for disease suppression from amended and non-amended soils, we take the intersection to find common taxa screened from the biochar, lime, and oyster shell powder groups (Fig. 5d). The results revealed 11 taxa screened from the three groups at the genus level (Table 2). Furthermore, these taxa belong to four phyla (*Bacteroidetes*, *Proteobacteria*, *Actinobacteria*, and *Acidobacteria*) and seven families (*Holophagaceae*, *Nocardioidaceae*, *Cytophagaceae*, *Flavobacteriaceae*, *Xanthomonadaceae*, *Methylophilaceae*, and *Pseudonocardiaceae*).

**Table 2:** Indicators of rhizobacterial communities for disease suppression

Taxon of indicators					LDA score (log10)		
Phylum	Class	Order	Family	Genus	CP	LM	OS
*Bacteroidetes*	*Flavobacteria*	*Flavobacteriales*	*NS9_marine_group*	*–*	4.82	5.07	4.57
*Acidobacteria.*	*Acidobacteria*	*Holophagales*	*Holophagaceae*	*–*	5.84	5.82	5.64
*Actinobacteria*	*Actinobacteria*	*Propionibacteriales*	*Nocardioidaceae*	*Aeromicrobium*	5.21	5.23	5.35
*Bacteroidetes*	*Cytophagia*	*Cytophagales*	*–*	*–*	6.11	5.91	6.23
*Bacteroidetes*	*Cytophagia*	*Cytophagales*	*Cytophagaceae*	*–*	6.06	6.23	5.75
*Bacteroidetes*	*Cytophagia*	*–*	*–*	*–*	6.13	6.25	5.88
*Proteobacteria*	*Deltaproteobacteria*	*Myxococcales*	*BIrii41*	*–*	5.07	4.67	4.70
*Bacteroidetes*	*Flavobacteria*	*Flavobacteriales*	*Flavobacteriaceae*	*Pricia*	5.42	5.98	5.51
*Proteobacteria*	*Gammaproteobacteria*	*Xanthomonadales*	*Xanthomonadaceae*	*Pseudoxanthomonas*	4.81	4.74	4.63
*Proteobacteria*	*Betaproteobacteria*	*Methylophilales*	*Methylophilaceae*	*Methylobacillus*	4.73	5.19	4.69
*Actinobacteria*	*Actinobacteria*	*Pseudonocardiales*	*Pseudonocardiaceae*	*Lechevalieria*	4.66	4.66	4.52

### Correlation of tobacco bacterial wilt incidence with soil bacterial community composition

**Fig. 6:**
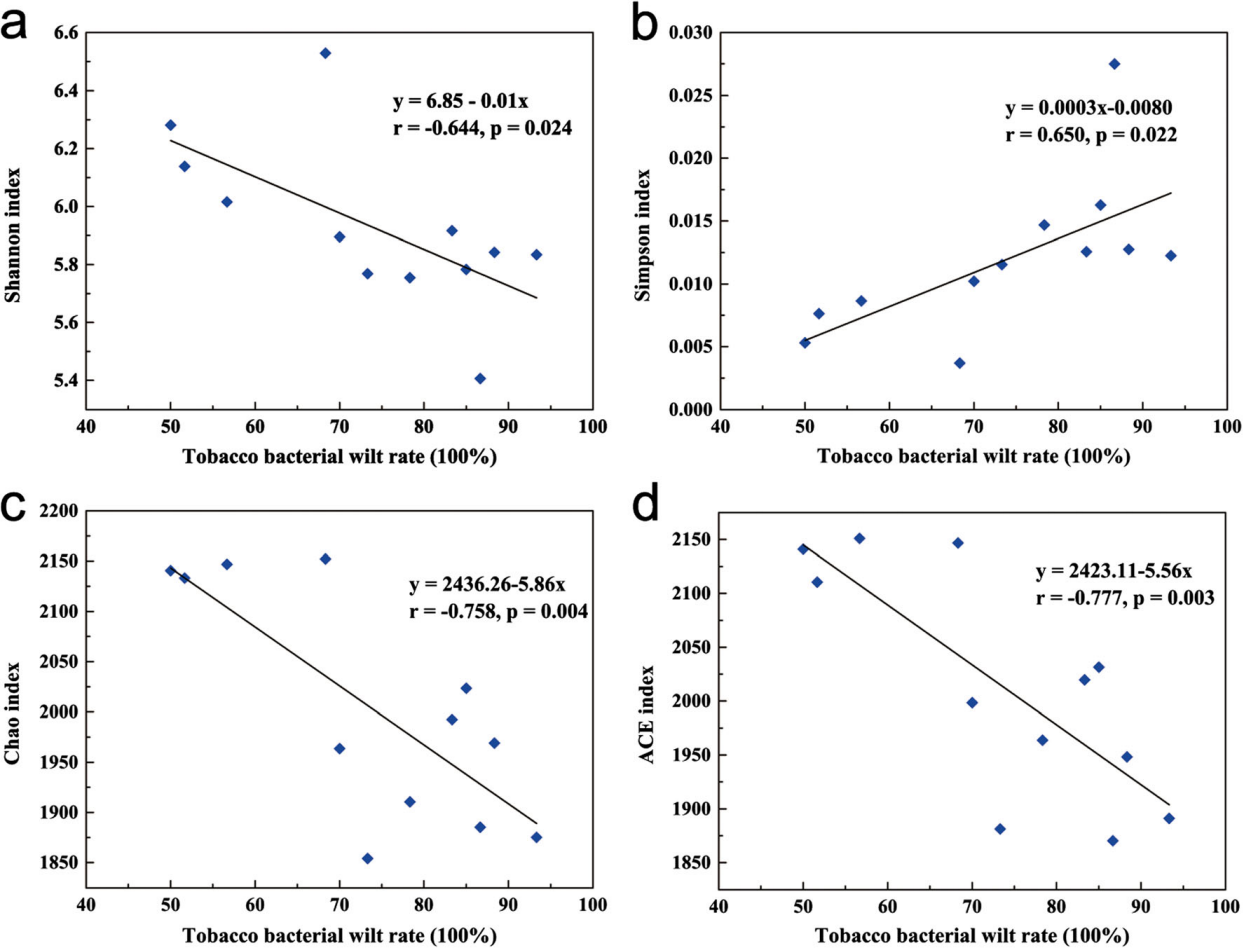
Linear regression analysis of the relationship between tobacco bacterial wilt rate and alpha-diversity. The alpha-diversity: **a** Shannon index, **b** Simpson index, **c** Chao index, and **d** ACE index

Linear regression analysis was used to explore whether the soil bacterial community was associated with tobacco bacterial wilt incidence. The relationship between tobacco disease incidence and alpha-diversity was explored (Fig. 6), and the results showed that the Chao, ACE, and Shannon indices were strongly negatively related to disease incidence, but the Simpson index was positively related to disease incidence. Furthermore, a Pearson analysis was also performed on the relationships between the 11 taxa that were previously screened and the tobacco bacterial wilt incidence. We found that the abundance of five genera was significantly negatively correlated with bacterial wilt incidence (Table 3). There are two identified genera of *Aeromicrobium* and *Pseudoxanthomonas*; two genera belonging to *Holophagaceae* and *Cytophagaceae*; and one genus belonging to *Cytophagales*.

**Table 3:** Correlation between tobacco bacterial wilt rate and abundances of selected microorganisms at the genus level

Taxon of indicators					*r*	*P*
Phylum	Class	Order	Family	Genus		
*Acidobacteria*	*Acidobacteria*	*Holophagales*	*Holophagaceae*	*–*	− 0.667	0.018
*Actinobacteria*	*Actinobacteria*	*Propionibacteriales*	*Nocardioidaceae*	*Aeromicrobium*	− 0.711	0.009
*Bacteroidetes*	*Cytophagia*	*Cytophagales*	*Cytophagaceae*	*–*	− 0.850	0.000
*Proteobacteria*	*Gammaproteobacteria*	*Xanthomonadales*	*Xanthomonadaceae*	*Pseudoxanthomonas*	− 0.614	0.034
*Bacteroidetes*	*Cytophagia*	*Cytophagales*	*–*	*–*	− 0.855	0.000

## Discussion

### Soil amendments improved soil pH and reduced the occurrence of bacterial wilt

High soil pH is especially important for controlling tobacco bacterial wilt (Zhang et al. 2016). In this study, we found that lime and oyster shell powder significantly reduced the incidence of tobacco bacterial wilt by improving soil pH (Fig. 1). This is consistent with a recent study demonstrating that soil pH improvement after lime and wood ash application reduces the occurrence of bacterial wilt (Li et al. 2017). It has been indicated that soil pH directly influences plant disease infection by affecting the survival of soil-borne pathogens and soil microbes (Ghorbani et al. 2008). Growth and reproduction of plant pathogens is inhibited at higher soil pH by affecting iron absorption (Elmer and Pignatello 2011). Acidic soil pH (pH 4.5–5.5) inhibits the growth and antagonistic activity of antagonistic bacteria such as *Pseudomonas fluorescens* and *Bacillus cereus* (Li et al. 2017). In addition, soil acidification affects plant growth and nutrient availability (Rout et al. 2001; Wang et al. 2000). For example, the soluble Al content has a toxic effect on plant growth at lower pH values (Bian et al. 2013; Ryan et al. 2010), and most essential nutrients cannot be directly absorbed by plants in acidic soil (Läuchli and Grattan 2017). Taken together, increasing soil pH with soil amendments can be proposed as a strategy for disease control.

### Soil amendments increased the bacterial community diversity and reduced the occurrence of bacterial wilt

In the present study, we observed that the bacterial community diversity shifted in the amended soils (Table 1). Previous studies have shown that lime and biochar application have a significant impact on bacterial diversity and communities (Tender et al. 2016; Xue et al. 2010). In this study, lime and biochar slightly increased the diversity of soil microbial communities compared to the control. Furthermore, oyster shell powder significantly increased the richness index of soil microbial communities compared to the control. The reason may be that oyster shells contain glycosaminoglycan and aspartic proteinases, which can stimulate growth in soil microbes (Yong et al. 2016). Our tests for a correlation between diversity and bacterial wilt rate indicated that the incidence of tobacco bacterial wilt increased with a reduction in soil microbial diversity (Fig. 6). This is in line with previous findings that found that long-term use of chemical fertilizer leads to a decrease in microbial diversity in the soil and an increase in peanut wilt (Liu et al. 2015). It has been reported that soil microbial diversity confers protection against soil-borne disease and hinders the establishment of soil pathogens (Alabouvette et al. 2004; Brussaard et al. 2007; Dey et al. 2012). Overall, the increase in the soil bacterial richness and diversity after soil amendment application may contribute to the suppression of bacterial wilt in acidic tobacco-growing areas.

### Soil amendments changed the composition of bacterial communities

Broad and complex shifts in the microbial community composition can contribute to soil disease suppression (Kinkel et al. 2011), but it may also be related to the presence or alteration of specific microbial populations (Bonilla et al. 2012). Our results showed that soil amendments increased the relative abundance of *Bacteroidetes* and *Saccharibacteria*. *Bacteroidetes* are copiotrophic soil bacteria, suitable for using labile substrates and surviving in rhizosphere conditions (Goldfarb et al. 2011). It is suggested that this increased relative abundance may derive from soil amendments improving the soil environment, such as increasing soil pH. The phylum candidatus *Saccharibacteria* was formerly known as *Candidate Division TM7* (Kindaichi et al. 2016). Due to the isolation and characterization of very few strains of *Saccharibacteria*, information on their potential disease control is limited. Notably, recent studies revealed that *Saccharibacteria* was the most prominent biomarker in bacterial wilt disease suppression (Zhang et al. 2017). We observed that the relative abundance of *Saccharibacteria* was higher in amended soil with a corresponding decrease in tobacco bacterial wilt in the current study, suggesting that *Saccharibacteria* may play a potential role in evaluating the disease suppression effects of soil amendment applications.

### Key taxa characterized the bacterial wilt suppression

Using LEfSe and Venn diagram analyses, 11 taxa were found to be indicative of tobacco health (Fig. 5, Table 2). Additionally, correlation analysis showed that the abundance of 5 genera was significantly negatively correlated with the bacterial wilt rate (Table 3). *Aeromicrobium* is a member of the actinobacteria family. As a group of bacteria with high concentrations in soils, actinobacteria play an important role in plant disease suppression and growth promotion (Palaniyandi et al. 2013). Meanwhile, antibiotics produced by actinobacteria have been proven to suppress various plant diseases (Agbessi et al. 2003; Kim et al. 2007; Lee et al. 2012). Miller et al. (1991) first described the genus *Aeromicrobium* with a single species, *Aeromicrobium erythreum*, that produced the macrolide antibiotic erythromycin. Based on these results, it has been speculated that *Aeromicrobium* plays an important role in disease suppression. Earlier studies demonstrated that *Xanthomonadaceae* may play an important role in the control of tobacco bacterial wilt caused by *Ralstonia solanacearum* (Wu et al. 2014). Moreover, *Pseudoxanthomonas* are Gram-negative bacteria belonging to the *Xanthomonadaceae* family*,* which is beneficial for the suppression of soil-borne diseases and provides a healthy soil environment for the growth of the root system (Wang et al. 2017). The other three genera that are significantly related to the incidence of tobacco bacterial wilt have not been identified as of yet; therefore, their role in disease suppression is unclear.

Altogether, soil pH and rhizosphere bacterial communities which are important for suppression of bacterial wilt disease were affected by soil acidification amendments to varying degrees. The suppressive effect of these soil amendments on tobacco bacterial wilt varied as influenced by the difference of physico-chemical properties of the soil amendments. Notably, oyster shell powder can significantly suppress the occurrence of bacterial wilt attributed to higher soil pH, bacterial community diversity, and beneficial bacteria. The higher level of bacterial wilt suppression observed in oyster shell powder was associated with its source and composition. The main component of oyster shell powder derived from crustaceans is chitin, and the breakdown of chitin present in the oyster shell powder releases ammonia, which probably accounts for the remarkable rise in pH (Bai et al. 2016; Hampson and Coombes 1995). Moreover, oyster shell powder can be used as a carbon source of soil microorganisms for microbial metabolism, thereby increasing soil microbial diversity (Cohen-Kupiec and Chet 1998). Soil amended with chitin can increase some beneficial bacteria that are likely to play an important role as pathogen antagonists (Mitchell and Alexander 1961; Veliz et al. 2017). Similarly, we found that *Saccharibacteria*, *Aeromicrobium*, and *Pseudoxanthomonas* were potential indicators of disease suppression after application of soil amendments in this study.

This paper concentrated only on the short-term responses of soil pH and soil microbial communities after application of soil amendments at the topping stage of tobacco. However, whether soil amendments had a persistent effect on soil pH and soil microbial communities or what the likely duration of that effect would be was not investigated. Although this is a limitation, this study can clearly elucidate the increase in soil pH and shifts in the soil bacterial communities induced by different soil amendments and provide information on proper application of disease control in an acidic tobacco-growing soil.

Our results demonstrated that oyster shell powder application was more effective at reducing the incidence of tobacco bacterial wilt and improving the soil pH than lime and biochar. Besides, soil amendments increased the diversity and species richness of the bacterial community. The abundances of *Saccharibacteria*, *Aeromicrobium*, and *Pseudoxanthomonas* were potential indicators of increased disease suppression in amended soil. Furthermore, the temporal changes in soils that occur after soil amendment additions and the long-term influences of soil amendments on soil status should be considered when selecting the types of soil amendments to be used. Taken together, we recommend soil amendments (especially oyster shell powder) in order to improve soil pH and increase the bacterial richness and diversity of acidic tobacco-growing soils and thus contribute to the suppression of tobacco bacterial wilt.

## Electronic supplementary material

ESM 1 (PDF 953 kb)
