# Supplementary material for: Soil acidification amendments change the rhizosphere bacterial community of tobacco in a bacterial wilt affected field
Source: Appl Microbiol Biotechnol. 2018 Oct 9;102(22):9781–91. doi: 10.1007/s00253-018-9347-0 (PMC6208964; doi:10.1007/s00253-018-9347-0)
Supplement: Supplementary file 1 — (PDF 953 kb) [file 253_2018_9347_MOESM1_ESM.pdf]

APPLIED MICROBIOLOGY AND BIOTECHNOLOGY

Soil acidification amendments change the rhizosphere bacterial community of tobacco in a bacterial wilt affected field

Guihua SHEN, Shuting ZHANG, Xiaojiao LIU, Qipeng JIANG, and Wei Ding*
Laboratory of Natural Products Pesticides, Plant Protection College of Southwest University, Chongqing, 400715, China
Email：dwing818@163.com
Telephone number: 023-68250218

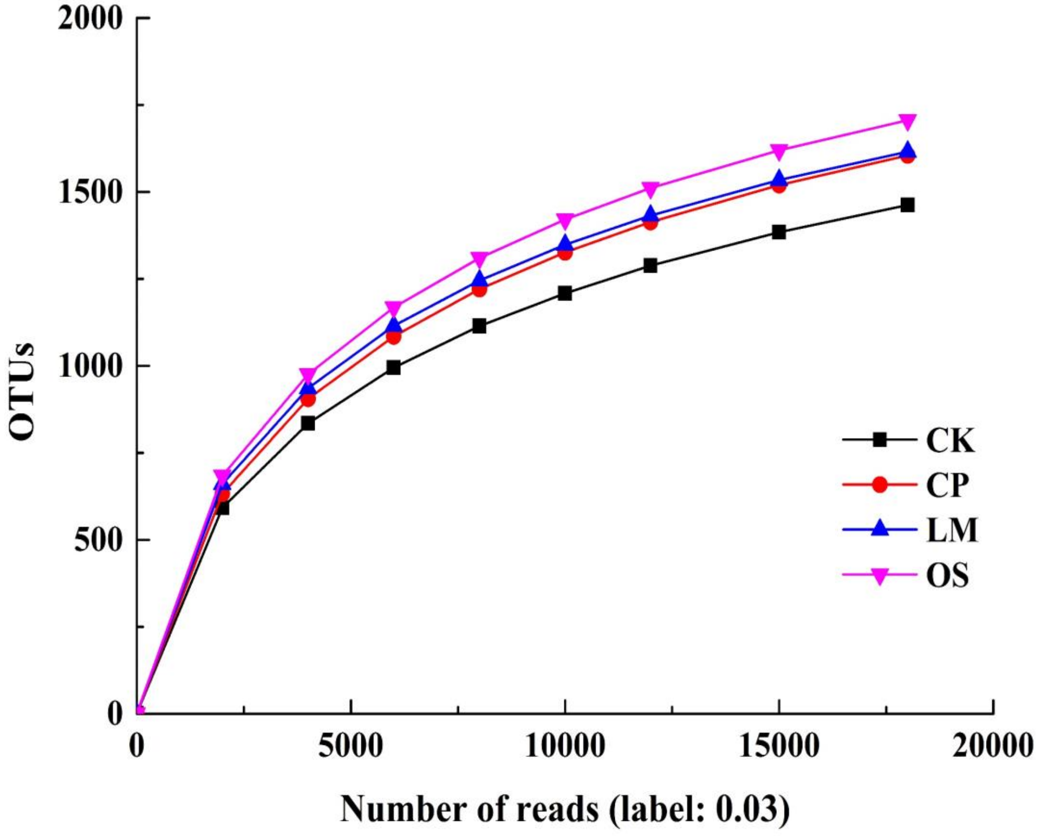

Fig S1 Rarefaction curves of 16S rRNA genes of 97% similarity from rhizosphere soils amended with biochar (CP), lime (LM), oyster shell powder (OS) and soils with no amendment (CK)

Table S1. The screen out genera between the treatment (biochar, lime and oyster shell powder) and control groups

Taxonomy	Mean	Group	LDA	*P* value
Bacteria, Proteobacteria, Betaproteobacteria, Burkholderiales, Oxalobacteraceae, Pseudoduganella	5.29	CP	5.02	0.04
Bacteria, Actinobacteria, Actinobacteria, Streptosporangiales, Streptosporangiaceae, Streptosporangium	3.58			-
Bacteria, Firmicutes, Bacilli, Lactobacillales, Streptococcaceae	6.06			-
Bacteria, Proteobacteria, Deltaproteobacteria, Myxococcales, Archangiaceae	4.44			-
Bacteria, Proteobacteria, Alphaproteobacteria, Rhizobiales, Brucellaceae, Ochrobactrum	4.85			-
Bacteria, Actinobacteria, Actinobacteria, Solirubrobacterales, YNPFFP1	5.55			-
Bacteria, Proteobacteria, Gammaproteobacteria, Xanthomonadales, Xanthomonadaceae	6.65	CP	6.29	0.05
Bacteria, Proteobacteria, Alphaproteobacteria, Sphingomonadales, Erythrobacteraceae, Altererythrobacter	5.64			-
Bacteria, Proteobacteria, Gammaproteobacteria, Oceanospirillales, Hahellaceae, Hahella	3.82			-
Bacteria, Firmicutes, Clostridia, Clostridiales, Peptostreptococcaceae, Paeniclostridium	5.43			-
Bacteria, Actinobacteria, Actinobacteria, Kineosporiales	5.69	CK	5.18	0.05
Bacteria, Proteobacteria, Betaproteobacteria, Burkholderiales	7.32			-
Bacteria, Bacteroidetes, Sphingobacteriia, Sphingobacteriales, Chitinophagaceae, Lacibacter	4.94	CP	4.71	0.04
Bacteria, Actinobacteria, Actinobacteria, Acidimicrobiales, Acidimicrobiaceae	5.14			-
Bacteria, Proteobacteria, Gammaproteobacteria, Xanthomonadales, Xanthomonadaceae, Dokdonella	5.67			-

Bacteria, Proteobacteria, Alphaproteobacteria, Sphingomonadales, Sphingomonadaceae, Zymomonas	0.00			-
Bacteria, Chlorobi, Chlorobia, Chlorobiales, OPB56	5.77			-
Bacteria, Proteobacteria, Alphaproteobacteria, Rhizobiales, Phyllobacteriaceae	6.57			-
Bacteria, Bacteroidetes, Sphingobacteriia, Sphingobacteriales, Sphingobacteriaceae, Nubsella	0.00			-
Bacteria, Actinobacteria, Actinobacteria, Pseudonocardiales, Pseudonocardiaceae, Kutzneria	4.55			-
Bacteria, Elusimicrobia, Elusimicrobia, Lineage_IIc	0.00			-
Bacteria, Elusimicrobia, Elusimicrobia, Lineage_IIb	4.73			-
Bacteria, Elusimicrobia, Elusimicrobia, Lineage_IIa	3.88			-
Bacteria, Proteobacteria, Alphaproteobacteria, Rhizobiales, Hyphomicrobiaceae, Rhodoplanes	5.89			-
Bacteria, Bacteroidetes	4.88			-
Bacteria, Actinobacteria, Actinobacteria, Corynebacteriales, Nocardiaceae, Rhodococcus	5.47			-
Bacteria, Gemmatimonadetes, Gemmatimonadetes, Longimicrobiales	5.07			-
Bacteria, Proteobacteria, Alphaproteobacteria, Rhizobiales, Xanthobacteraceae, Pseudolabrys	6.55			-
Bacteria, Verrucomicrobia, OPB35_soil_group, Pedosphaera	4.41			-
Bacteria, Proteobacteria, Gammaproteobacteria, Pseudomonadales, Moraxellaceae	4.88	CP	4.62	0.04
Bacteria, Acidobacteria, Acidobacteria, Subgroup_10, ABS_19	5.71	CP	5.22	0.05
Bacteria, Bacteroidetes, Flavobacteriia, Flavobacteriales, Flavobacteriaceae	6.41			-

Bacteria, Ignavibacteriae, Ignavibacteria, Ignavibacteriales	0.00			-
Bacteria, Actinobacteria, Actinobacteria, Propionibacteriales	6.64	CP	6.14	0.05
Bacteria, Proteobacteria, Deltaproteobacteria, Bdellovibrionales, Bdellovibrionaceae, OM27_clade	4.16			-
Bacteria, Bacteroidetes, Cytophagia, Cytophagales, Cytophagaceae, Dyadobacter	4.98	CP	4.72	0.05
Bacteria, Proteobacteria, Alphaproteobacteria, Sphingomonadales, Sphingomonadaceae, Sphingopyxis	5.83			-
Bacteria, Deinococcus_Thermus, Deinococci	5.43			-
Bacteria, Proteobacteria, Gammaproteobacteria, Legionellales, Legionellaceae	5.00			-
Bacteria, Proteobacteria, Gammaproteobacteria, Thiotrichales, EV818SWSAP88	3.93			-
Bacteria, Bacteroidetes, Sphingobacteriia, Sphingobacteriales, Sphingobacteriaceae, Sphingobacterium	5.42			-
Bacteria, Verrucomicrobia, Opitutae	5.85			-
Bacteria, Proteobacteria, Deltaproteobacteria, Bdellovibrionales	5.84			-
Bacteria, Chloroflexi, Ktedonobacteria, Ktedonobacterales, Ktedonobacteraceae	5.21			-
Bacteria, Bacteroidetes, Bacteroidia, Bacteroidales, Porphyromonadaceae, Petrimonas	5.09			-
Bacteria, Firmicutes, Bacilli, Lactobacillales, Carnobacteriaceae	4.44			-
Bacteria, Proteobacteria, Gammaproteobacteria, Enterobacteriales	5.46	CP	5.07	0.05
Bacteria, Candidatus_Berkelbacteria	4.26			-
Bacteria, Bacteroidetes, Sphingobacteriia, Sphingobacteriales, Chitinophagaceae, Dinghuibacter	3.97			-

Bacteria, Proteobacteria, Alphaproteobacteria, Rhizobiales, Rhizobiales_Incertae_Sedis	6.82			-
Bacteria, Proteobacteria, Gammaproteobacteria, Pseudomonadales, Moraxellaceae, Acinetobacter	4.79			-
Bacteria, Proteobacteria, Deltaproteobacteria, Myxococcales, P3OB_42	5.37			-
Bacteria, Proteobacteria, Alphaproteobacteria, Rhizobiales, MNG7	4.61			-
Bacteria, Actinobacteria, Actinobacteria, Gaiellales, Gaiellaceae	6.00			-
Bacteria, Proteobacteria, Alphaproteobacteria, Rhizobiales, Hyphomicrobiaceae	6.74			-
Bacteria, Actinobacteria, Actinobacteria, Catenulisporales, Catenulisporaceae, Catenulispora	5.45			-
Bacteria, Firmicutes, Erysipelotrichia	5.32			-
Bacteria, Proteobacteria, Alphaproteobacteria, Rhizobiales	5.76			-
Bacteria, Verrucomicrobia, OPB35_soil_group	6.18			-
Bacteria, Planctomycetes, Phycisphaerae	6.11			-
Bacteria, Firmicutes, Bacilli, Bacillales, Family_XII_o__Bacillales, Exiguobacterium	4.76			-
Bacteria, Proteobacteria, Alphaproteobacteria, Rhodospirillales, Acetobacteraceae	6.19	CK	5.55	0.05
Bacteria, Bacteroidetes, Flavobacteriia, Flavobacteriales, Flavobacteriaceae, Arenibacter	4.53	CP	4.59	0.04
Bacteria, Armatimonadetes, Chthonomonadetes, Chthonomonadales, Chthonomonadaceae	4.28			-
Bacteria, Proteobacteria, Betaproteobacteria, Burkholderiales, Comamonadaceae, Aquincola	5.37			-
Bacteria, Firmicutes, Bacilli, Lactobacillales	6.34			-

Bacteria, Actinobacteria, Actinobacteria, Micrococcales, Bogoriellaceae	4.39			-
Bacteria, Proteobacteria, Betaproteobacteria, Burkholderiales, Oxalobacteraceae	6.06			-
Bacteria, Actinobacteria, Actinobacteria, Catenulisporales, Catenulisporaceae	5.45			-
Bacteria, Verrucomicrobia, Spartobacteria	6.39	CK	5.66	0.05
Bacteria, Chlorobi, Chlorobia	5.77			-
Bacteria, Proteobacteria, Gammaproteobacteria, Thiotrichales	3.93			-
Bacteria, Verrucomicrobia, Spartobacteria, Chthoniobacterales	3.93			-
Bacteria, Proteobacteria, Gammaproteobacteria, Xanthomonadales, Nevskiaceae, Alkanibacter	6.21			-
Bacteria, Deinococcus_Thermus, Deinococci, Deinococcales, Deinococcaceae, Deinococcus	3.93			-
Bacteria, Proteobacteria, Deltaproteobacteria, Myxococcales, Sandaracinaceae	4.70			-
Bacteria, Bacteroidetes, Sphingobacteriia, Sphingobacteriales, Chitinophagaceae, Segetibacter	4.46			-
Bacteria, Proteobacteria, Betaproteobacteria, Burkholderiales, Burkholderiaceae	0.00			-
Bacteria, Actinobacteria, Actinobacteria, Gaiellales	6.78			-
Bacteria, Proteobacteria, Gammaproteobacteria, Cellvibrionales, Cellvibrionaceae, Cellvibrio	5.03			-
Bacteria, Bacteroidetes, Flavobacteriia, Flavobacteriales, Flavobacteriaceae, Muricauda	0.00			-
Bacteria, Acidobacteria, Acidobacteria, Solibacterales	6.83			-
Bacteria, Bacteroidetes, Sphingobacteriia, Sphingobacteriales, AKYH767	4.77			-

Bacteria, Acidobacteria, Acidobacteria	7.00			-
Bacteria, Firmicutes, Erysipelotrichia, Erysipelotrichales, Erysipelotrichaceae, Turicibacter	5.32			-
Bacteria, Proteobacteria, Gammaproteobacteria, Cellvibrionales, Porticoccaceae	4.26			-
Bacteria, Proteobacteria, Alphaproteobacteria, Rickettsiales, Rickettsiaceae, Candidatus_Trichorickettsia	0.00			-
Bacteria, Proteobacteria, Alphaproteobacteria, Rhodobacterales, Rhodobacteraceae, Rubellimicrobium	4.60	CP	4.54	0.05
Bacteria, Proteobacteria, Alphaproteobacteria, Rhizobiales, Rhizobiales_Incertae_Sedis, Rhizomicrobium	6.78			-
Bacteria, Deinococcus_Thermus	5.43			-
Bacteria, Proteobacteria, Alphaproteobacteria, Rhizobiales, Methylobacteriaceae, Methylobacterium	4.44			-
Bacteria, Proteobacteria, Alphaproteobacteria, Rhizobiales, Hyphomicrobiaceae, Devosia	6.59			-
Bacteria, Firmicutes, Erysipelotrichia, Erysipelotrichales, Erysipelotrichaceae	5.32			-
Bacteria, Proteobacteria, Alphaproteobacteria, Rhodobacterales, Rhodobacteraceae, Paracoccus	5.01	CP	4.55	0.05
Bacteria, Chloroflexi, Ktedonobacteria, Ktedonobacterales, Thermosporotrichaceae, Thermosporothrix	4.72			-
Bacteria, Proteobacteria, Gammaproteobacteria, Xanthomonadales, Xanthomonadales_Incertae_Sedis, Steroidobacter	5.87	CP	5.43	0.05
Bacteria, Proteobacteria, Alphaproteobacteria, Rickettsiales, Mitochondria	4.20			-
Bacteria, Proteobacteria, Alphaproteobacteria, Sphingomonadales, Sphingomonadaceae, Sphingomonas	7.41			-
Bacteria, Actinobacteria, Actinobacteria, Streptomycetales, Streptomycetaceae, Streptomyces	6.12			-
Bacteria, Proteobacteria, Gammaproteobacteria, Pseudomonadales, Pseudomonadaceae	6.22	CP	5.85	0.05

Bacteria, Chloroflexi, Ktedonobacteria, Ktedonobacterales, 1959_1	4.72			-
Bacteria, Planctomycetes, Phycisphaerae, Tepidisphaerales	6.10			-
Bacteria, Proteobacteria, Alphaproteobacteria, Alphaproteobacteria_Incertae_Sedis, Micavibrio	4.92	CP	4.73	0.04
Bacteria, Proteobacteria, Gammaproteobacteria, Xanthomonadales, Xanthomonadaceae, Panacagrimonas	4.85	CP	4.58	0.05
Bacteria, Proteobacteria, Deltaproteobacteria, Myxococcales, mle1_27	3.93			-
Bacteria, Actinobacteria, Actinobacteria, Frankiales, Geodermatophilaceae	5.65			-
Bacteria, Acidobacteria, Acidobacteria, Solibacterales, Solibacteraceae__Subgroup_3_, AKIW659	4.51	CK	4.79	0.04
Bacteria, Chloroflexi, Thermomicrobia, Sphaerobacterales	5.86	CK	5.21	0.05
Bacteria, Proteobacteria, Gammaproteobacteria, Xanthomonadales, Xanthomonadaceae, Lysobacter	6.30	CP	5.80	0.05
Bacteria, Proteobacteria, Betaproteobacteria, Neisseriales, Neisseriaceae	4.25			-
Bacteria, Proteobacteria, Gammaproteobacteria, Methylococcales	3.58			-
Bacteria, Proteobacteria, Alphaproteobacteria, Sphingomonadales, Erythrobacteraceae	6.11			-
Bacteria, Peregrinibacteria, Candidatus_Peribacteria	3.97			-
Bacteria, Proteobacteria, Gammaproteobacteria, Xanthomonadales, Xanthomonadaceae, Luteimonas	6.06			-
Bacteria, Proteobacteria, Alphaproteobacteria, Rhizobiales, Rhodobiaceae, Parvibaculum	5.71			-
Bacteria, Actinobacteria, Actinobacteria, Micrococcales, Demequinaceae, Lysinimicrobium	3.97			-
Bacteria, Bacteroidetes, Sphingobacteriia, Sphingobacteriales, Chitinophagaceae, Crenotalea	6.43			-

Bacteria, Proteobacteria, Gammaproteobacteria, Xanthomonadales, Xanthomonadaceae, Arenimonas	6.02			-
Bacteria, Firmicutes, Clostridia, Clostridiales, Clostridiaceae_1	4.54			-
Bacteria, Chloroflexi, P2_11E	4.67			-
Bacteria, Planctomycetes, Phycisphaerae, Phycisphaerales	4.93			-
Bacteria, Firmicutes, Bacilli, Bacillales, Bacillaceae, Oceanobacillus	4.64			-
Bacteria, Bacteroidetes, Sphingobacteriia, Sphingobacteriales, Sphingobacteriaceae, Mucilaginibacter	6.33			-
Bacteria, Bacteroidetes, Flavobacteriia, Flavobacteriales, Flavobacteriaceae, Confluentibacter	5.05			-
Bacteria, Bacteroidetes, Flavobacteriia, Flavobacteriales, Cryomorphaceae, Fluviicola	5.77	CP	5.45	0.05
Bacteria, Proteobacteria, Betaproteobacteria, Burkholderiales, Comamonadaceae, Delftia	3.68			-
Bacteria, Firmicutes, Bacilli, Lactobacillales, Streptococcaceae, Streptococcus	4.66	CK	4.57	0.05
Bacteria, Chlamydiae, Chlamydiae, Chlamydiales	5.05			-
Bacteria, Proteobacteria, Gammaproteobacteria, Xanthomonadales, Xanthomonadaceae, Luteibacter	5.36	CP	5.01	0.05
Bacteria, WS6	0.00			-
Bacteria, Proteobacteria, Betaproteobacteria, Burkholderiales, Alcaligenaceae, Pusillimonas	5.29			-
Bacteria, Actinobacteria, Actinobacteria, Kineosporiales, Kineosporiaceae, Angustibacter	5.69	CK	5.14	0.05
Bacteria, Bacteroidetes, Flavobacteriia, Flavobacteriales, NS9_marine_group	5.11	CP	4.82	0.04
Bacteria, Actinobacteria, Actinobacteria, Streptomycetales	6.59			-

Bacteria, Bacteroidetes, Sphingobacteriia, Sphingobacteriales, Chitinophagaceae, Arachidicoccus	7.08			-
Bacteria, Bacteroidetes, Flavobacteriia, Flavobacteriales, Flavobacteriaceae, Vitellibacter	4.28			-
Bacteria, Proteobacteria, Betaproteobacteria, Burkholderiales, Burkholderiaceae, Pandoraea	5.17			-
Bacteria, Proteobacteria, Betaproteobacteria, Burkholderiales, Comamonadaceae, Aquabacterium	4.18			-
Bacteria, Proteobacteria, Betaproteobacteria, Burkholderiales, Comamonadaceae, Variovorax	4.61			-
Bacteria, Verrucomicrobia, Spartobacteria, Chthoniobacterales, Chthoniobacteraceae	5.08			-
Bacteria, Firmicutes, Bacilli, Bacillales, Bacillaceae, Bacillus	6.13			-
Bacteria, Proteobacteria, Betaproteobacteria, Rhodocyclales, Rhodocyclaceae, Uliginosibacterium	0.00			-
Bacteria, Bacteroidetes, Sphingobacteriia, Sphingobacteriales, Chitinophagaceae	7.10			-
Bacteria, Chloroflexi, Ktedonobacteria, Ktedonobacterales, G12_WMSP1	5.21			-
Bacteria, Proteobacteria, Alphaproteobacteria, Caulobacterales, Hyphomonadaceae, Hirschia	5.04			-
Bacteria, Bacteroidetes, Cytophagia, Cytophagales, Cytophagaceae, Sporocytophaga	5.23			-
Bacteria, Proteobacteria, Gammaproteobacteria, Pseudomonadales	6.24	CP	5.84	0.05
Bacteria, Actinobacteria, Actinobacteria, Streptosporangiales, Streptosporangiaceae	6.02	CK	5.58	0.05
Bacteria, Planctomycetes, Planctomycetacia, Planctomycetales, Planctomycetaceae, Planctomyces	4.23			-
Bacteria, Proteobacteria, Alphaproteobacteria, Rhodospirillales, DA111	6.49			-
Bacteria, Actinobacteria, Actinobacteria, Pseudonocardiales, Pseudonocardiaceae, Saccharothrix	0.00			-

Bacteria, Proteobacteria, Alphaproteobacteria, Rhizobiales, Xanthobacteraceae	6.24			-
Bacteria, Bacteroidetes, Flavobacteriia, Flavobacteriales, Flavobacteriaceae, Galbibacter	3.68			-
Bacteria, Proteobacteria, Gammaproteobacteria, Xanthomonadales, Xanthomonadales_Incertae_Sedis, Acidibacter	6.33			-
Bacteria, Chloroflexi, Anaerolineae, Anaerolineales, Anaerolineaceae, Anaerolinea	4.34			-
Bacteria, Actinobacteria, Actinobacteria, Micromonosporales, Micromonosporaceae, Micromonospora	5.79			-
Bacteria, Proteobacteria, Alphaproteobacteria, Rickettsiales, Rickettsiaceae	0.00			-
Bacteria, Firmicutes, Clostridia, Clostridiales, Christensenellaceae	3.58			-
Bacteria, Proteobacteria, Alphaproteobacteria, Rhodospirillales, AKYH478	4.27			-
Bacteria, Acidobacteria, Acidobacteria, Vicinamibacter	4.46			-
Bacteria, Actinobacteria, Actinobacteria, Catenulisporales, Actinospicaceae, Actinospica	5.62			-
Bacteria, Ignavibacteriae, Ignavibacteria, Ignavibacteriales, BSV26	4.98			-
Bacteria, Elusimicrobia, Elusimicrobia	5.46	CK	5.07	0.05
Bacteria, Proteobacteria, Betaproteobacteria, Burkholderiales, Comamonadaceae	5.80			-
Bacteria, Proteobacteria, Gammaproteobacteria, Enterobacteriales, Enterobacteriaceae, _Serratia__fonticola_RB_25_group	0.00			-
Bacteria, Bacteroidetes, Cytophagia, Cytophagales, Cytophagaceae, Ohtaekwangia	5.26	CP	4.93	0.05
Bacteria, Proteobacteria, Gammaproteobacteria, Oceanospirillales, Oceanospirillaceae, Pseudohongiella	5.36			-
Bacteria, Actinobacteria, Actinobacteria, Streptosporangiales	6.15			-

Bacteria, Acidobacteria, Acidobacteria, Acidobacteriales, Acidobacteriaceae__Subgroup_1, Edaphobacter	5.70	CK	5.17	0.05
Bacteria, Acidobacteria, Acidobacteria, Holophagales, Holophagaceae	6.21	CP	5.84	0.05
Bacteria, Firmicutes, Bacilli, Bacillales, Bacillaceae	6.14			-
Bacteria, Actinobacteria, Actinobacteria, Frankiales, Sporichthyaceae	5.00	CP	4.47	0.05
Bacteria, Firmicutes, Bacilli, Bacillales, Planococcaceae	5.83			-
Bacteria, Firmicutes, Bacilli, Bacillales, Planococcaceae, Solibacillus	5.31			-
Bacteria, Proteobacteria, Alphaproteobacteria, Rhodospirillales, Acetobacteraceae, Rhodovastum	5.90			-
Bacteria, Proteobacteria, Betaproteobacteria, Hydrogenophilales, Hydrogenophilaceae, Thiobacillus	4.86			-
Bacteria, Firmicutes, Bacilli, Bacillales, Sporolactobacillaceae, Tuberibacillus	5.69	CK	5.36	0.04
Bacteria, Actinobacteria, Actinobacteria, Micrococcales, Intrasporangiaceae, Terrabacter	5.60	CP	4.96	0.05
Bacteria, Chloroflexi, Elev_1554	3.88			-
Bacteria, Proteobacteria, Gammaproteobacteria, Oceanospirillales, Halomonadaceae	4.77			-
Bacteria, Actinobacteria, Actinobacteria, Micrococcales, Micrococcaceae	6.30			-
Bacteria, Chloroflexi, Thermomicrobia	6.31	CK	5.53	0.05
Bacteria, Proteobacteria, Deltaproteobacteria, Desulfurellales	6.06			-
Bacteria, Spirochaetae, Spirochaetes	4.84			-
Bacteria, Chloroflexi, Thermomicrobia, AKYG1722	5.36			-

Bacteria, Bacteroidetes, Sphingobacteriia, Sphingobacteriales, Chitinophagaceae, Flavitalea	5.37			-
Bacteria, Actinobacteria, Actinobacteria, Frankiales, Frankiaceae, Jatrophihabitans	6.17			-
Bacteria, Proteobacteria, Gammaproteobacteria, Pseudomonadales, Moraxellaceae, Psychrobacter	4.18			-
Bacteria, Planctomycetes, Planctomycetacia, Planctomycetales, Planctomycetaceae, Singulisphaera	5.65			-
Bacteria, Firmicutes, Bacilli, Bacillales, Thermoactinomycetaceae, Shimazuella	3.88			-
Bacteria, Actinobacteria, Actinobacteria, Micrococcales, Microbacteriaceae, Lysinimonas	6.20			-
Bacteria, Firmicutes, Bacilli, C178B	6.18	CK	5.82	0.05
Bacteria, Firmicutes, Clostridia, Clostridiales, Peptostreptococcaceae	5.30			-
Bacteria, Proteobacteria, Betaproteobacteria, Burkholderiales, Burkholderiaceae, Cupriavidus	4.91			-
Bacteria, Proteobacteria, Gammaproteobacteria, Xanthomonadales, Xanthomonadaceae, Dyella	6.92			-
Bacteria, Verrucomicrobia	6.63			-
Bacteria, Actinobacteria, Actinobacteria, Solirubrobacterales, Gsoil_1167	4.50			-
Bacteria, Proteobacteria, Betaproteobacteria, Burkholderiales, Burkholderiaceae, Burkholderia_Paraburkholderia	6.62			-
Bacteria, Acidobacteria, Acidobacteria, Solibacterales, Solibacteraceae__Subgroup_3_, Candidatus_Solibacter	6.40			-
Bacteria, Proteobacteria, Deltaproteobacteria, Myxococcales, Phaselicystidaceae	4.16			-
Bacteria, Actinobacteria, Actinobacteria, Frankiales, Nakamurellaceae	5.65	CP	5.14	0.05
Bacteria, Actinobacteria, Actinobacteria, Catenulisporales	5.85			-

Bacteria, Proteobacteria, Alphaproteobacteria, Rhodospirillales, Rhodospirillales_Incertae_Sedis	6.26			-
Bacteria, Firmicutes, Clostridia, Clostridiales, Lachnospiraceae, Lachnospiraceae_NK3A20_group	4.23			-
Bacteria, Planctomycetes, OM190	3.68			-
Bacteria, Proteobacteria, Betaproteobacteria, TRA3_20	5.92			-
Bacteria, Proteobacteria, Betaproteobacteria, Burkholderiales, Comamonadaceae, Pelomonas	4.89			-
Bacteria, Planctomycetes, Planctomycetacia, Planctomycetales, Planctomycetaceae, Isosphaera	5.10			-
Bacteria, Actinobacteria, Actinobacteria, Micrococcales, Bogoriellaceae, Georgenia	4.39			-
Bacteria, Bacteroidetes, Sphingobacteriia, Sphingobacteriales, env_OPS_17	5.74	CP	5.39	0.05
Bacteria, Proteobacteria, Gammaproteobacteria, Legionellales, Coxiellaceae	5.84			-
Bacteria, Bacteroidetes, Flavobacteriia, Flavobacteriales, Flavobacteriaceae, Gelidibacter	5.25			-
Bacteria, Proteobacteria, Gammaproteobacteria, Xanthomonadales, Xanthomonadaceae, Stenotrophomonas	5.63			-
Bacteria, Proteobacteria, Betaproteobacteria, Burkholderiales, Alcaligenaceae, Verticia	4.26			-
Bacteria, Proteobacteria, Alphaproteobacteria, Rhizobiales, Beijerinckiaceae, Methylovirgula	5.53			-
Bacteria, Actinobacteria, Actinobacteria, Frankiales	6.29			-
Bacteria, Chloroflexi, Chloroflexia, Chloroflexales, Roseiflexaceae, Roseiflexus	5.97	CP	5.60	0.05
Bacteria, Proteobacteria, Alphaproteobacteria, Rhizobiales, Rhizobiaceae	6.02			-
Bacteria, Proteobacteria, Alphaproteobacteria, Rhizobiales, Roseiarcaceae, Roseiarcus	5.91			-

Bacteria, Proteobacteria, Gammaproteobacteria, Cellvibrionales	5.12			-
Bacteria, Firmicutes, Bacilli, Bacillales, Paenibacillaceae, Paenibacillus	6.41			-
Bacteria, Proteobacteria, Deltaproteobacteria, Myxococcales, Eel_36e1D6	3.97			-
Bacteria, Proteobacteria, Alphaproteobacteria, Rhizobiales, Bradyrhizobiaceae	5.40			-
Bacteria, Acidobacteria, Acidobacteria, Blastocatellales, Blastocatellaceae__Subgroup_4, Tellurimicrobium	4.58			-
Bacteria, Actinobacteria, Actinobacteria, Solirubrobacterales, Patulibacteraceae, Patulibacter	4.64			-
Bacteria, Actinobacteria, Actinobacteria, Propionibacteriales, Nocardioidaceae, Aeromicrobium	5.68	CP	5.21	0.05
Bacteria, Proteobacteria, Betaproteobacteria, Nitrosomonadales	6.83			-
Bacteria, Proteobacteria, Alphaproteobacteria, Rhizobiales, Methylobacteriaceae	4.99			-
Bacteria, Bacteroidetes, Cytophagia, Cytophagales, Cytophagaceae, Cytophaga	5.20			-
Bacteria, Actinobacteria, Actinobacteria, Frankiales, Sporichthyaceae, Sporichthya	4.67			-
Bacteria, Spirochaetae, Spirochaetes, Spirochaetales, Leptospiraceae	4.46			-
Bacteria, Bacteroidetes, Sphingobacteriia, Sphingobacteriales, Chitinophagaceae, Terrimonas	5.83			-
Bacteria, Proteobacteria, Alphaproteobacteria, Rhizobiales, Brucellaceae	4.85			-
Bacteria, Proteobacteria, Deltaproteobacteria, Bradymonadales	4.20			-
Bacteria, Planctomycetes, Planctomycetacia, Brocadiales	4.12			-
*Bacteria, Acidobacteria, Acidobacteria, Solibacterales, Solibacteraceae__Subgroup_3_*	6.83			-

Bacteria, Proteobacteria, Alphaproteobacteria, Rhizobiales, alphaI_cluster	4.82			-
Bacteria, Proteobacteria, Deltaproteobacteria, Bdellovibrionales, Bacteriovoracaceae, Peredibacter	5.10	CP	4.68	0.05
Bacteria, Armatimonadetes, Fimbriimonadia, Fimbriimonadales	5.35			-
Bacteria, Acidobacteria, Acidobacteria, Acidobacteriales, Acidobacteriaceae__Subgroup_1, Acidobacterium	6.25			-
Bacteria, Acidobacteria, Acidobacteria, Solibacterales, Solibacteraceae__Subgroup_3_, Bryobacter	6.63			-
Bacteria, Actinobacteria, Actinobacteria, Corynebacteriales, Nocardiaceae, Nocardia	5.02			-
Bacteria, Spirochaetae, Spirochaetes, Spirochaetales, Spirochaetaceae	4.69			-
Bacteria, Chlamydiae, Chlamydiae, Chlamydiales, Parachlamydiaceae, Neochlamydia	4.25			-
Bacteria, Firmicutes, Bacilli, Bacillales, Alicyclobacillaceae, Tumebacillus	5.37			-
Bacteria, Parcubacteria, Candidatus_Nomurabacteria	3.68			-
Bacteria, Bacteroidetes, Sphingobacteriia, Sphingobacteriales, Sphingobacteriaceae	5.33			-
Bacteria, Bacteroidetes, Sphingobacteriia, Sphingobacteriales, Chitinophagaceae, Chitinophaga	5.87			-
Bacteria, Proteobacteria, Alphaproteobacteria, Rhizobiales, KF_JG30_B3	5.40			-
Bacteria, Proteobacteria, Betaproteobacteria, Methylophilales	5.75			-
Bacteria, Proteobacteria, Gammaproteobacteria, Xanthomonadales	5.83			-
Bacteria, Proteobacteria, Alphaproteobacteria, Rhizobiales, Methylobacteriaceae, Meganema	4.57			-
Bacteria, Proteobacteria, Alphaproteobacteria, Rhodobacterales, Rhodobacteraceae	3.82			-

Bacteria, Bacteroidetes, Cytophagia, Cytophagales, Cytophagaceae, Adhaeribacter	3.97			-
Bacteria, Armatimonadetes, Chthonomonadetes	4.28			-
Bacteria, Actinobacteria, Actinobacteria, Corynebacteriales, Nocardiaceae, Smaragdicoccus	4.53	CP	4.58	0.04
Bacteria, Proteobacteria, Alphaproteobacteria, Rhizobiales, Beijerinckiaceae	5.59			-
Bacteria, Proteobacteria, Alphaproteobacteria	5.32			-
Bacteria, Proteobacteria, Alphaproteobacteria, Rhodospirillales, Rhodospirillaceae	6.14			-
Bacteria, Proteobacteria, Alphaproteobacteria, Rhizobiales, Hyphomicrobiaceae, Hyphomicrobium	5.85			-
Bacteria, Proteobacteria, Gammaproteobacteria, Aeromonadales, Aeromonadaceae, Aeromonas	4.62	CP	4.58	0.04
Bacteria, Firmicutes, Clostridia, Clostridiales, Family_XIII, Mogibacterium	0.00			-
Bacteria, Proteobacteria, ARKICE_90	4.49	CP	4.96	0.04
Bacteria, Proteobacteria, Alphaproteobacteria, Caulobacterales, Hyphomonadaceae, Woodsholea	5.01			-
Bacteria, Proteobacteria, Alphaproteobacteria, Caulobacterales, Caulobacteraceae, Caulobacter	5.11			-
Bacteria, Proteobacteria, Gammaproteobacteria, Xanthomonadales, Xanthomonadaceae, Tahibacter	4.69	CP	4.72	0.05
Bacteria, Chloroflexi, Ktedonobacteria, B12_WMSP1	5.60			-
Bacteria, Proteobacteria, Betaproteobacteria, Methylophilales, Methylophilaceae	5.35			-
Bacteria, Proteobacteria, Deltaproteobacteria, Bdellovibrionales, Bacteriovoracaceae	0.00			-
Bacteria, Proteobacteria, Betaproteobacteria	4.76			-

Bacteria, Actinobacteria, Actinobacteria, Corynebacteriales	6.17			-
Bacteria, Proteobacteria, Alphaproteobacteria, Caulobacterales, Caulobacteraceae	6.06			-
Bacteria, Proteobacteria, Alphaproteobacteria, Rhizobiales, Xanthobacteraceae, Labrys	4.48			-
*Bacteria, TM6__Dependentiae_*	5.13			-
Bacteria, Proteobacteria, Betaproteobacteria, Burkholderiales, Burkholderiaceae, Limnobacter	5.18			-
Bacteria, Deinococcus_Thermus, Deinococci, Deinococcales, Deinococcaceae	3.93			-
Bacteria, Bacteroidetes, Cytophagia, Cytophagales, Cytophagaceae, Leadbetterella	0.00			-
Bacteria, Proteobacteria, Deltaproteobacteria, Oligoflexales, 0319_6G20	5.85			-
Bacteria, Parcubacteria, Candidatus_Azambacteria	3.97			-
Bacteria, Latescibacteria	5.76	CP	5.28	0.05
Bacteria, Armatimonadetes	4.21			-
Bacteria, Firmicutes	7.32			-
Bacteria, Ignavibacteriae	4.98			-
Bacteria, Bacteroidetes, Flavobacteriia, Flavobacteriales, Flavobacteriaceae, Chryseobacterium	5.57			-
Bacteria, GAL15	5.90			-
Bacteria, Planctomycetes, Phycisphaerae, Tepidisphaerales, Tepidisphaeraceae	6.10			-
Bacteria, Chloroflexi, S085	5.48	CP	5.10	0.05

Bacteria, Acidobacteria, Acidobacteria, Blastocatellales, Blastocatellaceae__Subgroup_4, Stenotrophobacter	4.35			-
Bacteria, Bacteroidetes, Sphingobacteriia, Sphingobacteriales, Chitinophagaceae, Filimonas	5.28			-
Bacteria, Planctomycetes, Phycisphaerae, Phycisphaerales, Phycisphaeraceae, SM1A02	4.55			-
Bacteria, Chloroflexi, Thermomicrobia, Sphaerobacterales, Sphaerobacteraceae, Nitrolancea	5.86	CK	5.21	0.05
Bacteria, Firmicutes, Clostridia, Clostridiales, Lachnospiraceae	4.23			-
Bacteria, Bacteroidetes, Bacteroidia, Bacteroidales, Porphyromonadaceae, Parabacteroides	0.00			-
Bacteria, Proteobacteria, Alphaproteobacteria, Rhizobiales, Xanthobacteraceae, Pseudoxanthobacter	4.25			-
Bacteria, Firmicutes, Limnochordia	4.70	CK	4.62	0.04
Bacteria, Fibrobacteres, Fibrobacteria, Fibrobacterales	4.93			-
Bacteria, Proteobacteria, Alphaproteobacteria, Rickettsiales, SM2D12	5.47			-
Bacteria, Proteobacteria, Betaproteobacteria, Neisseriales, Neisseriaceae, Vitreoscilla	0.00			-
Bacteria, Firmicutes, Clostridia, Halanaerobiales	6.62			-
Bacteria, Proteobacteria, Deltaproteobacteria, Myxococcales, Haliangiaceae	6.08			-
Bacteria, Gemmatimonadetes, Gemmatimonadetes, Longimicrobiales, Longimicrobiaceae	5.07			-
Bacteria, Chlamydiae	5.05			-
Bacteria, Actinobacteria, Actinobacteria, Micrococcales, Micrococcaceae, Arthrobacter	5.96	CP	5.47	0.05
Bacteria, Actinobacteria, Actinobacteria, Frankiales, Frankiaceae	6.17			-

Bacteria, Proteobacteria, Gammaproteobacteria, Legionellales, Coxiellaceae, Coxiella	4.50	CK	4.69	0.04
Bacteria, Firmicutes, Erysipelotrichia, Erysipelotrichales	5.32			-
Bacteria, Proteobacteria, Alphaproteobacteria, Rhizobiales, Phyllobacteriaceae, Mesorhizobium	6.48			-
Bacteria, Proteobacteria, Deltaproteobacteria, Myxococcales, Polyangiaceae	4.16			-
Bacteria, Proteobacteria, Alphaproteobacteria, Rhizobiales, Methylocystaceae	4.56			-
Bacteria, Actinobacteria, Actinobacteria, Frankiales, Geodermatophilaceae, Blastococcus	5.74			-
Bacteria, Proteobacteria, Deltaproteobacteria, Myxococcales, Myxococcaceae	4.63	CP	4.42	0.05
Bacteria, Proteobacteria, Deltaproteobacteria, Myxococcales, Phaselicystidaceae, Phaselicystis	4.16			-
Bacteria, Bacteroidetes, Cytophagia, Cytophagales, Cytophagaceae, Chryseolinea	5.12			-
Bacteria, Acidobacteria, Acidobacteria, Acidobacteriales, Acidobacteriaceae__Subgroup_1, Candidatus_Koribacter	5.37			-
Bacteria, Proteobacteria, Alphaproteobacteria, Rhodospirillales, MNC12	5.06	CP	4.80	0.04
Bacteria, Proteobacteria, Gammaproteobacteria, Oceanospirillales, Hahellaceae	3.82			-
Bacteria, Firmicutes, Bacilli, Bacillales, Planococcaceae, Lysinibacillus	5.42			-
Bacteria, Proteobacteria, Alphaproteobacteria, Rhizobiales, Rhizobiales_Incertae_Sedis, Agaricicola	5.33			-
Bacteria, Firmicutes, Bacilli, Lactobacillales, Carnobacteriaceae, Carnobacterium	4.44			-
Bacteria, Proteobacteria, Betaproteobacteria, Burkholderiales, Alcaligenaceae, Eoetvoesia	5.27			-
Bacteria, Actinobacteria, Actinobacteria, Streptosporangiales, Streptosporangiaceae, Nonomuraea	4.75			-

Bacteria, Bacteroidetes, Sphingobacteriia, Sphingobacteriales, Sphingobacteriaceae, Pedobacter	5.93			-
Bacteria, Gemmatimonadetes	7.35			-
Bacteria, Parcubacteria	6.41			-
Bacteria, Acidobacteria, Acidobacteria, Blastocatellales	6.26			-
Bacteria, Proteobacteria, Deltaproteobacteria	3.97			-
Bacteria, Proteobacteria, Alphaproteobacteria, Rhizobiales, Bradyrhizobiaceae, Bradyrhizobium	6.60			-
Bacteria, Actinobacteria, Actinobacteria, Solirubrobacterales, FFCH11085	4.56			-
Bacteria, Actinobacteria, Actinobacteria, Frankiales, Acidothermaceae	6.18			-
Bacteria, Proteobacteria, Deltaproteobacteria, Bdellovibrionales, Bdellovibrionaceae	5.77			-
Bacteria, Proteobacteria, Gammaproteobacteria, Pseudomonadales, Moraxellaceae, Perlucidibaca	0.00			-
Bacteria, Proteobacteria, Alphaproteobacteria, Rhizobiales, Beijerinckiaceae, Methylorosula	4.69			-
Bacteria, Actinobacteria, Actinobacteria, Acidimicrobiales, Iamiaceae, Iamia	5.28			-
Bacteria, Actinobacteria, Actinobacteria, Acidimicrobiales, OM1_clade	3.82			-
Bacteria, Proteobacteria, Betaproteobacteria, Nitrosomonadales, Nitrosomonadaceae	6.81			-
Bacteria, Proteobacteria, Gammaproteobacteria, Legionellales, Coxiellaceae, Rickettsiella	4.89			-
Bacteria, Proteobacteria, Alphaproteobacteria, Rhizobiales, Rhizobiaceae, Kaistia	4.95			-
Bacteria, Bacteroidetes, Cytophagia, Cytophagales	6.54	CP	6.11	0.05

Bacteria, Chloroflexi, Thermomicrobia, JG30_KF_CM45	6.04			-
Bacteria, Bacteroidetes, Sphingobacteriia, Sphingobacteriales, Chitinophagaceae, Parasegetibacter	4.57			-
Bacteria, Proteobacteria, Alphaproteobacteria, Caulobacterales	6.45			-
Bacteria, Bacteroidetes, Cytophagia, Cytophagales, Cytophagaceae	6.43	CP	6.06	0.05
Bacteria, Proteobacteria, Alphaproteobacteria, Rhodospirillales, MND8	4.44			-
Bacteria, Proteobacteria, Deltaproteobacteria, NB1_j	4.44			-
Bacteria, Proteobacteria, Betaproteobacteria, Burkholderiales, Alcaligenaceae, Paralcaligenes	5.77	CP	5.35	0.05
Bacteria, Proteobacteria, Alphaproteobacteria, Rhizobiales, Rhizobiales_Incertae_Sedis, Nordella	5.77	CP	5.37	0.05
Bacteria, FCPU426	4.67			-
Bacteria, Firmicutes, Bacilli, Bacillales, Paenibacillaceae, Aneurinibacillus	3.93			-
Bacteria, Proteobacteria, Alphaproteobacteria, Rhizobiales, Bradyrhizobiaceae, Bosea	4.77	CP	4.57	0.05
Bacteria, Proteobacteria, Betaproteobacteria, Burkholderiales, Comamonadaceae, Rhizobacter	4.87			-
Bacteria, Proteobacteria, Gammaproteobacteria, Cellvibrionales, Cellvibrionaceae, Simiduia	3.97			-
Bacteria, Proteobacteria, Deltaproteobacteria, Myxococcales, Blfdi19	4.72			-
Bacteria, Actinobacteria, Actinobacteria, Acidimicrobiales	6.22			-
Bacteria, Armatimonadetes, Chthonomonadetes, Chthonomonadales	4.28			-
Bacteria, Proteobacteria, Betaproteobacteria, Methylophilales, Methylophilaceae, Methylotenera	5.36			-

Bacteria, Proteobacteria, Deltaproteobacteria, Oligoflexales	5.96			-
Bacteria, Proteobacteria, Betaproteobacteria, Rhodocyclales	5.16			-
Bacteria, Fibrobacteres, Fibrobacteria	4.93			-
Bacteria, Proteobacteria, Gammaproteobacteria, Xanthomonadales, Xanthomonadaceae, Rhodanobacter	7.84			-
Bacteria, Proteobacteria, Betaproteobacteria, Nitrosomonadales, Nitrosomonadaceae, Nitrosospira	5.78			-
Bacteria, Proteobacteria, Deltaproteobacteria, Desulfuromonadales, Geobacteraceae, Geobacter	3.97			-
Bacteria, Bacteroidetes, Sphingobacteriia, Sphingobacteriales, Chitinophagaceae, Flavihumibacter	4.69	CP	4.58	0.05
Bacteria, Firmicutes, Bacilli, Bacillales, Thermoactinomycetaceae	3.88			-
Bacteria, Peregrinibacteria	3.97			-
Bacteria, Proteobacteria, Gammaproteobacteria	6.51			-
Bacteria, Actinobacteria, Actinobacteria	5.78			-
Bacteria, Chloroflexi, SBR2076	5.54			-
Bacteria, Parcubacteria, Candidatus_Jorgensenbacteria	4.26			-
Bacteria, Proteobacteria, Alphaproteobacteria, Rhodospirillales, Rhodospirillaceae, Dongia	5.66			-
Bacteria, Acidobacteria	7.55			-
Bacteria, Chloroflexi, Chloroflexia, Chloroflexales, FFCH7168	4.57			-
Bacteria, Actinobacteria, Actinobacteria, Acidimicrobiales, Acidimicrobiales_Incertae_Sedis	0.00			-

Bacteria, Proteobacteria, Betaproteobacteria, Hydrogenophilales, Hydrogenophilaceae	4.86			-
Bacteria, Actinobacteria, Actinobacteria, Micromonosporales, Micromonosporaceae, Dactylosporangium	5.06			-
Bacteria, Cyanobacteria, Cyanobacteria, SubsectionIII, FamilyI_o__SubsectionIII	5.06	CP	4.76	0.05
Bacteria, Actinobacteria, Actinobacteria, Micrococcales, Intrasporangiaceae	6.31			-
Bacteria, Proteobacteria, Gammaproteobacteria, Oceanospirillales, Oceanospirillaceae	5.36			-
Bacteria, Bacteroidetes, Flavobacteriia, Flavobacteriales, Flavobacteriaceae, Aequorivita	4.97			-
Bacteria, Chlamydiae, Chlamydiae, Chlamydiales, cvE6	4.94			-
Bacteria, Firmicutes, Limnochordia, Limnochordales, Limnochordaceae	4.70	CK	4.62	0.04
Bacteria, Proteobacteria, Gammaproteobacteria, Xanthomonadales, Xanthomonadaceae, Thermomonas	4.99			-
Bacteria, Ignavibacteriae, Ignavibacteria	4.98			-
Bacteria, Proteobacteria, Deltaproteobacteria, Oligoflexales, Oligoflexaceae	5.30			-
Bacteria, Proteobacteria, Deltaproteobacteria, Myxococcales, 27F_1492R	4.48			-
Bacteria, Fibrobacteres, Fibrobacteria, Fibrobacterales, Fibrobacteraceae	4.76	CP	4.62	0.04
Bacteria, Actinobacteria, Actinobacteria, Propionibacteriales, Nocardioidaceae, Marmoricola	6.36	CP	5.87	0.05
Bacteria, Actinobacteria, Actinobacteria, Micrococcales, Microbacteriaceae, Leucobacter	3.58			-
Bacteria, Chloroflexi, Ktedonobacteria, B10_SB3A	5.20			-
Bacteria, Chloroflexi, Caldilineae, Caldilineales, Caldilineaceae	4.52			-

Bacteria, Bacteroidetes, Sphingobacteriia, Sphingobacteriales, Sphingobacteriaceae, Pseudopedobacter	5.37	CP	5.15	0.04
Bacteria, Saccharibacteria, Candidatus_Saccharimonas	4.12			-
Bacteria, Actinobacteria, Actinobacteria, Pseudonocardiales, Pseudonocardiaceae, Amycolatopsis	5.41			-
Bacteria, Acidobacteria, Acidobacteria, Acidobacteriales, Acidobacteriaceae__Subgroup_1	7.12			-
Bacteria, Firmicutes, Clostridia, Clostridiales, Family_XIII	0.00			-
Bacteria, Actinobacteria, Actinobacteria, Solirubrobacterales	4.25			-
Bacteria, Actinobacteria, Actinobacteria, Micrococcales, Microbacteriaceae, Leifsonia	5.74			-
Bacteria, Actinobacteria, Actinobacteria, Pseudonocardiales, Pseudonocardiaceae	5.67			-
Bacteria, Actinobacteria, Actinobacteria, Micromonosporales, Micromonosporaceae, Hamadaea	4.82			-
Bacteria, Acidobacteria, Acidobacteria, Blastocatellales, Blastocatellaceae__Subgroup_4, RB41	5.51	CP	5.09	0.05
Bacteria, Acidobacteria, Acidobacteria, Acidobacteriales	7.22			-
Bacteria, Nitrospirae, Nitrospira	6.36			-
Bacteria, Armatimonadetes, Chthonomonadetes, Chthonomonadales, Chthonomonadaceae, Chthonomonas	4.28			-
Bacteria, Actinobacteria, Actinobacteria, Micrococcales, Microbacteriaceae, Humibacter	5.96			-
Bacteria, Acidobacteria, Acidobacteria, Solibacterales, Solibacteraceae__Subgroup_3_, Paludibaculum	4.94			-
Bacteria, Fibrobacteres	4.93			-
Bacteria, Verrucomicrobia, Verrucomicrobiae, Verrucomicrobiales, Verrucomicrobiaceae, Verrucomicrobium	0.00			-

Bacteria, Proteobacteria, Gammaproteobacteria, Xanthomonadales, Xanthomonadaceae, Frateuria	6.30			-
Bacteria, Proteobacteria, Deltaproteobacteria, Bradymonadales, Bradymonadaceae, Bradymonas	4.20			-
Bacteria, Spirochaetae, Spirochaetes, Spirochaetales, Leptospiraceae, Turneriella	4.46			-
Bacteria, Cyanobacteria	6.07			-
Bacteria, Proteobacteria, Alphaproteobacteria, Rhizobiales, Hyphomicrobiaceae, Rhodomicrobium	4.63	CP	4.61	0.05
Bacteria, Actinobacteria, Actinobacteria, Kineosporiales, Kineosporiaceae	5.69	CK	5.20	0.05
Bacteria, Proteobacteria, Gammaproteobacteria, Thiotrichales, Thiotrichaceae, Beggiatoa	0.00			-
Bacteria, Bacteroidetes, Flavobacteriia, Flavobacteriales, Flavobacteriaceae, Moheibacter	4.95			-
Bacteria, Proteobacteria, Alphaproteobacteria, Sphingomonadales, Sphingomonadaceae	7.43			-
Bacteria, Proteobacteria, Alphaproteobacteria, Rhizobiales, Bradyrhizobiaceae, Rhodopseudomonas	5.53			-
Bacteria, Proteobacteria, Alphaproteobacteria, Rhodospirillales, Rhodospirillaceae, Ferrovibrio	3.93			-
Bacteria, Chloroflexi, Ktedonobacteria, Ktedonobacterales	5.32			-
Bacteria, Bacteroidetes, Flavobacteriia	6.64	CP	6.21	0.05
Bacteria, Proteobacteria, Alphaproteobacteria, Rhodospirillales, Rhodospirillaceae, Inquilinus	4.88			-
Bacteria, Proteobacteria, Betaproteobacteria, Nitrosomonadales, Nitrosomonadaceae, Nitrosomonas	4.38			-
Bacteria, Actinobacteria, Actinobacteria, Micromonosporales	6.02			-
Bacteria, Firmicutes, Bacilli, Bacillales, Alicyclobacillaceae, Alicyclobacillus	4.96			-

Bacteria, Bacteroidetes, Flavobacteriia, Flavobacteriales, Flavobacteriaceae, Flavobacterium	6.07			-
Bacteria, Proteobacteria, Alphaproteobacteria, Sphingomonadales, DSSF69	4.69			-
Bacteria, Proteobacteria, Gammaproteobacteria, Enterobacteriales, Enterobacteriaceae	5.46	CP	5.06	0.05
Bacteria, Acidobacteria, Acidobacteria, Holophagales, Holophagaceae, Geothrix	4.53			-
Bacteria, Proteobacteria, Betaproteobacteria, Burkholderiales, Comamonadaceae, Ottowia	5.93	CP	5.56	0.05
Bacteria, Bacteroidetes, Bacteroidia, Bacteroidales	5.09			-
Bacteria, Elusimicrobia	5.46	CK	5.02	0.05
Bacteria, Proteobacteria, Betaproteobacteria, B1_7BS	3.93			-
Bacteria, Proteobacteria, Alphaproteobacteria, Rhizobiales, Hyphomicrobiaceae, Pedomicrobium	5.04	CP	4.68	0.05
Bacteria, Actinobacteria, Actinobacteria, Solirubrobacterales, Solirubrobacteraceae	3.93			-
Bacteria, Proteobacteria, Gammaproteobacteria, Enterobacteriales, Enterobacteriaceae, Serratia	3.82			-
Bacteria, Chloroflexi, JG30_KF_CM66	5.76			-
Bacteria, Actinobacteria, Actinobacteria, Streptosporangiales, Streptosporangiaceae, Sphaerisporangium	4.98			-
Bacteria, BRC1	4.68			-
Bacteria, Planctomycetes	6.46			-
Bacteria, Proteobacteria, Alphaproteobacteria, Rhodospirillales, Acetobacteraceae, Acidicaldus	5.02			-
Bacteria, Chloroflexi, Thermomicrobia, Sphaerobacterales, Sphaerobacteraceae	5.86	CK	5.17	0.05

Bacteria, Actinobacteria, Actinobacteria, Micrococcales, Demequinaceae	3.97			-
Bacteria, Actinobacteria, Actinobacteria, Frankiales, Nakamurellaceae, Nakamurella	5.65	CP	5.12	0.05
Bacteria, Firmicutes, Clostridia, Clostridiales, Family_XVII	4.38			-
Bacteria, Actinobacteria, Actinobacteria, Solirubrobacterales, TM146	5.13	CP	4.75	0.05
Bacteria, Proteobacteria, Gammaproteobacteria, HTA4	4.67			-
Bacteria, Proteobacteria, Alphaproteobacteria, Rhizobiales, Roseiarcaceae	5.91			-
Bacteria, Proteobacteria, Gammaproteobacteria, Legionellales	5.87			-
Bacteria, Chloroflexi, KD4_96	6.45			-
Bacteria, Firmicutes, Bacilli, Lactobacillales, Enterococcaceae	6.03			-
Bacteria, Actinobacteria, Actinobacteria, Streptosporangiales, Thermomonosporaceae, Actinomadura	5.57			-
Bacteria, Actinobacteria, Actinobacteria, Micrococcales	7.00			-
Bacteria, Proteobacteria, Alphaproteobacteria, Caulobacterales, Caulobacteraceae, Phenylobacterium	5.89			-
Bacteria, Armatimonadetes, Fimbriimonadia	5.35			-
Bacteria, Actinobacteria, Actinobacteria, Acidimicrobiales, Acidimicrobiaceae, Ilumatobacter	4.82			-
Bacteria, Chloroflexi, Ktedonobacteria, Ktedonobacterales, HSB_OF53_F07	6.40			-
Bacteria, Proteobacteria, Gammaproteobacteria, Oceanospirillales, Halomonadaceae, Halomonas	4.77			-
Bacteria, Proteobacteria, Alphaproteobacteria, Rhodospirillales, JG37_AG_20	5.77			-

Bacteria, Microgenomates	5.46			-
Bacteria, Proteobacteria, JTB23	4.35			-
Bacteria, Proteobacteria, Deltaproteobacteria, Myxococcales, Haliangiaceae, Haliangium	6.08			-
Bacteria, Parcubacteria, Candidatus_Magasanikbacteria	5.09			-
Bacteria, Verrucomicrobia, Spartobacteria, Chthoniobacterales, Chthoniobacteraceae, Chthoniobacter	5.08			-
Bacteria, Actinobacteria, Actinobacteria, Streptomycetales, Streptomycetaceae	6.41			-
Bacteria, Firmicutes, Bacilli	3.58			-
Bacteria, Proteobacteria, Gammaproteobacteria, Xanthomonadales, Solimonadaceae, Solimonas	5.57	CP	5.27	0.04
Bacteria, Proteobacteria, Deltaproteobacteria, Desulfuromonadales	3.97			-
Bacteria, Proteobacteria, Betaproteobacteria, Burkholderiales, Alcaligenaceae, Candidimonas	6.47			-
Bacteria, Proteobacteria, Gammaproteobacteria, Cellvibrionales, Cellvibrionaceae	5.07			-
Bacteria, Bacteroidetes, Sphingobacteriia, Sphingobacteriales, Chitinophagaceae, Taibaiella	6.80			-
Bacteria, Actinobacteria, Actinobacteria, Micrococcales, Dermacoccaceae, Flexivirga	5.87			-
Bacteria, Actinobacteria, Actinobacteria, Micrococcales, Cellulomonadaceae, Actinotalea	4.26			-
Bacteria, Proteobacteria, Alphaproteobacteria, Rhizobiales, Rhizobiaceae, Rhizobium	5.98	CP	5.48	0.05
Bacteria, Proteobacteria, Alphaproteobacteria, Sphingomonadales	5.79			-
Bacteria, Chloroflexi, Chloroflexia	5.99	CP	5.61	0.05

Bacteria, Cyanobacteria, Cyanobacteria, SubsectionIII, FamilyI_o__SubsectionIII, Phormidium	0.00			-
Bacteria, Planctomycetes, Planctomycetacia, Brocadiales, Brocadiaceae, Candidatus_Kuenenia	4.12			-
Bacteria, Proteobacteria, Alphaproteobacteria, Sphingomonadales, Sphingomonadaceae, Novosphingobium	5.48	CP	5.03	0.05
Bacteria, Acidobacteria, Acidobacteria, Blastocatellales, Blastocatellaceae__Subgroup_4	6.09			-
Bacteria, Spirochaetae	4.84			-
Bacteria, Proteobacteria, Deltaproteobacteria, Myxococcales	5.95			-
Bacteria, Actinobacteria, Actinobacteria, Micrococcales, Dermacoccaceae	5.87			-
Bacteria, Proteobacteria, Gammaproteobacteria, Xanthomonadales, Xanthomonadales_Incertae_Sedis	6.43			-
Bacteria, Planctomycetes, BD7_11	4.42			-
Bacteria, Proteobacteria, Betaproteobacteria, Burkholderiales, Comamonadaceae, Ramlibacter	6.15			-
Bacteria, Chloroflexi, Ktedonobacteria, Ktedonobacterales, Thermosporotrichaceae	5.94	CP	5.45	0.05
Bacteria, Proteobacteria, Betaproteobacteria, Burkholderiales, Comamonadaceae, Comamonas	0.00			-
Bacteria, Actinobacteria, Actinobacteria, Gaiellales, Gaiellaceae, Gaiella	6.00			-
Bacteria, Proteobacteria, Gammaproteobacteria, Oceanospirillales	6.03			-
Bacteria, Firmicutes, Clostridia, Clostridiales	3.98			-
Bacteria, Verrucomicrobia, Verrucomicrobiae, Verrucomicrobiales, Verrucomicrobiaceae	4.52			-
Bacteria, Verrucomicrobia, Opitutae, Opitutales, Opitutaceae	5.85			-

Bacteria, Gemmatimonadetes, Gemmatimonadetes, Gemmatimonadales, Gemmatimonadaceae, Gemmatimonas	7.06			-
Bacteria, Elusimicrobia, Elusimicrobia, Lineage_IV	5.36	CK	4.99	0.05
Bacteria, Proteobacteria, Alphaproteobacteria, Rhizobiales, Phyllobacteriaceae, Aquamicrobium	5.42			-
Bacteria, Proteobacteria, Deltaproteobacteria, Bradymonadales, Bradymonadaceae	4.20			-
Bacteria, Actinobacteria, Actinobacteria, Micromonosporales, Micromonosporaceae, Luedemannella	5.13			-
Bacteria, Proteobacteria, Deltaproteobacteria, Myxococcales, Sandaracinaceae, Sandaracinus	4.61	CP	4.63	0.05
Bacteria, Planctomycetes, Planctomycetacia, Planctomycetales, Planctomycetaceae	6.00			-
Bacteria, Gemmatimonadetes, Gemmatimonadetes, Gemmatimonadales, Gemmatimonadaceae	7.01			-
Bacteria, Firmicutes, Bacilli, Lactobacillales, Streptococcaceae, Lactococcus	6.06			-
Bacteria, Chlorobi, Chlorobia, Chlorobiales, SJA_28	3.82			-
Bacteria, Actinobacteria, Actinobacteria, Solirubrobacterales, Solirubrobacteraceae, Solirubrobacter	3.93			-
Bacteria, Verrucomicrobia, Spartobacteria, Chthoniobacterales, DA101_soil_group	6.35	CK	5.77	0.05
Bacteria, Actinobacteria, Actinobacteria, Propionibacteriales, Nocardioidaceae, Kribbella	5.34			-
Bacteria, Bacteroidetes, Sphingobacteriia, Sphingobacteriales, PHOS_HE51	4.94	CP	4.79	0.04
Bacteria, Bacteroidetes, Sphingobacteriia, Sphingobacteriales, Chitinophagaceae, Sediminibacterium	5.27	CP	4.82	0.05
Bacteria, Proteobacteria	5.87			-
Bacteria, Proteobacteria, Betaproteobacteria, Burkholderiales, Alcaligenaceae, Castellaniella	6.52			-

Bacteria, Gemmatimonadetes, Gemmatimonadetes, Gemmatimonadales	7.35			-
Bacteria, Chloroflexi, Ktedonobacteria, Ktedonobacterales, 1921_3	5.81			-
Bacteria, Bacteroidetes, Cytophagia	6.54	CP	6.13	0.05
Bacteria, Bacteroidetes, Flavobacteriia, Flavobacteriales, Cryomorphaceae, Wandonia	6.02	CP	5.71	0.05
Bacteria, Proteobacteria, Alphaproteobacteria, Caulobacterales, Caulobacteraceae, Asticcacaulis	5.85	CP	5.42	0.05
Bacteria, Proteobacteria, Gammaproteobacteria, Xanthomonadales, Nevskiaceae, Nevskia	4.16			-
Bacteria, Proteobacteria, Gammaproteobacteria, Xanthomonadales, Solimonadaceae	5.81	CP	5.50	0.05
Bacteria, Deinococcus_Thermus, Deinococci, Deinococcales, Trueperaceae, Truepera	5.42			-
Bacteria, Proteobacteria, Alphaproteobacteria, Caulobacterales, Caulobacteraceae, Brevundimonas	5.66			-
Bacteria, Chloroflexi, Chloroflexia, Chloroflexales, Roseiflexaceae	5.97	CP	5.59	0.05
Bacteria, Bacteroidetes, Flavobacteriia, Flavobacteriales, Cryomorphaceae	0.00			-
Bacteria, Proteobacteria, Deltaproteobacteria, Myxococcales, Polyangiaceae, Sorangium	5.09			-
Bacteria, Acidobacteria, Acidobacteria, Holophagales, Holophagaceae, Holophaga	6.22			-
Bacteria, Chloroflexi, Ktedonobacteria, Ktedonobacterales, BacC_u_018	5.26			-
Bacteria, Actinobacteria, Actinobacteria, Micrococcales, Cellulomonadaceae	4.26			-
Bacteria, Spirochaetae, Spirochaetes, Spirochaetales	4.84			-
Bacteria, Proteobacteria, Alphaproteobacteria, Rhodospirillales	6.12			-

Bacteria, Verrucomicrobia, Verrucomicrobiae, Verrucomicrobiales, Verrucomicrobiaceae, Luteolibacter	3.68			-
Bacteria, Proteobacteria, Alphaproteobacteria, Rhodospirillales, Rhodospirillales_Incertae_Sedis, Reyranella	6.26			-
Bacteria, FBP	5.80	CK	5.28	0.05
Bacteria, Armatimonadetes, Fimbriimonadia, Fimbriimonadales, Fimbriimonadaceae	5.35			-
Bacteria, Chlamydiae, Chlamydiae, Chlamydiales, Parachlamydiaceae	4.25			-
Bacteria, Verrucomicrobia, Opitutae, Opitutales, Opitutaceae, Opitutus	5.85			-
Bacteria, Planctomycetes, Planctomycetacia, Planctomycetales	6.20			-
Bacteria, Proteobacteria, Gammaproteobacteria, Xanthomonadales, Xanthomonadaceae, Mizugakiibacter	7.65			-
Bacteria, Planctomycetes, Phycisphaerae, Phycisphaerales, Phycisphaeraceae, Phycisphaera	4.69			-
Bacteria, Actinobacteria, Actinobacteria, Propionibacteriales, Nocardioidaceae	6.64	CP	6.10	0.05
Bacteria, Proteobacteria, Betaproteobacteria, Burkholderiales, Oxalobacteraceae, Duganella	0.00			-
Bacteria, Verrucomicrobia, Opitutae, Opitutales	5.85			-
Bacteria, Parcubacteria, Candidatus_Campbellbacteria	0.00			-
Bacteria, Bacteroidetes, Flavobacteriia, Flavobacteriales	6.64	CP	6.20	0.05
Bacteria, Proteobacteria, Gammaproteobacteria, Methylococcales, Methylococcaceae, Methylocaldum	3.58			-
Bacteria, Proteobacteria, Alphaproteobacteria, Rhodobacterales	5.24	CP	4.81	0.05
Bacteria, Proteobacteria, Deltaproteobacteria, Myxococcales, Archangiaceae, Anaeromyxobacter	5.64			-

Bacteria, Proteobacteria, Gammaproteobacteria, Legionellales, Coxiellaceae, Aquicella	5.77			-
Bacteria, Firmicutes, Bacilli, Bacillales, Sporolactobacillaceae	6.41	CK	6.06	0.05
Bacteria, Proteobacteria, Gammaproteobacteria, Xanthomonadales, Xanthomonadaceae, Rudaea	4.26			-
Bacteria, Firmicutes, Bacilli, Bacillales	7.07			-
Bacteria, Proteobacteria, Deltaproteobacteria, Myxococcales, BIrii41	5.37	CP	5.07	0.04
Bacteria, Proteobacteria, Betaproteobacteria, Burkholderiales, Alcaligenaceae, Achromobacter	5.23			-
Bacteria, Firmicutes, Bacilli, Bacillales, Paenibacillaceae, Brevibacillus	6.35			-
Bacteria, Actinobacteria, Actinobacteria, Micrococcales, Promicromonosporaceae, Cellulosimicrobium	4.12			-
Bacteria, Chloroflexi, Ktedonobacteria, Thermogemmatisporales	5.40			-
Bacteria, Proteobacteria, Betaproteobacteria, Rhodocyclales, Rhodocyclaceae	5.16			-
Bacteria, Proteobacteria, Deltaproteobacteria, Myxococcales, Nannocystaceae	0.00			-
Bacteria, Acidobacteria, Acidobacteria, Blastocatellales, Blastocatellaceae__Subgroup_4, 11_24	5.34			-
Bacteria, Actinobacteria, Actinobacteria, Micrococcales, Promicromonosporaceae	4.12			-
Bacteria, Bacteroidetes, Sphingobacteriia, Sphingobacteriales, Chitinophagaceae, Parafilimonas	4.73			-
Bacteria, Bacteroidetes, Sphingobacteriia, Sphingobacteriales, Chitinophagaceae, Niastella	5.02			-
Bacteria, Actinobacteria, Actinobacteria, Solirubrobacterales, Patulibacteraceae	4.64			-
Bacteria, Chloroflexi, Chloroflexia, Chloroflexales	5.99	CP	5.63	0.05

Bacteria, Bacteroidetes, Flavobacteriia, Flavobacteriales, Flavobacteriaceae, Pricia	5.82	CP	5.42	0.05
Bacteria, Proteobacteria, Alphaproteobacteria, Rickettsiales	5.54			-
Bacteria, Chloroflexi, Ktedonobacteria, Thermogemmatisporales, 1921_2	5.40			-
Bacteria, Actinobacteria, Actinobacteria, Streptosporangiales, Streptosporangiaceae, Microbispora	5.96	CK	5.59	0.05
Bacteria, Cyanobacteria, Cyanobacteria, Vampirovibrionales	4.48			-
Bacteria, Verrucomicrobia, Verrucomicrobiae	4.52			-
Bacteria, Actinobacteria, Actinobacteria, Corynebacteriales, Nocardiaceae	5.63			-
Bacteria, Planctomycetes, Planctomycetacia	6.20			-
Bacteria, Bacteroidetes, Sphingobacteriia, Sphingobacteriales, Saprospiraceae	4.98			-
Bacteria, Proteobacteria, Alphaproteobacteria, Rhodobacterales, Rhodobacteraceae, Rhodobacter	4.43			-
Bacteria, Chloroflexi, TK10	6.21			-
Bacteria, Actinobacteria, Actinobacteria, Corynebacteriales, Mycobacteriaceae	6.10			-
Bacteria, Chlorobi	5.77			-
Bacteria, Proteobacteria, Gammaproteobacteria, Pseudomonadales, Pseudomonadaceae, Pseudomonas	6.22	CP	5.81	0.05
Bacteria, Bacteroidetes, Bacteroidia	5.09			-
Bacteria, Actinobacteria, Actinobacteria, Micrococcales, Microbacteriaceae	6.08			-
Bacteria, Gemmatimonadetes, Gemmatimonadetes, Gemmatimonadales, Gemmatimonadaceae, Gemmatirosa	5.69			-

Bacteria, Actinobacteria, Actinobacteria, Acidimicrobiales, Acidimicrobiales_Incertae_Sedis, Candidatus_Microthrix	0.00			-
*Bacteria, Proteobacteria, Deltaproteobacteria, SAR324_clade_Marine_group_B_*	4.30			-
Bacteria, Chloroflexi, Gitt_GS_136	5.43	CP	4.83	0.05
Bacteria, Acidobacteria, Acidobacteria, Blastocatellales, Blastocatellaceae__Subgroup_4, DS_100	0.00			-
Bacteria, Proteobacteria, Gammaproteobacteria, Enterobacteriales, Enterobacteriaceae, Kluyvera	5.45	CP	5.09	0.05
Bacteria, Proteobacteria, Betaproteobacteria, Neisseriales	4.25			-
Bacteria, Proteobacteria, Alphaproteobacteria, Rickettsiales, Rickettsiales_Incertae_Sedis, Candidatus_Odyssella	4.34			-
Bacteria, Deinococcus_Thermus, Deinococci, Deinococcales	5.43			-
Bacteria, Proteobacteria, Betaproteobacteria, Burkholderiales, Alcaligenaceae	6.41	CP	5.97	0.05
Bacteria, Bacteroidetes, Sphingobacteriia, Sphingobacteriales, KD3_93	4.71			-
Bacteria, Proteobacteria, Betaproteobacteria, Burkholderiales, Comamonadaceae, Xenophilus	4.96			-
Bacteria, Bacteroidetes, Sphingobacteriia, Sphingobacteriales, NS11_12_marine_group	5.05			-
Bacteria, Proteobacteria, Gammaproteobacteria, Xanthomonadales, Xanthomonadaceae, Pseudoxanthomonas	5.07	CP	4.81	0.05
Bacteria, Proteobacteria, Gammaproteobacteria, Xanthomonadales, Solimonadaceae, Polycyclovorans	5.43	CP	5.13	0.05
Bacteria, Spirochaetae, Spirochaetes, Spirochaetales, Spirochaetaceae, Spirochaeta_2	4.69			-
Bacteria, Nitrospirae	6.36			-
Bacteria, Proteobacteria, Betaproteobacteria, Methylophilales, Methylophilaceae, Methylobacillus	5.02	CP	4.73	0.05

Bacteria, Firmicutes, Bacilli, Bacillales, Alicyclobacillaceae	5.46			-
Bacteria, Verrucomicrobia, Spartobacteria, Chthoniobacterales, Xiphinematobacteraceae, Candidatus_Xiphinematobacter	5.01			-
Bacteria, Bacteroidetes, Sphingobacteriia, Sphingobacteriales, Sphingobacteriaceae, Parapedobacter	5.85			-
Bacteria, Fibrobacteres, Fibrobacteria, Fibrobacterales, Fibrobacteraceae, possible_genus_04	4.42			-
Bacteria, Cyanobacteria, Cyanobacteria, SubsectionIII, FamilyI_o__SubsectionIII, Microcoleus	4.12			-
Bacteria, Bacteroidetes, Cytophagia, Cytophagales, Cytophagaceae, Emticicia	4.16			-
Bacteria, Firmicutes, Clostridia, Clostridiales, Clostridiaceae_1, Clostridium_sensu_stricto_1	5.62			-
Bacteria, Firmicutes, Bacilli, Bacillales, Sporolactobacillaceae, Sporolactobacillus	6.32	CK	5.95	0.05
Bacteria, Firmicutes, Clostridia, Clostridiales, Clostridiaceae_1, Clostridium_sensu_stricto_9	4.67			-
Bacteria, Chloroflexi, Ktedonobacteria, Ktedonobacterales, JG30a_KF_32	6.19			-
Bacteria, Chlamydiae, Chlamydiae	5.05			-
Bacteria, Proteobacteria, Alphaproteobacteria, Rhodospirillales, Acetobacteraceae, Acidisoma	4.26			-
Bacteria, Proteobacteria, Alphaproteobacteria, Rhizobiales, Phyllobacteriaceae, Aminobacter	5.73			-
Bacteria, Bacteroidetes, Bacteroidia, Bacteroidales, Porphyromonadaceae, Dysgonomonas	0.00			-
Bacteria, Chloroflexi, Anaerolineae, Anaerolineales	6.32	CP	5.91	0.05
Bacteria, Bacteroidetes, Sphingobacteriia, Sphingobacteriales, Chitinophagaceae, Flavisolibacter	6.14			-
Bacteria, Chlamydiae, Chlamydiae, Chlamydiales, Simkaniaceae	3.93			-

Bacteria, Chloroflexi, Anaerolineae, Anaerolineales, Anaerolineaceae	6.32	CP	5.90	0.05
Bacteria, Actinobacteria, Actinobacteria, Solirubrobacterales, Elev_16S_1332	4.53			-
Bacteria, Bacteroidetes, Sphingobacteriia, Sphingobacteriales, Chitinophagaceae, Ferruginibacter	5.27	CP	4.95	0.05
Bacteria, Chloroflexi	5.33			-
Bacteria, Acidobacteria, Acidobacteria, Holophagales	6.52	CP	6.05	0.05
Bacteria, Actinobacteria, Actinobacteria, Solirubrobacterales, 0319_6M6	4.93			-
Bacteria, Proteobacteria, Betaproteobacteria, Hydrogenophilales	4.86			-
Bacteria, Proteobacteria, Alphaproteobacteria, Rhizobiales, Rhodobiaceae	5.50			-
Bacteria, Proteobacteria, Alphaproteobacteria, Sphingomonadales, Sphingomonadaceae, Rhizorhapis	4.67			-
Bacteria, Bacteroidetes, Flavobacteriia, Flavobacteriales, Flavobacteriaceae, Empedobacter	4.38			-
Bacteria, Firmicutes, Bacilli, Bacillales, Family_XII_o__Bacillales	4.76			-
Bacteria, Proteobacteria, Alphaproteobacteria, Caulobacterales, Hyphomonadaceae	4.16			-
Bacteria, Chloroflexi, JG37_AG_4	6.86			-
Bacteria, Firmicutes, Clostridia	5.03			-
Bacteria, Chloroflexi, SHA_26	4.63			-
Bacteria, Chloroflexi, Caldilineae	4.52			-
Bacteria, Bacteroidetes, Sphingobacteriia, Sphingobacteriales, Chitinophagaceae, Heliimonas	4.16			-

Bacteria, Planctomycetes, Planctomycetacia, Brocadiales, Brocadiaceae	4.12			-
Bacteria, Omnitrophica	3.58			-
Bacteria, Proteobacteria, Betaproteobacteria, Burkholderiales, Oxalobacteraceae, Paucimonas	4.41			-
Bacteria, Chloroflexi, Ktedonobacteria, C0119	6.27			-
Bacteria, Actinobacteria, Actinobacteria, Acidimicrobiales, Iamiaceae	5.28			-
Bacteria, Actinobacteria, Actinobacteria, Frankiales, Acidothermaceae, Acidothermus	6.18			-
Bacteria, Chloroflexi, Ktedonobacteria, JG30_KF_AS9	6.99			-
Bacteria, Firmicutes, Bacilli, Bacillales, Planococcaceae, Sporosarcina	5.30			-
Bacteria, Saccharibacteria	7.42			-
Bacteria, Proteobacteria, Gammaproteobacteria, Legionellales, Legionellaceae, Legionella	5.00			-
Bacteria, Bacteroidetes, Cytophagia, Cytophagales, Cyclobacteriaceae	0.00			-
Bacteria, Proteobacteria, Gammaproteobacteria, Cellvibrionales, Porticoccaceae, C1_B045	4.26			-
Bacteria, Actinobacteria, Actinobacteria, Micrococcales, Micrococcaceae, Sinomonas	4.49	CP	4.76	0.05
Bacteria, Proteobacteria, Alphaproteobacteria, Rickettsiales, RB446	4.16			-
Bacteria, Proteobacteria, Gammaproteobacteria, Aeromonadales	4.62	CP	4.58	0.04
Bacteria, Proteobacteria, Deltaproteobacteria, Desulfuromonadales, Geobacteraceae	3.97			-
Bacteria, Firmicutes, Bacilli, Lactobacillales, Enterococcaceae, Enterococcus	6.03			-

Bacteria, Firmicutes, Bacilli, Bacillales, Paenibacillaceae, Cohnella	6.32			-
Bacteria, Proteobacteria, Gammaproteobacteria, Methylococcales, Methylococcaceae	3.58			-
Bacteria, Deinococcus_Thermus, Deinococci, Deinococcales, Trueperaceae	5.42			-
Bacteria, Firmicutes, Bacilli, Bacillales, Paenibacillaceae	6.84			-
Bacteria, Proteobacteria, Gammaproteobacteria, Xanthomonadales, Nevskiaceae	6.21			-
Bacteria, Firmicutes, Clostridia, Halanaerobiales, ODP1230B8_23	6.62			-
Bacteria, Firmicutes, Clostridia, Clostridiales, Christensenellaceae, Christensenellaceae_R_7_group	3.58			-
Bacteria, Proteobacteria, Alphaproteobacteria, Rhizobiales, Rhizobiales_Incertae_Sedis, Bauldia	5.13			-
Bacteria, Proteobacteria, Deltaproteobacteria, Bdellovibrionales, Bdellovibrionaceae, Bdellovibrio	5.76			-
Bacteria, Verrucomicrobia, Spartobacteria, Chthoniobacterales, Xiphinematobacteraceae	5.01			-
Bacteria, Proteobacteria, Gammaproteobacteria, Oceanospirillales, Alcanivoracaceae, Alcanivorax	5.92			-
Bacteria, Proteobacteria, Betaproteobacteria, SC_I_84	6.74			-
Bacteria, Actinobacteria, Actinobacteria, Micromonosporales, Micromonosporaceae	5.31			-
Bacteria, Cyanobacteria, Cyanobacteria, SubsectionIII	5.11	CP	4.79	0.05
Bacteria, Actinobacteria, Actinobacteria, Propionibacteriales, Nocardioidaceae, Nocardioides	6.14	CP	5.61	0.05
Bacteria, Actinobacteria, Actinobacteria, Streptosporangiales, Thermomonosporaceae	5.57			-
Bacteria, Proteobacteria, Deltaproteobacteria, Desulfurellales, Desulfurellaceae, H16	6.06			-

Bacteria, Acidobacteria, Acidobacteria, Acidobacteriales, Acidobacteriaceae__Subgroup_1, Granulicella	5.99			-
Bacteria, Acidobacteria, Acidobacteria, Solibacterales, Solibacteraceae__Subgroup_3_, PAUC26f	4.63			-
Bacteria, Proteobacteria, Gammaproteobacteria, Thiotrichales, Thiotrichaceae	0.00			-
Bacteria, Actinobacteria, Actinobacteria, Catenulisporales, Actinospicaceae	5.62			-
Bacteria, Firmicutes, Limnochordia, Limnochordales	4.70	CK	4.60	0.04
Bacteria, Actinobacteria, Actinobacteria, Pseudonocardiales, Pseudonocardiaceae, Pseudonocardia	4.98			-
Bacteria, Verrucomicrobia, Verrucomicrobiae, Verrucomicrobiales	4.52			-
Bacteria, Chlorobi, Chlorobia, Chlorobiales	5.77			-
Bacteria, Proteobacteria, Alphaproteobacteria, Rhodospirillales, KCM_B_15	4.78			-
Bacteria, Proteobacteria, Gammaproteobacteria, Aeromonadales, Aeromonadaceae	4.62	CP	4.60	0.04
Bacteria, Actinobacteria, Actinobacteria, Pseudonocardiales	5.67			-
Bacteria, Proteobacteria, Betaproteobacteria, Burkholderiales, Oxalobacteraceae, Massilia	6.12			-
Bacteria, Bacteroidetes, Sphingobacteriia, Sphingobacteriales	5.33			-
Bacteria, Chloroflexi, Ktedonobacteria, Ktedonobacterales, FCPS473	5.35			-
Bacteria, Planctomycetes, Phycisphaerae, Phycisphaerales, Phycisphaeraceae	0.00			-
Bacteria, Proteobacteria, Alphaproteobacteria, Rhizobiales, Methylobacteriaceae, Microvirga	4.85			-
Bacteria, Proteobacteria, Alphaproteobacteria, Sphingomonadales, Sphingomonadaceae, Sphingobium	5.56			-

Bacteria, Firmicutes, Clostridia, Clostridiales, Family_XVII, Sulfobacillus	4.38			-
Bacteria, Proteobacteria, Gammaproteobacteria, Oceanospirillales, Alcanivoracaceae	5.92			-
Bacteria, Nitrospirae, Nitrospira, Nitrospira	6.36			-
Bacteria, Proteobacteria, Alphaproteobacteria, Rhizobiales, Xanthobacteraceae, Variibacter	5.48			-
Bacteria, Gemmatimonadetes, Gemmatimonadetes	4.66			-
Bacteria, Proteobacteria, Alphaproteobacteria, Rickettsiales, Rickettsiales_Incertae_Sedis	4.34			-
Bacteria, Bacteroidetes, Sphingobacteriia, Sphingobacteriales, CWT_CU03_E12	5.24			-
Bacteria, Chloroflexi, Ktedonobacteria	5.33			-
Bacteria, Proteobacteria, Alphaproteobacteria, Rhodospirillales, Acetobacteraceae, Acidiphilium	4.85			-
Bacteria, Proteobacteria, Alphaproteobacteria, Rhodospirillales, I_10	5.27			-
Bacteria, Chloroflexi, Caldilineae, Caldilineales	4.52			-
Bacteria, Proteobacteria, Deltaproteobacteria, Desulfurellales, Desulfurellaceae	6.06			-
Bacteria, Proteobacteria, Alphaproteobacteria, Rickettsiales, Holosporaceae	4.52			-
Bacteria, Actinobacteria, Actinobacteria, Pseudonocardiales, Pseudonocardiaceae, Lechevalieria	4.91	CP	4.66	0.05
Bacteria, Verrucomicrobia, Verrucomicrobiae, Verrucomicrobiales, Verrucomicrobiaceae, Haloferula	4.52			-
Bacteria, Bacteroidetes, Sphingobacteriia	7.63			-
Bacteria, Proteobacteria, Betaproteobacteria, Burkholderiales, Burkholderiaceae, Ralstonia	5.86			-

Bacteria, Actinobacteria, Actinobacteria, Corynebacteriales, Mycobacteriaceae, Mycobacterium	6.10			-
Bacteria, Chloroflexi, Anaerolineae	6.32	CP	5.89	0.05
Bacteria, Bacteroidetes, Bacteroidia, Bacteroidales, Porphyromonadaceae	5.09			-
Bacteria, Actinobacteria	7.56			-
Bacteria, Proteobacteria, Alphaproteobacteria, Alphaproteobacteria_Incertae_Sedis	4.76			-
Bacteria, Actinobacteria, Actinobacteria, Acidimicrobiales, Acidimicrobiaceae, CL500_29_marine_group	4.86			-
Bacteria, Acidobacteria, Acidobacteria, Subgroup_10	5.71	CP	5.24	0.05
Bacteria, Cyanobacteria, Cyanobacteria	6.01			-
Bacteria, Acidobacteria, Acidobacteria, Subgroup_7	6.01			-
Bacteria	6.00			-
**Taxonomy**	**Mean**	**Group**	**LDA**	***P* value**
Bacteria, Proteobacteria, Betaproteobacteria, Burkholderiales, Oxalobacteraceae, Pseudoduganella	4.92			-
Bacteria, Actinobacteria, Actinobacteria, Streptosporangiales, Streptosporangiaceae, Streptosporangium	3.58			-
Bacteria, Firmicutes, Bacilli, Lactobacillales, Streptococcaceae	6.04			-
Bacteria, Proteobacteria, Deltaproteobacteria, Myxococcales, Archangiaceae	4.66			-
Bacteria, Proteobacteria, Alphaproteobacteria, Rhizobiales, Brucellaceae, Ochrobactrum	4.84			-
Bacteria, Actinobacteria, Actinobacteria, Solirubrobacterales, YNPFFP1	5.55			-

Bacteria, Proteobacteria, Gammaproteobacteria, Xanthomonadales, Xanthomonadaceae	5.76			-
Bacteria, Proteobacteria, Alphaproteobacteria, Sphingomonadales, Erythrobacteraceae, Altererythrobacter	5.59			-
Bacteria, Proteobacteria, Gammaproteobacteria, Oceanospirillales, Hahellaceae, Hahella	3.68			-
Bacteria, Firmicutes, Clostridia, Clostridiales, Peptostreptococcaceae, Paeniclostridium	5.43			-
Bacteria, Actinobacteria, Actinobacteria, Kineosporiales	5.69	CK	5.20	0.05
Bacteria, Proteobacteria, Betaproteobacteria, Burkholderiales	7.07			-
Bacteria, Bacteroidetes, Sphingobacteriia, Sphingobacteriales, Chitinophagaceae, Lacibacter	3.86			-
Bacteria, Actinobacteria, Actinobacteria, Acidimicrobiales, Acidimicrobiaceae	4.64			-
Bacteria, Proteobacteria, Gammaproteobacteria, Xanthomonadales, Xanthomonadaceae, Dokdonella	5.67			-
Bacteria, Proteobacteria, Alphaproteobacteria, Sphingomonadales, Sphingomonadaceae, Zymomonas	4.13			-
Bacteria, Chlorobi, Chlorobia, Chlorobiales, OPB56	5.73			-
Bacteria, Proteobacteria, Alphaproteobacteria, Rhizobiales, Phyllobacteriaceae	6.57			-
Bacteria, Bacteroidetes, Sphingobacteriia, Sphingobacteriales, Sphingobacteriaceae, Nubsella	3.56			-
Bacteria, Actinobacteria, Actinobacteria, Pseudonocardiales, Pseudonocardiaceae, Kutzneria	4.58			-
Bacteria, Elusimicrobia, Elusimicrobia, Lineage_IIc	4.34			-
Bacteria, Elusimicrobia, Elusimicrobia, Lineage_IIb	4.73			-
Bacteria, Elusimicrobia, Elusimicrobia, Lineage_IIa	4.16			-

Bacteria, Proteobacteria, Alphaproteobacteria, Rhizobiales, Hyphomicrobiaceae, Rhodoplanes	5.46			-
Bacteria, Bacteroidetes	5.21			-
Bacteria, Actinobacteria, Actinobacteria, Corynebacteriales, Nocardiaceae, Rhodococcus	5.04			-
Bacteria, Gemmatimonadetes, Gemmatimonadetes, Longimicrobiales	5.38			-
Bacteria, Proteobacteria, Alphaproteobacteria, Rhizobiales, Xanthobacteraceae, Pseudolabrys	6.55			-
Bacteria, Verrucomicrobia, OPB35_soil_group, Pedosphaera	4.41			-
Bacteria, Proteobacteria, Gammaproteobacteria, Pseudomonadales, Moraxellaceae	4.77			-
Bacteria, Acidobacteria, Acidobacteria, Subgroup_10, ABS_19	5.25			-
Bacteria, Bacteroidetes, Flavobacteriia, Flavobacteriales, Flavobacteriaceae	6.63			-
Bacteria, Ignavibacteriae, Ignavibacteria, Ignavibacteriales	4.26			-
Bacteria, Actinobacteria, Actinobacteria, Propionibacteriales	6.48			-
Bacteria, Proteobacteria, Deltaproteobacteria, Bdellovibrionales, Bdellovibrionaceae, OM27_clade	4.16			-
Bacteria, Bacteroidetes, Cytophagia, Cytophagales, Cytophagaceae, Dyadobacter	4.98	LM	4.81	0.05
Bacteria, Proteobacteria, Alphaproteobacteria, Sphingomonadales, Sphingomonadaceae, Sphingopyxis	5.64			-
Bacteria, Deinococcus_Thermus, Deinococci	5.77			-
Bacteria, Proteobacteria, Gammaproteobacteria, Legionellales, Legionellaceae	4.64			-
Bacteria, Proteobacteria, Gammaproteobacteria, Thiotrichales, EV818SWSAP88	3.93			-

Bacteria, Bacteroidetes, Sphingobacteriia, Sphingobacteriales, Sphingobacteriaceae, Sphingobacterium	5.14			-
Bacteria, Verrucomicrobia, Opitutae	5.74			-
Bacteria, Proteobacteria, Deltaproteobacteria, Bdellovibrionales	6.03			-
Bacteria, Chloroflexi, Ktedonobacteria, Ktedonobacterales, Ktedonobacteraceae	5.03			-
Bacteria, Bacteroidetes, Bacteroidia, Bacteroidales, Porphyromonadaceae, Petrimonas	5.09			-
Bacteria, Firmicutes, Bacilli, Lactobacillales, Carnobacteriaceae	4.41			-
Bacteria, Proteobacteria, Gammaproteobacteria, Enterobacteriales	5.55			-
Bacteria, Candidatus_Berkelbacteria	4.26			-
Bacteria, Bacteroidetes, Sphingobacteriia, Sphingobacteriales, Chitinophagaceae, Dinghuibacter	3.91			-
Bacteria, Proteobacteria, Alphaproteobacteria, Rhizobiales, Rhizobiales_Incertae_Sedis	6.82			-
Bacteria, Proteobacteria, Gammaproteobacteria, Pseudomonadales, Moraxellaceae, Acinetobacter	4.51			-
Bacteria, Proteobacteria, Deltaproteobacteria, Myxococcales, P3OB_42	5.72			-
Bacteria, Proteobacteria, Alphaproteobacteria, Rhizobiales, MNG7	4.33			-
Bacteria, Actinobacteria, Actinobacteria, Gaiellales, Gaiellaceae	6.00			-
Bacteria, Proteobacteria, Alphaproteobacteria, Rhizobiales, Hyphomicrobiaceae	6.60			-
Bacteria, Actinobacteria, Actinobacteria, Catenulisporales, Catenulisporaceae, Catenulispora	5.12			-
Bacteria, Firmicutes, Erysipelotrichia	5.37			-

Bacteria, Proteobacteria, Alphaproteobacteria, Rhizobiales	5.68			-
Bacteria, Verrucomicrobia, OPB35_soil_group	6.29			-
Bacteria, Planctomycetes, Phycisphaerae	6.11	CK	5.59	0.05
Bacteria, Firmicutes, Bacilli, Bacillales, Family_XII_o__Bacillales, Exiguobacterium	4.76			-
Bacteria, Proteobacteria, Alphaproteobacteria, Rhodospirillales, Acetobacteraceae	6.19			-
Bacteria, Bacteroidetes, Flavobacteriia, Flavobacteriales, Flavobacteriaceae, Arenibacter	5.29			-
Bacteria, Armatimonadetes, Chthonomonadetes, Chthonomonadales, Chthonomonadaceae	4.28			-
Bacteria, Proteobacteria, Betaproteobacteria, Burkholderiales, Comamonadaceae, Aquincola	5.32			-
Bacteria, Firmicutes, Bacilli, Lactobacillales	6.34			-
Bacteria, Actinobacteria, Actinobacteria, Micrococcales, Bogoriellaceae	4.19			-
Bacteria, Proteobacteria, Betaproteobacteria, Burkholderiales, Oxalobacteraceae	5.75			-
Bacteria, Actinobacteria, Actinobacteria, Catenulisporales, Catenulisporaceae	5.12			-
Bacteria, Verrucomicrobia, Spartobacteria	6.39	CK	5.81	0.05
Bacteria, Chlorobi, Chlorobia	5.73			-
Bacteria, Proteobacteria, Gammaproteobacteria, Thiotrichales	3.93			-
Bacteria, Verrucomicrobia, Spartobacteria, Chthoniobacterales	3.58			-
Bacteria, Proteobacteria, Gammaproteobacteria, Xanthomonadales, Nevskiaceae, Alkanibacter	6.41			-

Bacteria, Deinococcus_Thermus, Deinococci, Deinococcales, Deinococcaceae, Deinococcus	4.23	LM	4.87	0.04
Bacteria, Proteobacteria, Deltaproteobacteria, Myxococcales, Sandaracinaceae	4.78			-
Bacteria, Bacteroidetes, Sphingobacteriia, Sphingobacteriales, Chitinophagaceae, Segetibacter	4.46			-
Bacteria, Proteobacteria, Betaproteobacteria, Burkholderiales, Burkholderiaceae	3.56			-
Bacteria, Actinobacteria, Actinobacteria, Gaiellales	6.78			-
Bacteria, Proteobacteria, Gammaproteobacteria, Cellvibrionales, Cellvibrionaceae, Cellvibrio	5.11			-
Bacteria, Bacteroidetes, Flavobacteriia, Flavobacteriales, Flavobacteriaceae, Muricauda	4.40	LM	4.67	0.04
Bacteria, Acidobacteria, Acidobacteria, Solibacterales	6.83			-
Bacteria, Bacteroidetes, Sphingobacteriia, Sphingobacteriales, AKYH767	4.70			-
Bacteria, Acidobacteria, Acidobacteria	6.86			-
Bacteria, Firmicutes, Erysipelotrichia, Erysipelotrichales, Erysipelotrichaceae, Turicibacter	5.37			-
Bacteria, Proteobacteria, Gammaproteobacteria, Cellvibrionales, Porticoccaceae	4.26			-
Bacteria, Proteobacteria, Alphaproteobacteria, Rickettsiales, Rickettsiaceae, Candidatus_Trichorickettsia	0.00			-
Bacteria, Proteobacteria, Alphaproteobacteria, Rhodobacterales, Rhodobacteraceae, Rubellimicrobium	4.78			-
Bacteria, Proteobacteria, Alphaproteobacteria, Rhizobiales, Rhizobiales_Incertae_Sedis, Rhizomicrobium	6.78			-
Bacteria, Deinococcus_Thermus	5.77			-
Bacteria, Proteobacteria, Alphaproteobacteria, Rhizobiales, Methylobacteriaceae, Methylobacterium	4.44			-

Bacteria, Proteobacteria, Alphaproteobacteria, Rhizobiales, Hyphomicrobiaceae, Devosia	6.48			-
Bacteria, Firmicutes, Erysipelotrichia, Erysipelotrichales, Erysipelotrichaceae	5.37			-
Bacteria, Proteobacteria, Alphaproteobacteria, Rhodobacterales, Rhodobacteraceae, Paracoccus	4.74			-
Bacteria, Chloroflexi, Ktedonobacteria, Ktedonobacterales, Thermosporotrichaceae, Thermosporothrix	4.72			-
Bacteria, Proteobacteria, Gammaproteobacteria, Xanthomonadales, Xanthomonadales_Incertae_Sedis, Steroidobacter	5.69			-
Bacteria, Proteobacteria, Alphaproteobacteria, Rickettsiales, Mitochondria	3.72			-
Bacteria, Proteobacteria, Alphaproteobacteria, Sphingomonadales, Sphingomonadaceae, Sphingomonas	7.34			-
Bacteria, Actinobacteria, Actinobacteria, Streptomycetales, Streptomycetaceae, Streptomyces	5.99			-
Bacteria, Proteobacteria, Gammaproteobacteria, Pseudomonadales, Pseudomonadaceae	5.90	LM	5.40	0.05
Bacteria, Chloroflexi, Ktedonobacteria, Ktedonobacterales, 1959_1	4.47			-
Bacteria, Planctomycetes, Phycisphaerae, Tepidisphaerales	6.10	CK	5.59	0.05
Bacteria, Proteobacteria, Alphaproteobacteria, Alphaproteobacteria_Incertae_Sedis, Micavibrio	4.45	LM	4.73	0.04
Bacteria, Proteobacteria, Gammaproteobacteria, Xanthomonadales, Xanthomonadaceae, Panacagrimonas	4.61			-
Bacteria, Proteobacteria, Deltaproteobacteria, Myxococcales, mle1_27	4.60	LM	4.94	0.04
Bacteria, Actinobacteria, Actinobacteria, Frankiales, Geodermatophilaceae	5.65			-
Bacteria, Acidobacteria, Acidobacteria, Solibacterales, Solibacteraceae__Subgroup_3_, AKIW659	4.51			-
Bacteria, Chloroflexi, Thermomicrobia, Sphaerobacterales	5.86	CK	5.31	0.05

Bacteria, Proteobacteria, Gammaproteobacteria, Xanthomonadales, Xanthomonadaceae, Lysobacter	5.91			-
Bacteria, Proteobacteria, Betaproteobacteria, Neisseriales, Neisseriaceae	4.19			-
Bacteria, Proteobacteria, Gammaproteobacteria, Methylococcales	4.04			-
Bacteria, Proteobacteria, Alphaproteobacteria, Sphingomonadales, Erythrobacteraceae	6.17			-
Bacteria, Peregrinibacteria, Candidatus_Peribacteria	4.63	LM	4.57	0.05
Bacteria, Proteobacteria, Gammaproteobacteria, Xanthomonadales, Xanthomonadaceae, Luteimonas	6.08			-
Bacteria, Proteobacteria, Alphaproteobacteria, Rhizobiales, Rhodobiaceae, Parvibaculum	5.71			-
Bacteria, Actinobacteria, Actinobacteria, Micrococcales, Demequinaceae, Lysinimicrobium	4.43			-
Bacteria, Bacteroidetes, Sphingobacteriia, Sphingobacteriales, Chitinophagaceae, Crenotalea	6.43			-
Bacteria, Proteobacteria, Gammaproteobacteria, Xanthomonadales, Xanthomonadaceae, Arenimonas	6.00			-
Bacteria, Firmicutes, Clostridia, Clostridiales, Clostridiaceae_1	4.54			-
Bacteria, Chloroflexi, P2_11E	4.67			-
Bacteria, Planctomycetes, Phycisphaerae, Phycisphaerales	4.28			-
Bacteria, Firmicutes, Bacilli, Bacillales, Bacillaceae, Oceanobacillus	4.64			-
Bacteria, Bacteroidetes, Sphingobacteriia, Sphingobacteriales, Sphingobacteriaceae, Mucilaginibacter	5.98			-
Bacteria, Bacteroidetes, Flavobacteriia, Flavobacteriales, Flavobacteriaceae, Confluentibacter	4.70			-
Bacteria, Bacteroidetes, Flavobacteriia, Flavobacteriales, Cryomorphaceae, Fluviicola	5.44			-

Bacteria, Proteobacteria, Betaproteobacteria, Burkholderiales, Comamonadaceae, Delftia	3.68			-
Bacteria, Firmicutes, Bacilli, Lactobacillales, Streptococcaceae, Streptococcus	4.66			-
Bacteria, Chlamydiae, Chlamydiae, Chlamydiales	5.05	CK	4.69	0.05
Bacteria, Proteobacteria, Gammaproteobacteria, Xanthomonadales, Xanthomonadaceae, Luteibacter	5.78	LM	5.52	0.05
Bacteria, WS6	3.56			-
Bacteria, Proteobacteria, Betaproteobacteria, Burkholderiales, Alcaligenaceae, Pusillimonas	5.29			-
Bacteria, Actinobacteria, Actinobacteria, Kineosporiales, Kineosporiaceae, Angustibacter	5.69	CK	5.18	0.05
Bacteria, Bacteroidetes, Flavobacteriia, Flavobacteriales, NS9_marine_group	5.32	LM	5.07	0.04
Bacteria, Actinobacteria, Actinobacteria, Streptomycetales	6.38			-
Bacteria, Bacteroidetes, Sphingobacteriia, Sphingobacteriales, Chitinophagaceae, Arachidicoccus	6.88			-
Bacteria, Bacteroidetes, Flavobacteriia, Flavobacteriales, Flavobacteriaceae, Vitellibacter	4.51			-
Bacteria, Proteobacteria, Betaproteobacteria, Burkholderiales, Burkholderiaceae, Pandoraea	5.17			-
Bacteria, Proteobacteria, Betaproteobacteria, Burkholderiales, Comamonadaceae, Aquabacterium	5.57			-
Bacteria, Proteobacteria, Betaproteobacteria, Burkholderiales, Comamonadaceae, Variovorax	4.52			-
Bacteria, Verrucomicrobia, Spartobacteria, Chthoniobacterales, Chthoniobacteraceae	4.98			-
Bacteria, Firmicutes, Bacilli, Bacillales, Bacillaceae, Bacillus	6.10			-
Bacteria, Proteobacteria, Betaproteobacteria, Rhodocyclales, Rhodocyclaceae, Uliginosibacterium	4.21			-

Bacteria, Bacteroidetes, Sphingobacteriia, Sphingobacteriales, Chitinophagaceae	7.09			-
Bacteria, Chloroflexi, Ktedonobacteria, Ktedonobacterales, G12_WMSP1	5.21			-
Bacteria, Proteobacteria, Alphaproteobacteria, Caulobacterales, Hyphomonadaceae, Hirschia	4.72			-
Bacteria, Bacteroidetes, Cytophagia, Cytophagales, Cytophagaceae, Sporocytophaga	5.23			-
Bacteria, Proteobacteria, Gammaproteobacteria, Pseudomonadales	5.93	LM	5.47	0.05
Bacteria, Actinobacteria, Actinobacteria, Streptosporangiales, Streptosporangiaceae	6.02			-
Bacteria, Planctomycetes, Planctomycetacia, Planctomycetales, Planctomycetaceae, Planctomyces	3.58			-
Bacteria, Proteobacteria, Alphaproteobacteria, Rhodospirillales, DA111	6.49			-
Bacteria, Actinobacteria, Actinobacteria, Pseudonocardiales, Pseudonocardiaceae, Saccharothrix	3.91			-
Bacteria, Proteobacteria, Alphaproteobacteria, Rhizobiales, Xanthobacteraceae	6.24			-
Bacteria, Bacteroidetes, Flavobacteriia, Flavobacteriales, Flavobacteriaceae, Galbibacter	3.95			-
Bacteria, Proteobacteria, Gammaproteobacteria, Xanthomonadales, Xanthomonadales_Incertae_Sedis, Acidibacter	6.33			-
Bacteria, Chloroflexi, Anaerolineae, Anaerolineales, Anaerolineaceae, Anaerolinea	4.30			-
Bacteria, Actinobacteria, Actinobacteria, Micromonosporales, Micromonosporaceae, Micromonospora	5.57			-
Bacteria, Proteobacteria, Alphaproteobacteria, Rickettsiales, Rickettsiaceae	0.00			-
Bacteria, Firmicutes, Clostridia, Clostridiales, Christensenellaceae	3.58			-
Bacteria, Proteobacteria, Alphaproteobacteria, Rhodospirillales, AKYH478	4.27			-

Bacteria, Acidobacteria, Acidobacteria, Vicinamibacter	4.51			-
Bacteria, Actinobacteria, Actinobacteria, Catenulisporales, Actinospicaceae, Actinospica	5.39	CK	4.99	0.05
Bacteria, Ignavibacteriae, Ignavibacteria, Ignavibacteriales, BSV26	4.69			-
Bacteria, Elusimicrobia, Elusimicrobia	5.46			-
Bacteria, Proteobacteria, Betaproteobacteria, Burkholderiales, Comamonadaceae	5.80			-
Bacteria, Proteobacteria, Gammaproteobacteria, Enterobacteriales, Enterobacteriaceae, _Serratia__fonticola_RB_25_group	0.00			-
Bacteria, Bacteroidetes, Cytophagia, Cytophagales, Cytophagaceae, Ohtaekwangia	4.37			-
Bacteria, Proteobacteria, Gammaproteobacteria, Oceanospirillales, Oceanospirillaceae, Pseudohongiella	5.33			-
Bacteria, Actinobacteria, Actinobacteria, Streptosporangiales	6.15			-
Bacteria, Acidobacteria, Acidobacteria, Acidobacteriales, Acidobacteriaceae__Subgroup_1, Edaphobacter	5.70			-
Bacteria, Acidobacteria, Acidobacteria, Holophagales, Holophagaceae	6.17	LM	5.82	0.05
Bacteria, Firmicutes, Bacilli, Bacillales, Bacillaceae	6.12			-
Bacteria, Actinobacteria, Actinobacteria, Frankiales, Sporichthyaceae	4.72	CK	4.68	0.05
Bacteria, Firmicutes, Bacilli, Bacillales, Planococcaceae	5.83			-
Bacteria, Firmicutes, Bacilli, Bacillales, Planococcaceae, Solibacillus	5.31			-
Bacteria, Proteobacteria, Alphaproteobacteria, Rhodospirillales, Acetobacteraceae, Rhodovastum	5.90			-
Bacteria, Proteobacteria, Betaproteobacteria, Hydrogenophilales, Hydrogenophilaceae, Thiobacillus	4.75			-

Bacteria, Firmicutes, Bacilli, Bacillales, Sporolactobacillaceae, Tuberibacillus	5.69	CK	5.37	0.04
Bacteria, Actinobacteria, Actinobacteria, Micrococcales, Intrasporangiaceae, Terrabacter	5.48	LM	4.74	0.05
Bacteria, Chloroflexi, Elev_1554	3.88			-
Bacteria, Proteobacteria, Gammaproteobacteria, Oceanospirillales, Halomonadaceae	4.04			-
Bacteria, Actinobacteria, Actinobacteria, Micrococcales, Micrococcaceae	6.20			-
Bacteria, Chloroflexi, Thermomicrobia	6.31	CK	5.68	0.05
Bacteria, Proteobacteria, Deltaproteobacteria, Desulfurellales	6.06			-
Bacteria, Spirochaetae, Spirochaetes	5.22			-
Bacteria, Chloroflexi, Thermomicrobia, AKYG1722	5.49			-
Bacteria, Bacteroidetes, Sphingobacteriia, Sphingobacteriales, Chitinophagaceae, Flavitalea	5.35			-
Bacteria, Actinobacteria, Actinobacteria, Frankiales, Frankiaceae, Jatrophihabitans	6.14			-
Bacteria, Proteobacteria, Gammaproteobacteria, Pseudomonadales, Moraxellaceae, Psychrobacter	0.00			-
Bacteria, Planctomycetes, Planctomycetacia, Planctomycetales, Planctomycetaceae, Singulisphaera	5.65			-
Bacteria, Firmicutes, Bacilli, Bacillales, Thermoactinomycetaceae, Shimazuella	4.95			-
Bacteria, Actinobacteria, Actinobacteria, Micrococcales, Microbacteriaceae, Lysinimonas	6.20	CK	5.42	0.05
Bacteria, Firmicutes, Bacilli, C178B	6.18	CK	5.84	0.05
Bacteria, Firmicutes, Clostridia, Clostridiales, Peptostreptococcaceae	5.30			-

Bacteria, Proteobacteria, Betaproteobacteria, Burkholderiales, Burkholderiaceae, Cupriavidus	4.57			-
Bacteria, Proteobacteria, Gammaproteobacteria, Xanthomonadales, Xanthomonadaceae, Dyella	6.73			-
Bacteria, Verrucomicrobia	6.63			-
Bacteria, Actinobacteria, Actinobacteria, Solirubrobacterales, Gsoil_1167	4.23	LM	4.86	0.04
Bacteria, Proteobacteria, Betaproteobacteria, Burkholderiales, Burkholderiaceae, Burkholderia_Paraburkholderia	6.30			-
Bacteria, Acidobacteria, Acidobacteria, Solibacterales, Solibacteraceae__Subgroup_3_, Candidatus_Solibacter	6.40			-
Bacteria, Proteobacteria, Deltaproteobacteria, Myxococcales, Phaselicystidaceae	4.30			-
Bacteria, Actinobacteria, Actinobacteria, Frankiales, Nakamurellaceae	5.61			-
Bacteria, Actinobacteria, Actinobacteria, Catenulisporales	5.53			-
Bacteria, Proteobacteria, Alphaproteobacteria, Rhodospirillales, Rhodospirillales_Incertae_Sedis	6.18			-
Bacteria, Firmicutes, Clostridia, Clostridiales, Lachnospiraceae, Lachnospiraceae_NK3A20_group	3.56			-
Bacteria, Planctomycetes, OM190	3.68			-
Bacteria, Proteobacteria, Betaproteobacteria, TRA3_20	5.92			-
Bacteria, Proteobacteria, Betaproteobacteria, Burkholderiales, Comamonadaceae, Pelomonas	4.35			-
Bacteria, Planctomycetes, Planctomycetacia, Planctomycetales, Planctomycetaceae, Isosphaera	5.10	CK	5.02	0.05
Bacteria, Actinobacteria, Actinobacteria, Micrococcales, Bogoriellaceae, Georgenia	4.19			-
Bacteria, Bacteroidetes, Sphingobacteriia, Sphingobacteriales, env_OPS_17	5.48			-

Bacteria, Proteobacteria, Gammaproteobacteria, Legionellales, Coxiellaceae	5.99			-
Bacteria, Bacteroidetes, Flavobacteriia, Flavobacteriales, Flavobacteriaceae, Gelidibacter	5.63			-
Bacteria, Proteobacteria, Gammaproteobacteria, Xanthomonadales, Xanthomonadaceae, Stenotrophomonas	5.56			-
Bacteria, Proteobacteria, Betaproteobacteria, Burkholderiales, Alcaligenaceae, Verticia	4.60			-
Bacteria, Proteobacteria, Alphaproteobacteria, Rhizobiales, Beijerinckiaceae, Methylovirgula	5.32			-
Bacteria, Actinobacteria, Actinobacteria, Frankiales	6.15			-
Bacteria, Chloroflexi, Chloroflexia, Chloroflexales, Roseiflexaceae, Roseiflexus	5.39			-
Bacteria, Proteobacteria, Alphaproteobacteria, Rhizobiales, Rhizobiaceae	5.82			-
Bacteria, Proteobacteria, Alphaproteobacteria, Rhizobiales, Roseiarcaceae, Roseiarcus	5.91			-
Bacteria, Proteobacteria, Gammaproteobacteria, Cellvibrionales	5.13			-
Bacteria, Firmicutes, Bacilli, Bacillales, Paenibacillaceae, Paenibacillus	6.41			-
Bacteria, Proteobacteria, Deltaproteobacteria, Myxococcales, Eel_36e1D6	0.00			-
Bacteria, Proteobacteria, Alphaproteobacteria, Rhizobiales, Bradyrhizobiaceae	5.40			-
Bacteria, Acidobacteria, Acidobacteria, Blastocatellales, Blastocatellaceae__Subgroup_4, Tellurimicrobium	4.85			-
Bacteria, Actinobacteria, Actinobacteria, Solirubrobacterales, Patulibacteraceae, Patulibacter	4.64			-
Bacteria, Actinobacteria, Actinobacteria, Propionibacteriales, Nocardioidaceae, Aeromicrobium	5.59	LM	5.23	0.05
Bacteria, Proteobacteria, Betaproteobacteria, Nitrosomonadales	6.74			-

Bacteria, Proteobacteria, Alphaproteobacteria, Rhizobiales, Methylobacteriaceae	4.95			-
Bacteria, Bacteroidetes, Cytophagia, Cytophagales, Cytophagaceae, Cytophaga	5.02			-
Bacteria, Actinobacteria, Actinobacteria, Frankiales, Sporichthyaceae, Sporichthya	4.67			-
Bacteria, Spirochaetae, Spirochaetes, Spirochaetales, Leptospiraceae	4.46			-
Bacteria, Bacteroidetes, Sphingobacteriia, Sphingobacteriales, Chitinophagaceae, Terrimonas	5.31			-
Bacteria, Proteobacteria, Alphaproteobacteria, Rhizobiales, Brucellaceae	4.84			-
Bacteria, Proteobacteria, Deltaproteobacteria, Bradymonadales	4.43			-
Bacteria, Planctomycetes, Planctomycetacia, Brocadiales	3.86			-
*Bacteria, Acidobacteria, Acidobacteria, Solibacterales, Solibacteraceae__Subgroup_3_*	6.83			-
Bacteria, Proteobacteria, Alphaproteobacteria, Rhizobiales, alphaI_cluster	4.82			-
Bacteria, Proteobacteria, Deltaproteobacteria, Bdellovibrionales, Bacteriovoracaceae, Peredibacter	5.06	LM	4.71	0.05
Bacteria, Armatimonadetes, Fimbriimonadia, Fimbriimonadales	5.31			-
Bacteria, Acidobacteria, Acidobacteria, Acidobacteriales, Acidobacteriaceae__Subgroup_1, Acidobacterium	6.19			-
Bacteria, Acidobacteria, Acidobacteria, Solibacterales, Solibacteraceae__Subgroup_3_, Bryobacter	6.63			-
Bacteria, Actinobacteria, Actinobacteria, Corynebacteriales, Nocardiaceae, Nocardia	5.02			-
Bacteria, Spirochaetae, Spirochaetes, Spirochaetales, Spirochaetaceae	5.19			-
Bacteria, Chlamydiae, Chlamydiae, Chlamydiales, Parachlamydiaceae, Neochlamydia	4.24			-

Bacteria, Firmicutes, Bacilli, Bacillales, Alicyclobacillaceae, Tumebacillus	5.35			-
Bacteria, Parcubacteria, Candidatus_Nomurabacteria	3.68			-
Bacteria, Bacteroidetes, Sphingobacteriia, Sphingobacteriales, Sphingobacteriaceae	5.33			-
Bacteria, Bacteroidetes, Sphingobacteriia, Sphingobacteriales, Chitinophagaceae, Chitinophaga	5.69			-
Bacteria, Proteobacteria, Alphaproteobacteria, Rhizobiales, KF_JG30_B3	5.26			-
Bacteria, Proteobacteria, Betaproteobacteria, Methylophilales	5.77			-
Bacteria, Proteobacteria, Gammaproteobacteria, Xanthomonadales	6.00			-
Bacteria, Proteobacteria, Alphaproteobacteria, Rhizobiales, Methylobacteriaceae, Meganema	4.48			-
Bacteria, Proteobacteria, Alphaproteobacteria, Rhodobacterales, Rhodobacteraceae	4.13			-
Bacteria, Bacteroidetes, Cytophagia, Cytophagales, Cytophagaceae, Adhaeribacter	0.00			-
Bacteria, Armatimonadetes, Chthonomonadetes	4.28			-
Bacteria, Actinobacteria, Actinobacteria, Corynebacteriales, Nocardiaceae, Smaragdicoccus	4.53			-
Bacteria, Proteobacteria, Alphaproteobacteria, Rhizobiales, Beijerinckiaceae	5.39			-
Bacteria, Proteobacteria, Alphaproteobacteria	5.40			-
Bacteria, Proteobacteria, Alphaproteobacteria, Rhodospirillales, Rhodospirillaceae	5.90			-
Bacteria, Proteobacteria, Alphaproteobacteria, Rhizobiales, Hyphomicrobiaceae, Hyphomicrobium	5.80			-
Bacteria, Proteobacteria, Gammaproteobacteria, Aeromonadales, Aeromonadaceae, Aeromonas	0.00			-

Bacteria, Firmicutes, Clostridia, Clostridiales, Family_XIII, Mogibacterium	4.27			-
Bacteria, Proteobacteria, ARKICE_90	4.33			-
Bacteria, Proteobacteria, Alphaproteobacteria, Caulobacterales, Hyphomonadaceae, Woodsholea	4.61			-
Bacteria, Proteobacteria, Alphaproteobacteria, Caulobacterales, Caulobacteraceae, Caulobacter	5.35	LM	5.07	0.05
Bacteria, Proteobacteria, Gammaproteobacteria, Xanthomonadales, Xanthomonadaceae, Tahibacter	4.51			-
Bacteria, Chloroflexi, Ktedonobacteria, B12_WMSP1	5.60	CK	5.10	0.05
Bacteria, Proteobacteria, Betaproteobacteria, Methylophilales, Methylophilaceae	5.39			-
Bacteria, Proteobacteria, Deltaproteobacteria, Bdellovibrionales, Bacteriovoracaceae	0.00			-
Bacteria, Proteobacteria, Betaproteobacteria	4.71	CK	4.80	0.04
Bacteria, Actinobacteria, Actinobacteria, Corynebacteriales	6.17			-
Bacteria, Proteobacteria, Alphaproteobacteria, Caulobacterales, Caulobacteraceae	6.06			-
Bacteria, Proteobacteria, Alphaproteobacteria, Rhizobiales, Xanthobacteraceae, Labrys	4.10			-
*Bacteria, TM6__Dependentiae_*	5.22			-
Bacteria, Proteobacteria, Betaproteobacteria, Burkholderiales, Burkholderiaceae, Limnobacter	5.18			-
Bacteria, Deinococcus_Thermus, Deinococci, Deinococcales, Deinococcaceae	4.23	LM	4.88	0.04
Bacteria, Bacteroidetes, Cytophagia, Cytophagales, Cytophagaceae, Leadbetterella	4.16			-
Bacteria, Proteobacteria, Deltaproteobacteria, Oligoflexales, 0319_6G20	5.85			-

Bacteria, Parcubacteria, Candidatus_Azambacteria	3.56			-
Bacteria, Latescibacteria	5.41			-
Bacteria, Armatimonadetes	4.49			-
Bacteria, Firmicutes	7.32			-
Bacteria, Ignavibacteriae	4.83			-
Bacteria, Bacteroidetes, Flavobacteriia, Flavobacteriales, Flavobacteriaceae, Chryseobacterium	5.57			-
Bacteria, GAL15	5.85			-
Bacteria, Planctomycetes, Phycisphaerae, Tepidisphaerales, Tepidisphaeraceae	6.10	CK	5.60	0.05
Bacteria, Chloroflexi, S085	5.18			-
Bacteria, Acidobacteria, Acidobacteria, Blastocatellales, Blastocatellaceae__Subgroup_4, Stenotrophobacter	4.43			-
Bacteria, Bacteroidetes, Sphingobacteriia, Sphingobacteriales, Chitinophagaceae, Filimonas	5.63			-
Bacteria, Planctomycetes, Phycisphaerae, Phycisphaerales, Phycisphaeraceae, SM1A02	4.28			-
Bacteria, Chloroflexi, Thermomicrobia, Sphaerobacterales, Sphaerobacteraceae, Nitrolancea	5.86	CK	5.30	0.05
Bacteria, Firmicutes, Clostridia, Clostridiales, Lachnospiraceae	3.56			-
Bacteria, Bacteroidetes, Bacteroidia, Bacteroidales, Porphyromonadaceae, Parabacteroides	0.00			-
Bacteria, Proteobacteria, Alphaproteobacteria, Rhizobiales, Xanthobacteraceae, Pseudoxanthobacter	4.33			-
Bacteria, Firmicutes, Limnochordia	4.70			-

Bacteria, Fibrobacteres, Fibrobacteria, Fibrobacterales	4.86			-
Bacteria, Proteobacteria, Alphaproteobacteria, Rickettsiales, SM2D12	5.63			-
Bacteria, Proteobacteria, Betaproteobacteria, Neisseriales, Neisseriaceae, Vitreoscilla	0.00			-
Bacteria, Firmicutes, Clostridia, Halanaerobiales	6.62			-
Bacteria, Proteobacteria, Deltaproteobacteria, Myxococcales, Haliangiaceae	6.01			-
Bacteria, Gemmatimonadetes, Gemmatimonadetes, Longimicrobiales, Longimicrobiaceae	5.38			-
Bacteria, Chlamydiae	5.05	CK	4.68	0.05
Bacteria, Actinobacteria, Actinobacteria, Micrococcales, Micrococcaceae, Arthrobacter	5.52			-
Bacteria, Actinobacteria, Actinobacteria, Frankiales, Frankiaceae	6.14			-
Bacteria, Proteobacteria, Gammaproteobacteria, Legionellales, Coxiellaceae, Coxiella	4.50			-
Bacteria, Firmicutes, Erysipelotrichia, Erysipelotrichales	5.37			-
Bacteria, Proteobacteria, Alphaproteobacteria, Rhizobiales, Phyllobacteriaceae, Mesorhizobium	6.48			-
Bacteria, Proteobacteria, Deltaproteobacteria, Myxococcales, Polyangiaceae	4.33			-
Bacteria, Proteobacteria, Alphaproteobacteria, Rhizobiales, Methylocystaceae	4.63			-
Bacteria, Actinobacteria, Actinobacteria, Frankiales, Geodermatophilaceae, Blastococcus	5.66			-
Bacteria, Proteobacteria, Deltaproteobacteria, Myxococcales, Myxococcaceae	3.93			-
Bacteria, Proteobacteria, Deltaproteobacteria, Myxococcales, Phaselicystidaceae, Phaselicystis	4.30			-

Bacteria, Bacteroidetes, Cytophagia, Cytophagales, Cytophagaceae, Chryseolinea	5.48	LM	5.03	0.05
Bacteria, Acidobacteria, Acidobacteria, Acidobacteriales, Acidobacteriaceae__Subgroup_1, Candidatus_Koribacter	5.37			-
Bacteria, Proteobacteria, Alphaproteobacteria, Rhodospirillales, MNC12	3.56			-
Bacteria, Proteobacteria, Gammaproteobacteria, Oceanospirillales, Hahellaceae	3.68			-
Bacteria, Firmicutes, Bacilli, Bacillales, Planococcaceae, Lysinibacillus	5.42			-
Bacteria, Proteobacteria, Alphaproteobacteria, Rhizobiales, Rhizobiales_Incertae_Sedis, Agaricicola	5.33			-
Bacteria, Firmicutes, Bacilli, Lactobacillales, Carnobacteriaceae, Carnobacterium	4.41			-
Bacteria, Proteobacteria, Betaproteobacteria, Burkholderiales, Alcaligenaceae, Eoetvoesia	5.26			-
Bacteria, Actinobacteria, Actinobacteria, Streptosporangiales, Streptosporangiaceae, Nonomuraea	4.62			-
Bacteria, Bacteroidetes, Sphingobacteriia, Sphingobacteriales, Sphingobacteriaceae, Pedobacter	5.90			-
Bacteria, Gemmatimonadetes	7.35			-
Bacteria, Parcubacteria	6.41			-
Bacteria, Acidobacteria, Acidobacteria, Blastocatellales	6.19			-
Bacteria, Proteobacteria, Deltaproteobacteria	3.72			-
Bacteria, Proteobacteria, Alphaproteobacteria, Rhizobiales, Bradyrhizobiaceae, Bradyrhizobium	6.60			-
Bacteria, Actinobacteria, Actinobacteria, Solirubrobacterales, FFCH11085	4.52			-
Bacteria, Actinobacteria, Actinobacteria, Frankiales, Acidothermaceae	6.18			-

Bacteria, Proteobacteria, Deltaproteobacteria, Bdellovibrionales, Bdellovibrionaceae	5.98			-
Bacteria, Proteobacteria, Gammaproteobacteria, Pseudomonadales, Moraxellaceae, Perlucidibaca	4.42			-
Bacteria, Proteobacteria, Alphaproteobacteria, Rhizobiales, Beijerinckiaceae, Methylorosula	4.62	LM	4.55	0.05
Bacteria, Actinobacteria, Actinobacteria, Acidimicrobiales, Iamiaceae, Iamia	4.98			-
Bacteria, Actinobacteria, Actinobacteria, Acidimicrobiales, OM1_clade	4.02			-
Bacteria, Proteobacteria, Betaproteobacteria, Nitrosomonadales, Nitrosomonadaceae	6.68			-
Bacteria, Proteobacteria, Gammaproteobacteria, Legionellales, Coxiellaceae, Rickettsiella	4.89			-
Bacteria, Proteobacteria, Alphaproteobacteria, Rhizobiales, Rhizobiaceae, Kaistia	4.58			-
Bacteria, Bacteroidetes, Cytophagia, Cytophagales	6.36	LM	5.91	0.05
Bacteria, Chloroflexi, Thermomicrobia, JG30_KF_CM45	6.04	CK	5.48	0.05
Bacteria, Bacteroidetes, Sphingobacteriia, Sphingobacteriales, Chitinophagaceae, Parasegetibacter	4.04			-
Bacteria, Proteobacteria, Alphaproteobacteria, Caulobacterales	6.45			-
Bacteria, Bacteroidetes, Cytophagia, Cytophagales, Cytophagaceae	6.20	LM	5.75	0.05
Bacteria, Proteobacteria, Alphaproteobacteria, Rhodospirillales, MND8	4.16			-
Bacteria, Proteobacteria, Deltaproteobacteria, NB1_j	4.44			-
Bacteria, Proteobacteria, Betaproteobacteria, Burkholderiales, Alcaligenaceae, Paralcaligenes	5.36			-
Bacteria, Proteobacteria, Alphaproteobacteria, Rhizobiales, Rhizobiales_Incertae_Sedis, Nordella	5.16			-

Bacteria, FCPU426	4.67			-
Bacteria, Firmicutes, Bacilli, Bacillales, Paenibacillaceae, Aneurinibacillus	4.43			-
Bacteria, Proteobacteria, Alphaproteobacteria, Rhizobiales, Bradyrhizobiaceae, Bosea	4.63	LM	4.78	0.05
Bacteria, Proteobacteria, Betaproteobacteria, Burkholderiales, Comamonadaceae, Rhizobacter	4.79			-
Bacteria, Proteobacteria, Gammaproteobacteria, Cellvibrionales, Cellvibrionaceae, Simiduia	0.00			-
Bacteria, Proteobacteria, Deltaproteobacteria, Myxococcales, Blfdi19	4.72			-
Bacteria, Actinobacteria, Actinobacteria, Acidimicrobiales	6.19			-
Bacteria, Armatimonadetes, Chthonomonadetes, Chthonomonadales	4.28			-
Bacteria, Proteobacteria, Betaproteobacteria, Methylophilales, Methylophilaceae, Methylotenera	4.88			-
Bacteria, Proteobacteria, Deltaproteobacteria, Oligoflexales	5.96			-
Bacteria, Proteobacteria, Betaproteobacteria, Rhodocyclales	4.82			-
Bacteria, Fibrobacteres, Fibrobacteria	4.86			-
Bacteria, Proteobacteria, Gammaproteobacteria, Xanthomonadales, Xanthomonadaceae, Rhodanobacter	7.84			-
Bacteria, Proteobacteria, Betaproteobacteria, Nitrosomonadales, Nitrosomonadaceae, Nitrosospira	5.78			-
Bacteria, Proteobacteria, Deltaproteobacteria, Desulfuromonadales, Geobacteraceae, Geobacter	0.00			-
Bacteria, Bacteroidetes, Sphingobacteriia, Sphingobacteriales, Chitinophagaceae, Flavihumibacter	4.76	LM	4.63	0.05
Bacteria, Firmicutes, Bacilli, Bacillales, Thermoactinomycetaceae	4.95			-

Bacteria, Peregrinibacteria	4.63	LM	4.56	0.05
Bacteria, Proteobacteria, Gammaproteobacteria	6.51			-
Bacteria, Actinobacteria, Actinobacteria	5.78			-
Bacteria, Chloroflexi, SBR2076	5.55			-
Bacteria, Parcubacteria, Candidatus_Jorgensenbacteria	4.26			-
Bacteria, Proteobacteria, Alphaproteobacteria, Rhodospirillales, Rhodospirillaceae, Dongia	5.26			-
Bacteria, Acidobacteria	7.53			-
Bacteria, Chloroflexi, Chloroflexia, Chloroflexales, FFCH7168	4.40			-
Bacteria, Actinobacteria, Actinobacteria, Acidimicrobiales, Acidimicrobiales_Incertae_Sedis	3.72			-
Bacteria, Proteobacteria, Betaproteobacteria, Hydrogenophilales, Hydrogenophilaceae	4.75			-
Bacteria, Actinobacteria, Actinobacteria, Micromonosporales, Micromonosporaceae, Dactylosporangium	4.68			-
Bacteria, Cyanobacteria, Cyanobacteria, SubsectionIII, FamilyI_o__SubsectionIII	4.54			-
Bacteria, Actinobacteria, Actinobacteria, Micrococcales, Intrasporangiaceae	6.32			-
Bacteria, Proteobacteria, Gammaproteobacteria, Oceanospirillales, Oceanospirillaceae	5.33			-
Bacteria, Bacteroidetes, Flavobacteriia, Flavobacteriales, Flavobacteriaceae, Aequorivita	5.29			-
Bacteria, Chlamydiae, Chlamydiae, Chlamydiales, cvE6	4.94			-
Bacteria, Firmicutes, Limnochordia, Limnochordales, Limnochordaceae	4.70			-

Bacteria, Proteobacteria, Gammaproteobacteria, Xanthomonadales, Xanthomonadaceae, Thermomonas	5.20	LM	4.86	0.05
Bacteria, Ignavibacteriae, Ignavibacteria	4.83			-
Bacteria, Proteobacteria, Deltaproteobacteria, Oligoflexales, Oligoflexaceae	5.30			-
Bacteria, Proteobacteria, Deltaproteobacteria, Myxococcales, 27F_1492R	4.65			-
Bacteria, Fibrobacteres, Fibrobacteria, Fibrobacterales, Fibrobacteraceae	4.63			-
Bacteria, Actinobacteria, Actinobacteria, Propionibacteriales, Nocardioidaceae, Marmoricola	6.10			-
Bacteria, Actinobacteria, Actinobacteria, Micrococcales, Microbacteriaceae, Leucobacter	3.58			-
Bacteria, Chloroflexi, Ktedonobacteria, B10_SB3A	5.20	CK	4.85	0.05
Bacteria, Chloroflexi, Caldilineae, Caldilineales, Caldilineaceae	4.52			-
Bacteria, Bacteroidetes, Sphingobacteriia, Sphingobacteriales, Sphingobacteriaceae, Pseudopedobacter	4.39	LM	5.12	0.04
Bacteria, Saccharibacteria, Candidatus_Saccharimonas	4.33			-
Bacteria, Actinobacteria, Actinobacteria, Pseudonocardiales, Pseudonocardiaceae, Amycolatopsis	5.33			-
Bacteria, Acidobacteria, Acidobacteria, Acidobacteriales, Acidobacteriaceae__Subgroup_1	7.12			-
Bacteria, Firmicutes, Clostridia, Clostridiales, Family_XIII	4.27			-
Bacteria, Actinobacteria, Actinobacteria, Solirubrobacterales	4.54			-
Bacteria, Actinobacteria, Actinobacteria, Micrococcales, Microbacteriaceae, Leifsonia	5.64			-
Bacteria, Actinobacteria, Actinobacteria, Pseudonocardiales, Pseudonocardiaceae	5.64			-

Bacteria, Actinobacteria, Actinobacteria, Micromonosporales, Micromonosporaceae, Hamadaea	4.78			-
Bacteria, Acidobacteria, Acidobacteria, Blastocatellales, Blastocatellaceae__Subgroup_4, RB41	5.24			-
Bacteria, Acidobacteria, Acidobacteria, Acidobacteriales	7.22			-
Bacteria, Nitrospirae, Nitrospira	6.12			-
Bacteria, Armatimonadetes, Chthonomonadetes, Chthonomonadales, Chthonomonadaceae, Chthonomonas	4.28			-
Bacteria, Actinobacteria, Actinobacteria, Micrococcales, Microbacteriaceae, Humibacter	5.67			-
Bacteria, Acidobacteria, Acidobacteria, Solibacterales, Solibacteraceae__Subgroup_3_, Paludibaculum	4.57			-
Bacteria, Fibrobacteres	4.86			-
Bacteria, Verrucomicrobia, Verrucomicrobiae, Verrucomicrobiales, Verrucomicrobiaceae, Verrucomicrobium	4.07			-
Bacteria, Proteobacteria, Gammaproteobacteria, Xanthomonadales, Xanthomonadaceae, Frateuria	6.30			-
Bacteria, Proteobacteria, Deltaproteobacteria, Bradymonadales, Bradymonadaceae, Bradymonas	4.43			-
Bacteria, Spirochaetae, Spirochaetes, Spirochaetales, Leptospiraceae, Turneriella	4.46			-
Bacteria, Cyanobacteria	5.91			-
Bacteria, Proteobacteria, Alphaproteobacteria, Rhizobiales, Hyphomicrobiaceae, Rhodomicrobium	4.16			-
Bacteria, Actinobacteria, Actinobacteria, Kineosporiales, Kineosporiaceae	5.69	CK	5.19	0.05
Bacteria, Proteobacteria, Gammaproteobacteria, Thiotrichales, Thiotrichaceae, Beggiatoa	0.00			-
Bacteria, Bacteroidetes, Flavobacteriia, Flavobacteriales, Flavobacteriaceae, Moheibacter	4.98			-

Bacteria, Proteobacteria, Alphaproteobacteria, Sphingomonadales, Sphingomonadaceae	7.35			-
Bacteria, Proteobacteria, Alphaproteobacteria, Rhizobiales, Bradyrhizobiaceae, Rhodopseudomonas	5.41			-
Bacteria, Proteobacteria, Alphaproteobacteria, Rhodospirillales, Rhodospirillaceae, Ferrovibrio	4.07			-
Bacteria, Chloroflexi, Ktedonobacteria, Ktedonobacterales	5.32			-
Bacteria, Bacteroidetes, Flavobacteriia	6.74			-
Bacteria, Proteobacteria, Alphaproteobacteria, Rhodospirillales, Rhodospirillaceae, Inquilinus	4.30			-
Bacteria, Proteobacteria, Betaproteobacteria, Nitrosomonadales, Nitrosomonadaceae, Nitrosomonas	4.46			-
Bacteria, Actinobacteria, Actinobacteria, Micromonosporales	5.88			-
Bacteria, Firmicutes, Bacilli, Bacillales, Alicyclobacillaceae, Alicyclobacillus	4.96			-
Bacteria, Bacteroidetes, Flavobacteriia, Flavobacteriales, Flavobacteriaceae, Flavobacterium	6.02			-
Bacteria, Proteobacteria, Alphaproteobacteria, Sphingomonadales, DSSF69	4.69			-
Bacteria, Proteobacteria, Gammaproteobacteria, Enterobacteriales, Enterobacteriaceae	5.55			-
Bacteria, Acidobacteria, Acidobacteria, Holophagales, Holophagaceae, Geothrix	4.71			-
Bacteria, Proteobacteria, Betaproteobacteria, Burkholderiales, Comamonadaceae, Ottowia	5.25			-
Bacteria, Bacteroidetes, Bacteroidia, Bacteroidales	5.09			-
Bacteria, Elusimicrobia	5.46			-
Bacteria, Proteobacteria, Betaproteobacteria, B1_7BS	4.15			-

Bacteria, Proteobacteria, Alphaproteobacteria, Rhizobiales, Hyphomicrobiaceae, Pedomicrobium	4.72			-
Bacteria, Actinobacteria, Actinobacteria, Solirubrobacterales, Solirubrobacteraceae	4.29			-
Bacteria, Proteobacteria, Gammaproteobacteria, Enterobacteriales, Enterobacteriaceae, Serratia	3.91			-
Bacteria, Chloroflexi, JG30_KF_CM66	5.76			-
Bacteria, Actinobacteria, Actinobacteria, Streptosporangiales, Streptosporangiaceae, Sphaerisporangium	4.98			-
Bacteria, BRC1	4.73			-
Bacteria, Planctomycetes	6.46	CK	5.80	0.05
Bacteria, Proteobacteria, Alphaproteobacteria, Rhodospirillales, Acetobacteraceae, Acidicaldus	5.02			-
Bacteria, Chloroflexi, Thermomicrobia, Sphaerobacterales, Sphaerobacteraceae	5.86	CK	5.29	0.05
Bacteria, Actinobacteria, Actinobacteria, Micrococcales, Demequinaceae	4.43			-
Bacteria, Actinobacteria, Actinobacteria, Frankiales, Nakamurellaceae, Nakamurella	5.61			-
Bacteria, Firmicutes, Clostridia, Clostridiales, Family_XVII	4.47			-
Bacteria, Actinobacteria, Actinobacteria, Solirubrobacterales, TM146	4.57			-
Bacteria, Proteobacteria, Gammaproteobacteria, HTA4	4.88			-
Bacteria, Proteobacteria, Alphaproteobacteria, Rhizobiales, Roseiarcaceae	5.91			-
Bacteria, Proteobacteria, Gammaproteobacteria, Legionellales	6.01			-
Bacteria, Chloroflexi, KD4_96	6.32			-

Bacteria, Firmicutes, Bacilli, Lactobacillales, Enterococcaceae	6.03			-
Bacteria, Actinobacteria, Actinobacteria, Streptosporangiales, Thermomonosporaceae, Actinomadura	5.57			-
Bacteria, Actinobacteria, Actinobacteria, Micrococcales	6.89			-
Bacteria, Proteobacteria, Alphaproteobacteria, Caulobacterales, Caulobacteraceae, Phenylobacterium	5.95			-
Bacteria, Armatimonadetes, Fimbriimonadia	5.31			-
Bacteria, Actinobacteria, Actinobacteria, Acidimicrobiales, Acidimicrobiaceae, Ilumatobacter	4.44			-
Bacteria, Chloroflexi, Ktedonobacteria, Ktedonobacterales, HSB_OF53_F07	6.40			-
Bacteria, Proteobacteria, Gammaproteobacteria, Oceanospirillales, Halomonadaceae, Halomonas	4.04			-
Bacteria, Proteobacteria, Alphaproteobacteria, Rhodospirillales, JG37_AG_20	5.77			-
Bacteria, Microgenomates	5.46			-
Bacteria, Proteobacteria, JTB23	4.19			-
Bacteria, Proteobacteria, Deltaproteobacteria, Myxococcales, Haliangiaceae, Haliangium	6.01			-
Bacteria, Parcubacteria, Candidatus_Magasanikbacteria	4.75			-
Bacteria, Verrucomicrobia, Spartobacteria, Chthoniobacterales, Chthoniobacteraceae, Chthoniobacter	4.98			-
Bacteria, Actinobacteria, Actinobacteria, Streptomycetales, Streptomycetaceae	6.17			-
Bacteria, Firmicutes, Bacilli	3.58			-
Bacteria, Proteobacteria, Gammaproteobacteria, Xanthomonadales, Solimonadaceae, Solimonas	5.60	LM	5.21	0.04

Bacteria, Proteobacteria, Deltaproteobacteria, Desulfuromonadales	0.00			-
Bacteria, Proteobacteria, Betaproteobacteria, Burkholderiales, Alcaligenaceae, Candidimonas	6.40			-
Bacteria, Proteobacteria, Gammaproteobacteria, Cellvibrionales, Cellvibrionaceae	5.11			-
Bacteria, Bacteroidetes, Sphingobacteriia, Sphingobacteriales, Chitinophagaceae, Taibaiella	6.76			-
Bacteria, Actinobacteria, Actinobacteria, Micrococcales, Dermacoccaceae, Flexivirga	5.63	CK	5.24	0.05
Bacteria, Actinobacteria, Actinobacteria, Micrococcales, Cellulomonadaceae, Actinotalea	4.27			-
Bacteria, Proteobacteria, Alphaproteobacteria, Rhizobiales, Rhizobiaceae, Rhizobium	5.80			-
Bacteria, Proteobacteria, Alphaproteobacteria, Sphingomonadales	5.75			-
Bacteria, Chloroflexi, Chloroflexia	5.43			-
Bacteria, Cyanobacteria, Cyanobacteria, SubsectionIII, FamilyI_o__SubsectionIII, Phormidium	4.04			-
Bacteria, Planctomycetes, Planctomycetacia, Brocadiales, Brocadiaceae, Candidatus_Kuenenia	3.86			-
Bacteria, Proteobacteria, Alphaproteobacteria, Sphingomonadales, Sphingomonadaceae, Novosphingobium	5.28			-
Bacteria, Acidobacteria, Acidobacteria, Blastocatellales, Blastocatellaceae__Subgroup_4	6.09			-
Bacteria, Spirochaetae	5.22			-
Bacteria, Proteobacteria, Deltaproteobacteria, Myxococcales	6.01			-
Bacteria, Actinobacteria, Actinobacteria, Micrococcales, Dermacoccaceae	5.63	CK	5.25	0.05
Bacteria, Proteobacteria, Gammaproteobacteria, Xanthomonadales, Xanthomonadales_Incertae_Sedis	6.37			-

Bacteria, Planctomycetes, BD7_11	4.46			-
Bacteria, Proteobacteria, Betaproteobacteria, Burkholderiales, Comamonadaceae, Ramlibacter	6.03			-
Bacteria, Chloroflexi, Ktedonobacteria, Ktedonobacterales, Thermosporotrichaceae	5.58			-
Bacteria, Proteobacteria, Betaproteobacteria, Burkholderiales, Comamonadaceae, Comamonas	0.00			-
Bacteria, Actinobacteria, Actinobacteria, Gaiellales, Gaiellaceae, Gaiella	6.00			-
Bacteria, Proteobacteria, Gammaproteobacteria, Oceanospirillales	5.96			-
Bacteria, Firmicutes, Clostridia, Clostridiales	3.98			-
Bacteria, Verrucomicrobia, Verrucomicrobiae, Verrucomicrobiales, Verrucomicrobiaceae	4.35			-
Bacteria, Verrucomicrobia, Opitutae, Opitutales, Opitutaceae	5.74			-
Bacteria, Gemmatimonadetes, Gemmatimonadetes, Gemmatimonadales, Gemmatimonadaceae, Gemmatimonas	7.06			-
Bacteria, Elusimicrobia, Elusimicrobia, Lineage_IV	5.36			-
Bacteria, Proteobacteria, Alphaproteobacteria, Rhizobiales, Phyllobacteriaceae, Aquamicrobium	5.42			-
Bacteria, Proteobacteria, Deltaproteobacteria, Bradymonadales, Bradymonadaceae	4.43			-
Bacteria, Actinobacteria, Actinobacteria, Micromonosporales, Micromonosporaceae, Luedemannella	4.99			-
Bacteria, Proteobacteria, Deltaproteobacteria, Myxococcales, Sandaracinaceae, Sandaracinus	4.97	LM	4.85	0.05
Bacteria, Planctomycetes, Planctomycetacia, Planctomycetales, Planctomycetaceae	6.00	CK	5.19	0.05
Bacteria, Gemmatimonadetes, Gemmatimonadetes, Gemmatimonadales, Gemmatimonadaceae	7.01			-

Bacteria, Firmicutes, Bacilli, Lactobacillales, Streptococcaceae, Lactococcus	6.02			-
Bacteria, Chlorobi, Chlorobia, Chlorobiales, SJA_28	0.00			-
Bacteria, Actinobacteria, Actinobacteria, Solirubrobacterales, Solirubrobacteraceae, Solirubrobacter	4.29			-
Bacteria, Verrucomicrobia, Spartobacteria, Chthoniobacterales, DA101_soil_group	6.35	CK	5.72	0.05
Bacteria, Actinobacteria, Actinobacteria, Propionibacteriales, Nocardioidaceae, Kribbella	5.47			-
Bacteria, Bacteroidetes, Sphingobacteriia, Sphingobacteriales, PHOS_HE51	0.00			-
Bacteria, Bacteroidetes, Sphingobacteriia, Sphingobacteriales, Chitinophagaceae, Sediminibacterium	5.31	LM	4.87	0.05
Bacteria, Proteobacteria	5.87			-
Bacteria, Proteobacteria, Betaproteobacteria, Burkholderiales, Alcaligenaceae, Castellaniella	6.19			-
Bacteria, Gemmatimonadetes, Gemmatimonadetes, Gemmatimonadales	7.35			-
Bacteria, Chloroflexi, Ktedonobacteria, Ktedonobacterales, 1921_3	5.66			-
Bacteria, Bacteroidetes, Cytophagia	6.36	LM	5.88	0.05
Bacteria, Bacteroidetes, Flavobacteriia, Flavobacteriales, Cryomorphaceae, Wandonia	5.82	LM	5.47	0.05
Bacteria, Proteobacteria, Alphaproteobacteria, Caulobacterales, Caulobacteraceae, Asticcacaulis	5.61	LM	5.10	0.05
Bacteria, Proteobacteria, Gammaproteobacteria, Xanthomonadales, Nevskiaceae, Nevskia	4.46			-
Bacteria, Proteobacteria, Gammaproteobacteria, Xanthomonadales, Solimonadaceae	5.65	LM	5.27	0.05
Bacteria, Deinococcus_Thermus, Deinococci, Deinococcales, Trueperaceae, Truepera	5.76			-

Bacteria, Proteobacteria, Alphaproteobacteria, Caulobacterales, Caulobacteraceae, Brevundimonas	5.56			-
Bacteria, Chloroflexi, Chloroflexia, Chloroflexales, Roseiflexaceae	5.39			-
Bacteria, Bacteroidetes, Flavobacteriia, Flavobacteriales, Cryomorphaceae	4.21			-
Bacteria, Proteobacteria, Deltaproteobacteria, Myxococcales, Polyangiaceae, Sorangium	5.00			-
Bacteria, Acidobacteria, Acidobacteria, Holophagales, Holophagaceae, Holophaga	6.26			-
Bacteria, Chloroflexi, Ktedonobacteria, Ktedonobacterales, BacC_u_018	5.19			-
Bacteria, Actinobacteria, Actinobacteria, Micrococcales, Cellulomonadaceae	4.27			-
Bacteria, Spirochaetae, Spirochaetes, Spirochaetales	5.22			-
Bacteria, Proteobacteria, Alphaproteobacteria, Rhodospirillales	6.11			-
Bacteria, Verrucomicrobia, Verrucomicrobiae, Verrucomicrobiales, Verrucomicrobiaceae, Luteolibacter	3.68			-
Bacteria, Proteobacteria, Alphaproteobacteria, Rhodospirillales, Rhodospirillales_Incertae_Sedis, Reyranella	6.18			-
Bacteria, FBP	5.80			-
Bacteria, Armatimonadetes, Fimbriimonadia, Fimbriimonadales, Fimbriimonadaceae	5.31			-
Bacteria, Chlamydiae, Chlamydiae, Chlamydiales, Parachlamydiaceae	4.24			-
Bacteria, Verrucomicrobia, Opitutae, Opitutales, Opitutaceae, Opitutus	5.74			-
Bacteria, Planctomycetes, Planctomycetacia, Planctomycetales	6.20	CK	5.51	0.05
Bacteria, Proteobacteria, Gammaproteobacteria, Xanthomonadales, Xanthomonadaceae, Mizugakiibacter	7.65			-

Bacteria, Planctomycetes, Phycisphaerae, Phycisphaerales, Phycisphaeraceae, Phycisphaera	0.00			-
Bacteria, Actinobacteria, Actinobacteria, Propionibacteriales, Nocardioidaceae	6.48			-
Bacteria, Proteobacteria, Betaproteobacteria, Burkholderiales, Oxalobacteraceae, Duganella	4.53	LM	4.70	0.04
Bacteria, Verrucomicrobia, Opitutae, Opitutales	5.74			-
Bacteria, Parcubacteria, Candidatus_Campbellbacteria	0.00			-
Bacteria, Bacteroidetes, Flavobacteriia, Flavobacteriales	6.74			-
Bacteria, Proteobacteria, Gammaproteobacteria, Methylococcales, Methylococcaceae, Methylocaldum	4.04			-
Bacteria, Proteobacteria, Alphaproteobacteria, Rhodobacterales	5.14			-
Bacteria, Proteobacteria, Deltaproteobacteria, Myxococcales, Archangiaceae, Anaeromyxobacter	5.55			-
Bacteria, Proteobacteria, Gammaproteobacteria, Legionellales, Coxiellaceae, Aquicella	5.98			-
Bacteria, Firmicutes, Bacilli, Bacillales, Sporolactobacillaceae	6.41	CK	6.09	0.05
Bacteria, Proteobacteria, Gammaproteobacteria, Xanthomonadales, Xanthomonadaceae, Rudaea	4.26			-
Bacteria, Firmicutes, Bacilli, Bacillales	7.07			-
Bacteria, Proteobacteria, Deltaproteobacteria, Myxococcales, BIrii41	4.92	LM	4.70	0.04
Bacteria, Proteobacteria, Betaproteobacteria, Burkholderiales, Alcaligenaceae, Achromobacter	5.06			-
Bacteria, Firmicutes, Bacilli, Bacillales, Paenibacillaceae, Brevibacillus	6.35			-
Bacteria, Actinobacteria, Actinobacteria, Micrococcales, Promicromonosporaceae, Cellulosimicrobium	4.46			-

Bacteria, Chloroflexi, Ktedonobacteria, Thermogemmatisporales	5.40			-
Bacteria, Proteobacteria, Betaproteobacteria, Rhodocyclales, Rhodocyclaceae	4.72			-
Bacteria, Proteobacteria, Deltaproteobacteria, Myxococcales, Nannocystaceae	3.56			-
Bacteria, Acidobacteria, Acidobacteria, Blastocatellales, Blastocatellaceae__Subgroup_4, 11_24	4.95			-
Bacteria, Actinobacteria, Actinobacteria, Micrococcales, Promicromonosporaceae	4.46			-
Bacteria, Bacteroidetes, Sphingobacteriia, Sphingobacteriales, Chitinophagaceae, Parafilimonas	5.09	LM	4.83	0.05
Bacteria, Bacteroidetes, Sphingobacteriia, Sphingobacteriales, Chitinophagaceae, Niastella	4.90			-
Bacteria, Actinobacteria, Actinobacteria, Solirubrobacterales, Patulibacteraceae	4.64			-
Bacteria, Chloroflexi, Chloroflexia, Chloroflexales	5.43			-
Bacteria, Bacteroidetes, Flavobacteriia, Flavobacteriales, Flavobacteriaceae, Pricia	6.29	LM	5.98	0.05
Bacteria, Proteobacteria, Alphaproteobacteria, Rickettsiales	5.72			-
Bacteria, Chloroflexi, Ktedonobacteria, Thermogemmatisporales, 1921_2	5.40			-
Bacteria, Actinobacteria, Actinobacteria, Streptosporangiales, Streptosporangiaceae, Microbispora	5.96			-
Bacteria, Cyanobacteria, Cyanobacteria, Vampirovibrionales	4.83			-
Bacteria, Verrucomicrobia, Verrucomicrobiae	4.35			-
Bacteria, Actinobacteria, Actinobacteria, Corynebacteriales, Nocardiaceae	5.33			-
Bacteria, Planctomycetes, Planctomycetacia	6.20	CK	5.55	0.05

Bacteria, Bacteroidetes, Sphingobacteriia, Sphingobacteriales, Saprospiraceae	4.87			-
Bacteria, Proteobacteria, Alphaproteobacteria, Rhodobacterales, Rhodobacteraceae, Rhodobacter	4.28			-
Bacteria, Chloroflexi, TK10	6.13			-
Bacteria, Actinobacteria, Actinobacteria, Corynebacteriales, Mycobacteriaceae	6.10			-
Bacteria, Chlorobi	5.73			-
Bacteria, Proteobacteria, Gammaproteobacteria, Pseudomonadales, Pseudomonadaceae, Pseudomonas	5.90	LM	5.39	0.05
Bacteria, Bacteroidetes, Bacteroidia	5.09			-
Bacteria, Actinobacteria, Actinobacteria, Micrococcales, Microbacteriaceae	6.08			-
Bacteria, Gemmatimonadetes, Gemmatimonadetes, Gemmatimonadales, Gemmatimonadaceae, Gemmatirosa	5.69			-
Bacteria, Actinobacteria, Actinobacteria, Acidimicrobiales, Acidimicrobiales_Incertae_Sedis, Candidatus_Microthrix	3.72			-
*Bacteria, Proteobacteria, Deltaproteobacteria, SAR324_clade_Marine_group_B_*	4.28			-
Bacteria, Chloroflexi, Gitt_GS_136	5.14	CK	4.75	0.05
Bacteria, Acidobacteria, Acidobacteria, Blastocatellales, Blastocatellaceae__Subgroup_4, DS_100	4.28			-
Bacteria, Proteobacteria, Gammaproteobacteria, Enterobacteriales, Enterobacteriaceae, Kluyvera	5.54			-
Bacteria, Proteobacteria, Betaproteobacteria, Neisseriales	4.19			-
Bacteria, Proteobacteria, Alphaproteobacteria, Rickettsiales, Rickettsiales_Incertae_Sedis, Candidatus_Odyssella	4.70			-
Bacteria, Deinococcus_Thermus, Deinococci, Deinococcales	5.77			-

Bacteria, Proteobacteria, Betaproteobacteria, Burkholderiales, Alcaligenaceae	6.08			-
Bacteria, Bacteroidetes, Sphingobacteriia, Sphingobacteriales, KD3_93	4.28			-
Bacteria, Proteobacteria, Betaproteobacteria, Burkholderiales, Comamonadaceae, Xenophilus	5.12			-
Bacteria, Bacteroidetes, Sphingobacteriia, Sphingobacteriales, NS11_12_marine_group	5.06			-
Bacteria, Proteobacteria, Gammaproteobacteria, Xanthomonadales, Xanthomonadaceae, Pseudoxanthomonas	4.79	LM	4.74	0.05
Bacteria, Proteobacteria, Gammaproteobacteria, Xanthomonadales, Solimonadaceae, Polycyclovorans	4.62			-
Bacteria, Spirochaetae, Spirochaetes, Spirochaetales, Spirochaetaceae, Spirochaeta_2	5.19			-
Bacteria, Nitrospirae	6.12			-
Bacteria, Proteobacteria, Betaproteobacteria, Methylophilales, Methylophilaceae, Methylobacillus	5.44	LM	5.19	0.05
Bacteria, Firmicutes, Bacilli, Bacillales, Alicyclobacillaceae	5.44			-
Bacteria, Verrucomicrobia, Spartobacteria, Chthoniobacterales, Xiphinematobacteraceae, Candidatus_Xiphinematobacter	4.92			-
Bacteria, Bacteroidetes, Sphingobacteriia, Sphingobacteriales, Sphingobacteriaceae, Parapedobacter	4.30			-
Bacteria, Fibrobacteres, Fibrobacteria, Fibrobacterales, Fibrobacteraceae, possible_genus_04	4.48			-
Bacteria, Cyanobacteria, Cyanobacteria, SubsectionIII, FamilyI_o__SubsectionIII, Microcoleus	3.95			-
Bacteria, Bacteroidetes, Cytophagia, Cytophagales, Cytophagaceae, Emticicia	4.16			-
Bacteria, Firmicutes, Clostridia, Clostridiales, Clostridiaceae_1, Clostridium_sensu_stricto_1	5.62			-
Bacteria, Firmicutes, Bacilli, Bacillales, Sporolactobacillaceae, Sporolactobacillus	6.32	CK	6.00	0.05

Bacteria, Firmicutes, Clostridia, Clostridiales, Clostridiaceae_1, Clostridium_sensu_stricto_9	4.25			-
Bacteria, Chloroflexi, Ktedonobacteria, Ktedonobacterales, JG30a_KF_32	6.19			-
Bacteria, Chlamydiae, Chlamydiae	5.05	CK	4.74	0.05
Bacteria, Proteobacteria, Alphaproteobacteria, Rhodospirillales, Acetobacteraceae, Acidisoma	4.26			-
Bacteria, Proteobacteria, Alphaproteobacteria, Rhizobiales, Phyllobacteriaceae, Aminobacter	5.82			-
Bacteria, Bacteroidetes, Bacteroidia, Bacteroidales, Porphyromonadaceae, Dysgonomonas	0.00			-
Bacteria, Chloroflexi, Anaerolineae, Anaerolineales	5.96			-
Bacteria, Bacteroidetes, Sphingobacteriia, Sphingobacteriales, Chitinophagaceae, Flavisolibacter	6.08			-
Bacteria, Chlamydiae, Chlamydiae, Chlamydiales, Simkaniaceae	4.23			-
Bacteria, Chloroflexi, Anaerolineae, Anaerolineales, Anaerolineaceae	5.95			-
Bacteria, Actinobacteria, Actinobacteria, Solirubrobacterales, Elev_16S_1332	4.92			-
Bacteria, Bacteroidetes, Sphingobacteriia, Sphingobacteriales, Chitinophagaceae, Ferruginibacter	4.73			-
Bacteria, Chloroflexi	5.33			-
Bacteria, Acidobacteria, Acidobacteria, Holophagales	6.52	LM	6.08	0.05
Bacteria, Actinobacteria, Actinobacteria, Solirubrobacterales, 0319_6M6	4.91			-
Bacteria, Proteobacteria, Betaproteobacteria, Hydrogenophilales	4.75			-
Bacteria, Proteobacteria, Alphaproteobacteria, Rhizobiales, Rhodobiaceae	5.50			-

Bacteria, Proteobacteria, Alphaproteobacteria, Sphingomonadales, Sphingomonadaceae, Rhizorhapis	4.67			-
Bacteria, Bacteroidetes, Flavobacteriia, Flavobacteriales, Flavobacteriaceae, Empedobacter	4.38			-
Bacteria, Firmicutes, Bacilli, Bacillales, Family_XII_o__Bacillales	4.76			-
Bacteria, Proteobacteria, Alphaproteobacteria, Caulobacterales, Hyphomonadaceae	4.16			-
Bacteria, Chloroflexi, JG37_AG_4	6.86			-
Bacteria, Firmicutes, Clostridia	4.82			-
Bacteria, Chloroflexi, SHA_26	4.63			-
Bacteria, Chloroflexi, Caldilineae	4.52			-
Bacteria, Bacteroidetes, Sphingobacteriia, Sphingobacteriales, Chitinophagaceae, Heliimonas	4.16			-
Bacteria, Planctomycetes, Planctomycetacia, Brocadiales, Brocadiaceae	3.86			-
Bacteria, Omnitrophica	3.58			-
Bacteria, Proteobacteria, Betaproteobacteria, Burkholderiales, Oxalobacteraceae, Paucimonas	4.41			-
Bacteria, Chloroflexi, Ktedonobacteria, C0119	6.27			-
Bacteria, Actinobacteria, Actinobacteria, Acidimicrobiales, Iamiaceae	4.98			-
Bacteria, Actinobacteria, Actinobacteria, Frankiales, Acidothermaceae, Acidothermus	6.18			-
Bacteria, Chloroflexi, Ktedonobacteria, JG30_KF_AS9	6.99			-
Bacteria, Firmicutes, Bacilli, Bacillales, Planococcaceae, Sporosarcina	5.30			-

Bacteria, Saccharibacteria	7.11			-
Bacteria, Proteobacteria, Gammaproteobacteria, Legionellales, Legionellaceae, Legionella	4.64			-
Bacteria, Bacteroidetes, Cytophagia, Cytophagales, Cyclobacteriaceae	3.95			-
Bacteria, Proteobacteria, Gammaproteobacteria, Cellvibrionales, Porticoccaceae, C1_B045	4.26			-
Bacteria, Actinobacteria, Actinobacteria, Micrococcales, Micrococcaceae, Sinomonas	4.54			-
Bacteria, Proteobacteria, Alphaproteobacteria, Rickettsiales, RB446	4.16			-
Bacteria, Proteobacteria, Gammaproteobacteria, Aeromonadales	0.00			-
Bacteria, Proteobacteria, Deltaproteobacteria, Desulfuromonadales, Geobacteraceae	0.00			-
Bacteria, Firmicutes, Bacilli, Lactobacillales, Enterococcaceae, Enterococcus	6.03			-
Bacteria, Firmicutes, Bacilli, Bacillales, Paenibacillaceae, Cohnella	6.32			-
Bacteria, Proteobacteria, Gammaproteobacteria, Methylococcales, Methylococcaceae	4.04			-
Bacteria, Deinococcus_Thermus, Deinococci, Deinococcales, Trueperaceae	5.76			-
Bacteria, Firmicutes, Bacilli, Bacillales, Paenibacillaceae	6.84			-
Bacteria, Proteobacteria, Gammaproteobacteria, Xanthomonadales, Nevskiaceae	6.41			-
Bacteria, Firmicutes, Clostridia, Halanaerobiales, ODP1230B8_23	6.62			-
Bacteria, Firmicutes, Clostridia, Clostridiales, Christensenellaceae, Christensenellaceae_R_7_group	3.58			-
Bacteria, Proteobacteria, Alphaproteobacteria, Rhizobiales, Rhizobiales_Incertae_Sedis, Bauldia	5.21			-

Bacteria, Proteobacteria, Deltaproteobacteria, Bdellovibrionales, Bdellovibrionaceae, Bdellovibrio	5.98			-
Bacteria, Verrucomicrobia, Spartobacteria, Chthoniobacterales, Xiphinematobacteraceae	4.92			-
Bacteria, Proteobacteria, Gammaproteobacteria, Oceanospirillales, Alcanivoracaceae, Alcanivorax	5.92			-
Bacteria, Proteobacteria, Betaproteobacteria, SC_I_84	6.74			-
Bacteria, Actinobacteria, Actinobacteria, Micromonosporales, Micromonosporaceae	5.31	CK	4.96	0.05
Bacteria, Cyanobacteria, Cyanobacteria, SubsectionIII	4.73	LM	4.69	0.05
Bacteria, Actinobacteria, Actinobacteria, Propionibacteriales, Nocardioidaceae, Nocardioides	6.03			-
Bacteria, Actinobacteria, Actinobacteria, Streptosporangiales, Thermomonosporaceae	5.57			-
Bacteria, Proteobacteria, Deltaproteobacteria, Desulfurellales, Desulfurellaceae, H16	6.06			-
Bacteria, Acidobacteria, Acidobacteria, Acidobacteriales, Acidobacteriaceae__Subgroup_1, Granulicella	5.99			-
Bacteria, Acidobacteria, Acidobacteria, Solibacterales, Solibacteraceae__Subgroup_3_, PAUC26f	4.42			-
Bacteria, Proteobacteria, Gammaproteobacteria, Thiotrichales, Thiotrichaceae	0.00			-
Bacteria, Actinobacteria, Actinobacteria, Catenulisporales, Actinospicaceae	5.39	CK	4.98	0.05
Bacteria, Firmicutes, Limnochordia, Limnochordales	4.70			-
Bacteria, Actinobacteria, Actinobacteria, Pseudonocardiales, Pseudonocardiaceae, Pseudonocardia	5.03			-
Bacteria, Verrucomicrobia, Verrucomicrobiae, Verrucomicrobiales	4.35			-
Bacteria, Chlorobi, Chlorobia, Chlorobiales	5.73			-

Bacteria, Proteobacteria, Alphaproteobacteria, Rhodospirillales, KCM_B_15	4.51			-
Bacteria, Proteobacteria, Gammaproteobacteria, Aeromonadales, Aeromonadaceae	0.00			-
Bacteria, Actinobacteria, Actinobacteria, Pseudonocardiales	5.64			-
Bacteria, Proteobacteria, Betaproteobacteria, Burkholderiales, Oxalobacteraceae, Massilia	5.97			-
Bacteria, Bacteroidetes, Sphingobacteriia, Sphingobacteriales	5.45			-
Bacteria, Chloroflexi, Ktedonobacteria, Ktedonobacterales, FCPS473	5.40			-
Bacteria, Planctomycetes, Phycisphaerae, Phycisphaerales, Phycisphaeraceae	0.00			-
Bacteria, Proteobacteria, Alphaproteobacteria, Rhizobiales, Methylobacteriaceae, Microvirga	4.85			-
Bacteria, Proteobacteria, Alphaproteobacteria, Sphingomonadales, Sphingomonadaceae, Sphingobium	4.71			-
Bacteria, Firmicutes, Clostridia, Clostridiales, Family_XVII, Sulfobacillus	4.47			-
Bacteria, Proteobacteria, Gammaproteobacteria, Oceanospirillales, Alcanivoracaceae	5.92			-
Bacteria, Nitrospirae, Nitrospira, Nitrospira	6.12			-
Bacteria, Proteobacteria, Alphaproteobacteria, Rhizobiales, Xanthobacteraceae, Variibacter	5.48			-
Bacteria, Gemmatimonadetes, Gemmatimonadetes	4.66			-
Bacteria, Proteobacteria, Alphaproteobacteria, Rickettsiales, Rickettsiales_Incertae_Sedis	4.70			-
Bacteria, Bacteroidetes, Sphingobacteriia, Sphingobacteriales, CWT_CU03_E12	5.31			-
Bacteria, Chloroflexi, Ktedonobacteria	5.33			-

Bacteria, Proteobacteria, Alphaproteobacteria, Rhodospirillales, Acetobacteraceae, Acidiphilium	4.85			-
Bacteria, Proteobacteria, Alphaproteobacteria, Rhodospirillales, I_10	5.02			-
Bacteria, Chloroflexi, Caldilineae, Caldilineales	4.52			-
Bacteria, Proteobacteria, Deltaproteobacteria, Desulfurellales, Desulfurellaceae	6.06			-
Bacteria, Proteobacteria, Alphaproteobacteria, Rickettsiales, Holosporaceae	4.68			-
Bacteria, Actinobacteria, Actinobacteria, Pseudonocardiales, Pseudonocardiaceae, Lechevalieria	4.86	LM	4.66	0.05
Bacteria, Verrucomicrobia, Verrucomicrobiae, Verrucomicrobiales, Verrucomicrobiaceae, Haloferula	4.16			-
Bacteria, Bacteroidetes, Sphingobacteriia	7.51			-
Bacteria, Proteobacteria, Betaproteobacteria, Burkholderiales, Burkholderiaceae, Ralstonia	5.58			-
Bacteria, Actinobacteria, Actinobacteria, Corynebacteriales, Mycobacteriaceae, Mycobacterium	6.10			-
Bacteria, Chloroflexi, Anaerolineae	5.96			-
Bacteria, Bacteroidetes, Bacteroidia, Bacteroidales, Porphyromonadaceae	5.09			-
Bacteria, Actinobacteria	7.50			-
Bacteria, Proteobacteria, Alphaproteobacteria, Alphaproteobacteria_Incertae_Sedis	4.82			-
Bacteria, Actinobacteria, Actinobacteria, Acidimicrobiales, Acidimicrobiaceae, CL500_29_marine_group	4.45			-
Bacteria, Acidobacteria, Acidobacteria, Subgroup_10	5.25			-
Bacteria, Cyanobacteria, Cyanobacteria	5.90			-

Bacteria, Acidobacteria, Acidobacteria, Subgroup_7	5.88			-
Bacteria	6.00			-
**Taxonomy**	**Mean**	**Group**	**LDA**	***P* value**
Bacteria, Proteobacteria, Betaproteobacteria, Burkholderiales, Oxalobacteraceae, Pseudoduganella	4.21	OS	4.88	0.04
Bacteria, Actinobacteria, Actinobacteria, Streptosporangiales, Streptosporangiaceae, Streptosporangium	4.19			-
Bacteria, Firmicutes, Bacilli, Lactobacillales, Streptococcaceae	6.04			-
Bacteria, Proteobacteria, Deltaproteobacteria, Myxococcales, Archangiaceae	4.44	CK	4.56	0.05
Bacteria, Proteobacteria, Alphaproteobacteria, Rhizobiales, Brucellaceae, Ochrobactrum	4.43			-
Bacteria, Actinobacteria, Actinobacteria, Solirubrobacterales, YNPFFP1	5.55			-
Bacteria, Proteobacteria, Gammaproteobacteria, Xanthomonadales, Xanthomonadaceae	5.76			-
Bacteria, Proteobacteria, Alphaproteobacteria, Sphingomonadales, Erythrobacteraceae, Altererythrobacter	5.42			-
Bacteria, Proteobacteria, Gammaproteobacteria, Oceanospirillales, Hahellaceae, Hahella	3.68			-
Bacteria, Firmicutes, Clostridia, Clostridiales, Peptostreptococcaceae, Paeniclostridium	5.43	CK	5.11	0.05
Bacteria, Gemmatimonadetes, Gemmatimonadetes, Longimicrobiales	5.43			-
Bacteria, Proteobacteria, Alphaproteobacteria, Rhizobiales, Xanthobacteraceae, Pseudolabrys	7.07			-
Bacteria, Bacteroidetes, Sphingobacteriia, Sphingobacteriales, Chitinophagaceae, Lacibacter	4.01			-
Bacteria, Actinobacteria, Actinobacteria, Acidimicrobiales, Acidimicrobiaceae	4.82			-

Bacteria, Proteobacteria, Gammaproteobacteria, Xanthomonadales, Xanthomonadaceae, Dokdonella	5.67			-
Bacteria, Proteobacteria, Alphaproteobacteria, Sphingomonadales, Sphingomonadaceae, Zymomonas	3.71			-
Bacteria, Chlorobi, Chlorobia, Chlorobiales, OPB56	5.65			-
Bacteria, Proteobacteria, Alphaproteobacteria, Rhizobiales, Phyllobacteriaceae	6.57			-
Bacteria, Bacteroidetes, Sphingobacteriia, Sphingobacteriales, Sphingobacteriaceae, Nubsella	3.88			-
Bacteria, Actinobacteria, Actinobacteria, Pseudonocardiales, Pseudonocardiaceae, Kutzneria	4.55			-
Bacteria, Elusimicrobia, Elusimicrobia, Lineage_IIc	3.55			-
Bacteria, Elusimicrobia, Elusimicrobia, Lineage_IIb	4.73			-
Bacteria, Elusimicrobia, Elusimicrobia, Lineage_IIa	3.88			-
Bacteria, Proteobacteria, Alphaproteobacteria, Rhizobiales, Hyphomicrobiaceae, Rhodoplanes	5.46			-
Bacteria, Bacteroidetes	4.88			-
Bacteria, Actinobacteria, Actinobacteria, Corynebacteriales, Nocardiaceae, Rhodococcus	5.11			-
Bacteria, Proteobacteria, Deltaproteobacteria, Bdellovibrionales	5.79			-
Bacteria, Chloroflexi, Ktedonobacteria, Ktedonobacterales, Ktedonobacteraceae	6.55			-
Bacteria, Verrucomicrobia, OPB35_soil_group, Pedosphaera	4.44			-
Bacteria, Proteobacteria, Gammaproteobacteria, Pseudomonadales, Moraxellaceae	4.68	OS	4.71	0.04
Bacteria, Acidobacteria, Acidobacteria, Subgroup_10, ABS_19	5.25			-

Bacteria, Bacteroidetes, Flavobacteriia, Flavobacteriales, Flavobacteriaceae	6.44			-
Bacteria, Ignavibacteriae, Ignavibacteria, Ignavibacteriales	3.55			-
Bacteria, Actinobacteria, Actinobacteria, Propionibacteriales	6.48			-
Bacteria, Proteobacteria, Deltaproteobacteria, Bdellovibrionales, Bdellovibrionaceae, OM27_clade	4.33			-
Bacteria, Bacteroidetes, Cytophagia, Cytophagales, Cytophagaceae, Dyadobacter	4.53			-
Bacteria, Proteobacteria, Alphaproteobacteria, Sphingomonadales, Sphingomonadaceae, Sphingopyxis	5.54			-
Bacteria, Deinococcus_Thermus, Deinococci	5.29			-
Bacteria, Proteobacteria, Gammaproteobacteria, Legionellales, Legionellaceae	4.59			-
Bacteria, Proteobacteria, Gammaproteobacteria, Thiotrichales, EV818SWSAP88	3.93			-
Bacteria, Bacteroidetes, Sphingobacteriia, Sphingobacteriales, Sphingobacteriaceae, Sphingobacterium	5.20			-
Bacteria, Verrucomicrobia, Opitutae	5.74			-
Bacteria, Verrucomicrobia, OPB35_soil_group	6.19			-
Bacteria, Planctomycetes, Phycisphaerae	4.99			-
Bacteria, Bacteroidetes, Bacteroidia, Bacteroidales, Porphyromonadaceae, Petrimonas	5.09			-
Bacteria, Firmicutes, Bacilli, Lactobacillales, Carnobacteriaceae	4.41			-
Bacteria, Proteobacteria, Gammaproteobacteria, Enterobacteriales	5.15			-
Bacteria, Candidatus_Berkelbacteria	4.26			-

Bacteria, Bacteroidetes, Sphingobacteriia, Sphingobacteriales, Chitinophagaceae, Dinghuibacter	0.00			-
Bacteria, Proteobacteria, Alphaproteobacteria, Rhizobiales, Rhizobiales_Incertae_Sedis	6.82	CK	6.32	0.05
Bacteria, Proteobacteria, Gammaproteobacteria, Pseudomonadales, Moraxellaceae, Acinetobacter	4.40	OS	4.91	0.04
Bacteria, Proteobacteria, Deltaproteobacteria, Myxococcales, P3OB_42	5.33			-
Bacteria, Proteobacteria, Alphaproteobacteria, Rhizobiales, MNG7	4.42			-
Bacteria, Actinobacteria, Actinobacteria, Gaiellales, Gaiellaceae	6.00			-
Bacteria, Proteobacteria, Alphaproteobacteria, Rhizobiales, Hyphomicrobiaceae	6.60			-
Bacteria, Actinobacteria, Actinobacteria, Catenulisporales, Catenulisporaceae, Catenulispora	5.43			-
Bacteria, Firmicutes, Erysipelotrichia	5.30			-
Bacteria, Proteobacteria, Alphaproteobacteria, Rhizobiales	5.40			-
Bacteria, Deinococcus_Thermus, Deinococci, Deinococcales, Deinococcaceae, Deinococcus	3.71			-
Bacteria, Proteobacteria, Deltaproteobacteria, Myxococcales, Sandaracinaceae	6.11	CK	5.64	0.05
Bacteria, Firmicutes, Bacilli, Bacillales, Family_XII_o__Bacillales, Exiguobacterium	4.76			-
Bacteria, Proteobacteria, Alphaproteobacteria, Rhodospirillales, Acetobacteraceae	6.19			-
Bacteria, Bacteroidetes, Flavobacteriia, Flavobacteriales, Flavobacteriaceae, Arenibacter	4.52	OS	4.49	0.04
Bacteria, Armatimonadetes, Chthonomonadetes, Chthonomonadales, Chthonomonadaceae	4.28			-
Bacteria, Proteobacteria, Betaproteobacteria, Burkholderiales, Comamonadaceae, Aquincola	5.32			-

Bacteria, Firmicutes, Bacilli, Lactobacillales	6.34			-
Bacteria, Actinobacteria, Actinobacteria, Micrococcales, Bogoriellaceae	3.98			-
Bacteria, Proteobacteria, Betaproteobacteria, Burkholderiales, Oxalobacteraceae	5.75			-
Bacteria, Actinobacteria, Actinobacteria, Catenulisporales, Catenulisporaceae	5.43			-
Bacteria, Verrucomicrobia, Spartobacteria	6.39	CK	5.90	0.05
Bacteria, Chlorobi, Chlorobia	5.67			-
Bacteria, Proteobacteria, Gammaproteobacteria, Thiotrichales	3.93			-
Bacteria, Verrucomicrobia, Spartobacteria, Chthoniobacterales	3.58			-
Bacteria, Proteobacteria, Gammaproteobacteria, Xanthomonadales, Nevskiaceae, Alkanibacter	6.18			-
Bacteria, Proteobacteria, Alphaproteobacteria, Rhizobiales, Methylobacteriaceae, Methylobacterium	4.44			-
Bacteria, Proteobacteria, Alphaproteobacteria, Rhizobiales, Hyphomicrobiaceae, Devosia	4.46			-
Bacteria, Bacteroidetes, Sphingobacteriia, Sphingobacteriales, Chitinophagaceae, Segetibacter	4.46			-
Bacteria, Proteobacteria, Betaproteobacteria, Burkholderiales, Burkholderiaceae	3.71			-
Bacteria, Actinobacteria, Actinobacteria, Gaiellales	6.78			-
Bacteria, Proteobacteria, Gammaproteobacteria, Cellvibrionales, Cellvibrionaceae, Cellvibrio	5.29	OS	4.91	0.05
Bacteria, Bacteroidetes, Flavobacteriia, Flavobacteriales, Flavobacteriaceae, Muricauda	4.67			-
Bacteria, Acidobacteria, Acidobacteria, Solibacterales	6.83			-

Bacteria, Bacteroidetes, Sphingobacteriia, Sphingobacteriales, AKYH767	4.45			-
Bacteria, Acidobacteria, Acidobacteria	6.86			-
Bacteria, Firmicutes, Erysipelotrichia, Erysipelotrichales, Erysipelotrichaceae, Turicibacter	5.30			-
Bacteria, Proteobacteria, Gammaproteobacteria, Cellvibrionales, Porticoccaceae	4.26			-
Bacteria, Proteobacteria, Alphaproteobacteria, Rickettsiales, Rickettsiaceae, Candidatus_Trichorickettsia	0.00			-
Bacteria, Proteobacteria, Alphaproteobacteria, Rhodobacterales, Rhodobacteraceae, Rubellimicrobium	4.31			-
Bacteria, Proteobacteria, Alphaproteobacteria, Rhizobiales, Rhizobiales_Incertae_Sedis, Rhizomicrobium	6.78	CK	6.25	0.05
Bacteria, Deinococcus_Thermus	5.29			-
Bacteria, Acidobacteria, Acidobacteria, Solibacterales, Solibacteraceae__Subgroup_3_, AKIW659	4.51	CK	5.06	0.04
Bacteria, Chloroflexi, Thermomicrobia, Sphaerobacterales	6.48			-
Bacteria, Firmicutes, Erysipelotrichia, Erysipelotrichales, Erysipelotrichaceae	5.30			-
Bacteria, Proteobacteria, Alphaproteobacteria, Rhodobacterales, Rhodobacteraceae, Paracoccus	4.43			-
Bacteria, Chloroflexi, Ktedonobacteria, Ktedonobacterales, Thermosporotrichaceae, Thermosporothrix	4.72			-
Bacteria, Proteobacteria, Gammaproteobacteria, Xanthomonadales, Xanthomonadales_Incertae_Sedis, Steroidobacter	5.50			-
Bacteria, Proteobacteria, Alphaproteobacteria, Rickettsiales, Mitochondria	3.58			-
Bacteria, Proteobacteria, Alphaproteobacteria, Sphingomonadales, Sphingomonadaceae, Sphingomonas	7.34			-
Bacteria, Actinobacteria, Actinobacteria, Streptomycetales, Streptomycetaceae, Streptomyces	5.99			-

Bacteria, Proteobacteria, Gammaproteobacteria, Pseudomonadales, Pseudomonadaceae	5.57			-
Bacteria, Chloroflexi, Ktedonobacteria, Ktedonobacterales, 1959_1	4.47			-
Bacteria, Planctomycetes, Phycisphaerae, Tepidisphaerales	6.10	CK	5.67	0.05
Bacteria, Proteobacteria, Alphaproteobacteria, Alphaproteobacteria_Incertae_Sedis, Micavibrio	4.20			-
Bacteria, Proteobacteria, Gammaproteobacteria, Xanthomonadales, Xanthomonadaceae, Panacagrimonas	5.43	OS	5.08	0.05
Bacteria, Proteobacteria, Deltaproteobacteria, Myxococcales, mle1_27	0.00			-
Bacteria, Actinobacteria, Actinobacteria, Frankiales, Geodermatophilaceae	5.65	CK	5.23	0.05
Bacteria, Bacteroidetes, Sphingobacteriia, Sphingobacteriales, Sphingobacteriaceae, Mucilaginibacter	5.98			-
Bacteria, Bacteroidetes, Flavobacteriia, Flavobacteriales, Flavobacteriaceae, Confluentibacter	5.86	CK	5.36	0.05
Bacteria, Proteobacteria, Gammaproteobacteria, Xanthomonadales, Xanthomonadaceae, Lysobacter	5.87			-
Bacteria, Proteobacteria, Betaproteobacteria, Neisseriales, Neisseriaceae	3.68			-
Bacteria, Proteobacteria, Gammaproteobacteria, Methylococcales	4.01			-
Bacteria, Proteobacteria, Alphaproteobacteria, Sphingomonadales, Erythrobacteraceae	6.05			-
Bacteria, Peregrinibacteria, Candidatus_Peribacteria	4.98	OS	4.65	0.05
Bacteria, Proteobacteria, Gammaproteobacteria, Xanthomonadales, Xanthomonadaceae, Luteimonas	6.04			-
Bacteria, Proteobacteria, Alphaproteobacteria, Rhizobiales, Rhodobiaceae, Parvibaculum	5.71			-
Bacteria, Actinobacteria, Actinobacteria, Micrococcales, Demequinaceae, Lysinimicrobium	4.73	OS	4.53	0.04

Bacteria, Bacteroidetes, Sphingobacteriia, Sphingobacteriales, Chitinophagaceae, Crenotalea	6.43	CK	6.10	0.05
Bacteria, Proteobacteria, Gammaproteobacteria, Xanthomonadales, Xanthomonadaceae, Arenimonas	5.94			-
Bacteria, Firmicutes, Clostridia, Clostridiales, Clostridiaceae_1	4.54			-
Bacteria, Chloroflexi, P2_11E	4.67	CK	4.58	0.05
Bacteria, Planctomycetes, Phycisphaerae, Phycisphaerales	4.28			-
Bacteria, Firmicutes, Bacilli, Bacillales, Bacillaceae, Oceanobacillus	4.64			-
Bacteria, Proteobacteria, Betaproteobacteria, Burkholderiales, Comamonadaceae, Variovorax	4.52			-
Bacteria, Verrucomicrobia, Spartobacteria, Chthoniobacterales, Chthoniobacteraceae	4.91	OS	4.69	0.04
Bacteria, Bacteroidetes, Flavobacteriia, Flavobacteriales, Cryomorphaceae, Fluviicola	5.50	OS	5.20	0.05
Bacteria, Proteobacteria, Betaproteobacteria, Burkholderiales, Comamonadaceae, Delftia	4.39			-
Bacteria, Firmicutes, Bacilli, Lactobacillales, Streptococcaceae, Streptococcus	4.66			-
Bacteria, Chlamydiae, Chlamydiae, Chlamydiales	5.08			-
Bacteria, Proteobacteria, Gammaproteobacteria, Xanthomonadales, Xanthomonadaceae, Luteibacter	4.28			-
Bacteria, WS6	4.40	OS	4.99	0.04
Bacteria, Proteobacteria, Betaproteobacteria, Burkholderiales, Alcaligenaceae, Pusillimonas	5.29			-
Bacteria, Actinobacteria, Actinobacteria, Kineosporiales, Kineosporiaceae, Angustibacter	5.69	CK	5.29	0.05
Bacteria, Bacteroidetes, Flavobacteriia, Flavobacteriales, NS9_marine_group	4.91	OS	4.57	0.04

Bacteria, Actinobacteria, Actinobacteria, Streptomycetales	6.35			-
Bacteria, Bacteroidetes, Sphingobacteriia, Sphingobacteriales, Chitinophagaceae, Arachidicoccus	6.88			-
Bacteria, Bacteroidetes, Flavobacteriia, Flavobacteriales, Flavobacteriaceae, Vitellibacter	5.21	OS	4.93	0.05
Bacteria, Proteobacteria, Betaproteobacteria, Burkholderiales, Burkholderiaceae, Pandoraea	5.19			-
Bacteria, Proteobacteria, Betaproteobacteria, Burkholderiales, Comamonadaceae, Aquabacterium	0.00			-
Bacteria, Chloroflexi, Anaerolineae, Anaerolineales, Anaerolineaceae, Anaerolinea	4.21			-
Bacteria, Actinobacteria, Actinobacteria, Micromonosporales, Micromonosporaceae, Micromonospora	4.98			-
Bacteria, Firmicutes, Bacilli, Bacillales, Bacillaceae, Bacillus	6.10	CK	5.58	0.05
Bacteria, Proteobacteria, Betaproteobacteria, Rhodocyclales, Rhodocyclaceae, Uliginosibacterium	0.00			-
Bacteria, Bacteroidetes, Sphingobacteriia, Sphingobacteriales, Chitinophagaceae	7.09			-
Bacteria, Chloroflexi, Ktedonobacteria, Ktedonobacterales, G12_WMSP1	5.21	CK	4.83	0.05
Bacteria, Proteobacteria, Alphaproteobacteria, Caulobacterales, Hyphomonadaceae, Hirschia	4.85			-
Bacteria, Bacteroidetes, Cytophagia, Cytophagales, Cytophagaceae, Sporocytophaga	5.23			-
Bacteria, Proteobacteria, Gammaproteobacteria, Pseudomonadales	5.62			-
Bacteria, Actinobacteria, Actinobacteria, Streptosporangiales, Streptosporangiaceae	6.02	CK	5.66	0.05
Bacteria, Planctomycetes, Planctomycetacia, Planctomycetales, Planctomycetaceae, Planctomyces	3.59			-
Bacteria, Proteobacteria, Alphaproteobacteria, Rhodospirillales, DA111	6.49			-

Bacteria, Actinobacteria, Actinobacteria, Pseudonocardiales, Pseudonocardiaceae, Saccharothrix	4.54	OS	4.66	0.04
Bacteria, Proteobacteria, Alphaproteobacteria, Rhizobiales, Xanthobacteraceae	6.24			-
Bacteria, Bacteroidetes, Flavobacteriia, Flavobacteriales, Flavobacteriaceae, Galbibacter	4.38	OS	4.74	0.05
Bacteria, Proteobacteria, Gammaproteobacteria, Xanthomonadales, Xanthomonadales_Incertae_Sedis, Acidibacter	6.33			-
Bacteria, Firmicutes, Bacilli, Bacillales, Bacillaceae	6.12	CK	5.61	0.05
Bacteria, Actinobacteria, Actinobacteria, Frankiales, Sporichthyaceae	5.57			-
Bacteria, Proteobacteria, Alphaproteobacteria, Rickettsiales, Rickettsiaceae	0.00			-
Bacteria, Firmicutes, Clostridia, Clostridiales, Christensenellaceae	3.58			-
Bacteria, Proteobacteria, Alphaproteobacteria, Rhodospirillales, AKYH478	4.16			-
Bacteria, Acidobacteria, Acidobacteria, Vicinamibacter	4.24			-
Bacteria, Actinobacteria, Actinobacteria, Catenulisporales, Actinospicaceae, Actinospica	5.46			-
Bacteria, Ignavibacteriae, Ignavibacteria, Ignavibacteriales, BSV26	4.94	OS	4.62	0.05
Bacteria, Elusimicrobia, Elusimicrobia	5.46			-
Bacteria, Proteobacteria, Betaproteobacteria, Burkholderiales, Comamonadaceae	5.80			-
Bacteria, Proteobacteria, Gammaproteobacteria, Enterobacteriales, Enterobacteriaceae, _Serratia__fonticola_RB_25_group	4.03			-
Bacteria, Bacteroidetes, Cytophagia, Cytophagales, Cytophagaceae, Ohtaekwangia	4.97	OS	4.76	0.05
Bacteria, Proteobacteria, Gammaproteobacteria, Oceanospirillales, Oceanospirillaceae, Pseudohongiella	5.62	OS	5.23	0.05

Bacteria, Actinobacteria, Actinobacteria, Streptosporangiales	6.15	CK	5.82	0.05
Bacteria, Acidobacteria, Acidobacteria, Acidobacteriales, Acidobacteriaceae__Subgroup_1, Edaphobacter	5.70	CK	5.35	0.05
Bacteria, Acidobacteria, Acidobacteria, Holophagales, Holophagaceae	6.07	OS	5.64	0.05
Bacteria, Actinobacteria, Actinobacteria, Frankiales, Frankiaceae, Jatrophihabitans	6.09			-
Bacteria, Proteobacteria, Gammaproteobacteria, Pseudomonadales, Moraxellaceae, Psychrobacter	4.72			-
Bacteria, Firmicutes, Bacilli, Bacillales, Planococcaceae	5.83			-
Bacteria, Firmicutes, Bacilli, Bacillales, Planococcaceae, Solibacillus	5.31			-
Bacteria, Proteobacteria, Alphaproteobacteria, Rhodospirillales, Acetobacteraceae, Rhodovastum	5.90	CK	5.53	0.05
Bacteria, Proteobacteria, Betaproteobacteria, Hydrogenophilales, Hydrogenophilaceae, Thiobacillus	4.75			-
Bacteria, Firmicutes, Bacilli, Bacillales, Sporolactobacillaceae, Tuberibacillus	5.69	CK	5.38	0.05
Bacteria, Actinobacteria, Actinobacteria, Micrococcales, Intrasporangiaceae, Terrabacter	5.35			-
Bacteria, Chloroflexi, Elev_1554	3.88			-
Bacteria, Proteobacteria, Gammaproteobacteria, Oceanospirillales, Halomonadaceae	0.00			-
Bacteria, Actinobacteria, Actinobacteria, Micrococcales, Micrococcaceae	6.16			-
Bacteria, Chloroflexi, Thermomicrobia	6.31	CK	5.71	0.05
Bacteria, Proteobacteria, Deltaproteobacteria, Desulfurellales	6.06			-
Bacteria, Spirochaetae, Spirochaetes	4.84			-

Bacteria, Chloroflexi, Thermomicrobia, AKYG1722	5.32			-
Bacteria, Bacteroidetes, Sphingobacteriia, Sphingobacteriales, Chitinophagaceae, Flavitalea	5.20			-
Bacteria, Proteobacteria, Alphaproteobacteria, Rhodospirillales, Rhodospirillales_Incertae_Sedis	6.18			-
Bacteria, Firmicutes, Clostridia, Clostridiales, Lachnospiraceae, Lachnospiraceae_NK3A20_group	3.55			-
Bacteria, Planctomycetes, Planctomycetacia, Planctomycetales, Planctomycetaceae, Singulisphaera	5.65			-
Bacteria, Firmicutes, Bacilli, Bacillales, Thermoactinomycetaceae, Shimazuella	4.28			-
Bacteria, Actinobacteria, Actinobacteria, Micrococcales, Microbacteriaceae, Lysinimonas	6.20	CK	5.64	0.05
Bacteria, Firmicutes, Bacilli, C178B	6.18	CK	5.90	0.05
Bacteria, Firmicutes, Clostridia, Clostridiales, Peptostreptococcaceae	5.30	CK	4.89	0.05
Bacteria, Proteobacteria, Betaproteobacteria, Burkholderiales, Burkholderiaceae, Cupriavidus	4.57			-
Bacteria, Proteobacteria, Gammaproteobacteria, Xanthomonadales, Xanthomonadaceae, Dyella	6.74			-
Bacteria, Verrucomicrobia	6.63	CK	5.81	0.05
Bacteria, Actinobacteria, Actinobacteria, Solirubrobacterales, Gsoil_1167	3.96			-
Bacteria, Proteobacteria, Betaproteobacteria, Burkholderiales, Burkholderiaceae, Burkholderia_Paraburkholderia	6.36			-
Bacteria, Acidobacteria, Acidobacteria, Solibacterales, Solibacteraceae__Subgroup_3_, Candidatus_Solibacter	6.40			-
Bacteria, Proteobacteria, Deltaproteobacteria, Myxococcales, Phaselicystidaceae	4.16			-
Bacteria, Actinobacteria, Actinobacteria, Frankiales, Nakamurellaceae	5.31			-

Bacteria, Actinobacteria, Actinobacteria, Catenulisporales	5.75			-
Bacteria, Proteobacteria, Alphaproteobacteria, Rhizobiales, Roseiarcaceae, Roseiarcus	5.91	CK	5.46	0.05
Bacteria, Proteobacteria, Gammaproteobacteria, Cellvibrionales	0.00			-
Bacteria, Planctomycetes, OM190	3.68			-
Bacteria, Proteobacteria, Betaproteobacteria, TRA3_20	5.92			-
Bacteria, Proteobacteria, Betaproteobacteria, Burkholderiales, Comamonadaceae, Pelomonas	4.16			-
Bacteria, Planctomycetes, Planctomycetacia, Planctomycetales, Planctomycetaceae, Isosphaera	5.10	CK	4.87	0.05
Bacteria, Actinobacteria, Actinobacteria, Micrococcales, Bogoriellaceae, Georgenia	3.98			-
Bacteria, Bacteroidetes, Sphingobacteriia, Sphingobacteriales, env_OPS_17	5.23	OS	4.79	0.05
Bacteria, Proteobacteria, Gammaproteobacteria, Legionellales, Coxiellaceae	5.84			-
Bacteria, Bacteroidetes, Flavobacteriia, Flavobacteriales, Flavobacteriaceae, Gelidibacter	5.01			-
Bacteria, Proteobacteria, Gammaproteobacteria, Xanthomonadales, Xanthomonadaceae, Stenotrophomonas	5.56			-
Bacteria, Proteobacteria, Betaproteobacteria, Burkholderiales, Alcaligenaceae, Verticia	4.26			-
Bacteria, Proteobacteria, Alphaproteobacteria, Rhizobiales, Beijerinckiaceae, Methylovirgula	5.32	CK	4.96	0.05
Bacteria, Actinobacteria, Actinobacteria, Frankiales	6.15			-
Bacteria, Chloroflexi, Chloroflexia, Chloroflexales, Roseiflexaceae, Roseiflexus	5.47			-
Bacteria, Proteobacteria, Alphaproteobacteria, Rhizobiales, Rhizobiaceae	5.80			-

Bacteria, Planctomycetes, Planctomycetacia, Brocadiales	0.00			-
*Bacteria, Acidobacteria, Acidobacteria, Solibacterales, Solibacteraceae__Subgroup_3_*	5.42	OS	5.02	0.05
Bacteria, Firmicutes, Bacilli, Bacillales, Paenibacillaceae, Paenibacillus	6.41			-
Bacteria, Proteobacteria, Deltaproteobacteria, Myxococcales, Eel_36e1D6	3.71			-
Bacteria, Proteobacteria, Alphaproteobacteria, Rhizobiales, Bradyrhizobiaceae	5.40	CK	4.92	0.05
Bacteria, Acidobacteria, Acidobacteria, Blastocatellales, Blastocatellaceae__Subgroup_4, Tellurimicrobium	4.72	OS	4.56	0.05
Bacteria, Actinobacteria, Actinobacteria, Solirubrobacterales, Patulibacteraceae, Patulibacter	4.64			-
Bacteria, Actinobacteria, Actinobacteria, Propionibacteriales, Nocardioidaceae, Aeromicrobium	5.76	OS	5.35	0.05
Bacteria, Proteobacteria, Betaproteobacteria, Nitrosomonadales	6.74			-
Bacteria, Proteobacteria, Alphaproteobacteria, Rhizobiales, Methylobacteriaceae	4.95			-
Bacteria, Bacteroidetes, Cytophagia, Cytophagales, Cytophagaceae, Cytophaga	4.68			-
Bacteria, Actinobacteria, Actinobacteria, Frankiales, Sporichthyaceae, Sporichthya	4.67			-
Bacteria, Spirochaetae, Spirochaetes, Spirochaetales, Leptospiraceae	4.46			-
Bacteria, Bacteroidetes, Sphingobacteriia, Sphingobacteriales, Chitinophagaceae, Terrimonas	5.26			-
Bacteria, Proteobacteria, Alphaproteobacteria, Rhizobiales, Brucellaceae	4.43			-
Bacteria, Proteobacteria, Deltaproteobacteria, Bradymonadales	3.71			-
Bacteria, Proteobacteria, Gammaproteobacteria, Xanthomonadales	6.07			-

Bacteria, Proteobacteria, Alphaproteobacteria, Rhizobiales, Methylobacteriaceae, Meganema	6.83			-
Bacteria, Proteobacteria, Alphaproteobacteria, Rhizobiales, alphaI_cluster	4.82			-
Bacteria, Proteobacteria, Deltaproteobacteria, Bdellovibrionales, Bacteriovoracaceae, Peredibacter	4.81			-
Bacteria, Armatimonadetes, Fimbriimonadia, Fimbriimonadales	5.13			-
Bacteria, Acidobacteria, Acidobacteria, Acidobacteriales, Acidobacteriaceae__Subgroup_1, Acidobacterium	6.19	CK	5.62	0.05
Bacteria, Acidobacteria, Acidobacteria, Solibacterales, Solibacteraceae__Subgroup_3_, Bryobacter	6.63	CK	6.13	0.05
Bacteria, Actinobacteria, Actinobacteria, Corynebacteriales, Nocardiaceae, Nocardia	5.02			-
Bacteria, Spirochaetae, Spirochaetes, Spirochaetales, Spirochaetaceae	4.61			-
Bacteria, Chlamydiae, Chlamydiae, Chlamydiales, Parachlamydiaceae, Neochlamydia	4.24			-
Bacteria, Firmicutes, Bacilli, Bacillales, Alicyclobacillaceae, Tumebacillus	5.11			-
Bacteria, Parcubacteria, Candidatus_Nomurabacteria	3.71			-
Bacteria, Bacteroidetes, Sphingobacteriia, Sphingobacteriales, Sphingobacteriaceae	5.33			-
Bacteria, Bacteroidetes, Sphingobacteriia, Sphingobacteriales, Chitinophagaceae, Chitinophaga	5.75			-
Bacteria, Proteobacteria, Alphaproteobacteria, Rhizobiales, KF_JG30_B3	5.26			-
Bacteria, Proteobacteria, Betaproteobacteria, Methylophilales	5.45			-
Bacteria, Chloroflexi, Ktedonobacteria, B12_WMSP1	5.60	CK	5.21	0.05
Bacteria, Proteobacteria, Betaproteobacteria, Methylophilales, Methylophilaceae	4.48			-

Bacteria, Proteobacteria, Alphaproteobacteria, Rhodobacterales, Rhodobacteraceae	4.04			-
Bacteria, Bacteroidetes, Cytophagia, Cytophagales, Cytophagaceae, Adhaeribacter	0.00			-
Bacteria, Armatimonadetes, Chthonomonadetes	4.28			-
Bacteria, Actinobacteria, Actinobacteria, Corynebacteriales, Nocardiaceae, Smaragdicoccus	4.81	OS	4.67	0.04
Bacteria, Proteobacteria, Alphaproteobacteria, Rhizobiales, Beijerinckiaceae	5.33			-
Bacteria, Proteobacteria, Alphaproteobacteria	5.32			-
Bacteria, Proteobacteria, Alphaproteobacteria, Rhodospirillales, Rhodospirillaceae	6.01			-
Bacteria, Proteobacteria, Alphaproteobacteria, Rhizobiales, Hyphomicrobiaceae, Hyphomicrobium	5.80	CK	5.36	0.05
Bacteria, Proteobacteria, Gammaproteobacteria, Aeromonadales, Aeromonadaceae, Aeromonas	0.00			-
Bacteria, Firmicutes, Clostridia, Clostridiales, Family_XIII, Mogibacterium	4.20			-
Bacteria, Proteobacteria, ARKICE_90	3.71			-
Bacteria, Proteobacteria, Alphaproteobacteria, Caulobacterales, Hyphomonadaceae, Woodsholea	4.70			-
Bacteria, Proteobacteria, Alphaproteobacteria, Caulobacterales, Caulobacteraceae, Caulobacter	4.95			-
Bacteria, Proteobacteria, Gammaproteobacteria, Xanthomonadales, Xanthomonadaceae, Tahibacter	4.31			-
Bacteria, Ignavibacteriae	4.96	OS	4.64	0.05
Bacteria, Bacteroidetes, Flavobacteriia, Flavobacteriales, Flavobacteriaceae, Chryseobacterium	5.26			-
Bacteria, Proteobacteria, Deltaproteobacteria, Bdellovibrionales, Bacteriovoracaceae	4.66	OS	4.68	0.04

Bacteria, Proteobacteria, Betaproteobacteria	4.71			-
Bacteria, Actinobacteria, Actinobacteria, Corynebacteriales	6.17			-
Bacteria, Proteobacteria, Alphaproteobacteria, Caulobacterales, Caulobacteraceae	6.06			-
Bacteria, Proteobacteria, Alphaproteobacteria, Rhizobiales, Xanthobacteraceae, Labrys	3.55			-
*Bacteria, TM6__Dependentiae_*	5.03			-
Bacteria, Proteobacteria, Betaproteobacteria, Burkholderiales, Burkholderiaceae, Limnobacter	5.18			-
Bacteria, Deinococcus_Thermus, Deinococci, Deinococcales, Deinococcaceae	3.71			-
Bacteria, Bacteroidetes, Cytophagia, Cytophagales, Cytophagaceae, Leadbetterella	0.00			-
Bacteria, Proteobacteria, Deltaproteobacteria, Oligoflexales, 0319_6G20	5.85			-
Bacteria, Parcubacteria, Candidatus_Azambacteria	4.53	OS	4.61	0.04
Bacteria, Latescibacteria	5.22			-
Bacteria, Armatimonadetes	4.21			-
Bacteria, Firmicutes	7.32			-
Bacteria, Firmicutes, Clostridia, Halanaerobiales	6.62	CK	6.16	0.05
Bacteria, Proteobacteria, Deltaproteobacteria, Myxococcales, Haliangiaceae	5.57			-
Bacteria, GAL15	5.85			-
Bacteria, Planctomycetes, Phycisphaerae, Tepidisphaerales, Tepidisphaeraceae	6.10	CK	5.65	0.05

Bacteria, Chloroflexi, S085	4.99			-
Bacteria, Acidobacteria, Acidobacteria, Blastocatellales, Blastocatellaceae__Subgroup_4, Stenotrophobacter	4.41			-
Bacteria, Bacteroidetes, Sphingobacteriia, Sphingobacteriales, Chitinophagaceae, Filimonas	5.28			-
Bacteria, Planctomycetes, Phycisphaerae, Phycisphaerales, Phycisphaeraceae, SM1A02	4.28			-
Bacteria, Chloroflexi, Thermomicrobia, Sphaerobacterales, Sphaerobacteraceae, Nitrolancea	5.86	CK	5.30	0.05
Bacteria, Firmicutes, Clostridia, Clostridiales, Lachnospiraceae	0.00			-
Bacteria, Bacteroidetes, Bacteroidia, Bacteroidales, Porphyromonadaceae, Parabacteroides	3.55			-
Bacteria, Proteobacteria, Alphaproteobacteria, Rhizobiales, Xanthobacteraceae, Pseudoxanthobacter	3.71			-
Bacteria, Firmicutes, Limnochordia	4.70			-
Bacteria, Fibrobacteres, Fibrobacteria, Fibrobacterales	5.11	OS	4.85	0.05
Bacteria, Proteobacteria, Alphaproteobacteria, Rickettsiales, SM2D12	5.90	OS	5.46	0.05
Bacteria, Proteobacteria, Betaproteobacteria, Neisseriales, Neisseriaceae, Vitreoscilla	3.71			-
Bacteria, Proteobacteria, Alphaproteobacteria, Rhodospirillales, MNC12	3.55			-
Bacteria, Proteobacteria, Gammaproteobacteria, Oceanospirillales, Hahellaceae	6.16			-
Bacteria, Gemmatimonadetes, Gemmatimonadetes, Longimicrobiales, Longimicrobiaceae	5.43			-
Bacteria, Chlamydiae	5.08			-
Bacteria, Actinobacteria, Actinobacteria, Micrococcales, Micrococcaceae, Arthrobacter	5.46			-

Bacteria, Actinobacteria, Actinobacteria, Frankiales, Frankiaceae	6.09			-
Bacteria, Proteobacteria, Gammaproteobacteria, Legionellales, Coxiellaceae, Coxiella	4.50			-
Bacteria, Firmicutes, Erysipelotrichia, Erysipelotrichales	5.30			-
Bacteria, Proteobacteria, Alphaproteobacteria, Rhizobiales, Phyllobacteriaceae, Mesorhizobium	6.48			-
Bacteria, Proteobacteria, Deltaproteobacteria, Myxococcales, Polyangiaceae	4.16			-
Bacteria, Proteobacteria, Alphaproteobacteria, Rhizobiales, Methylocystaceae	4.36			-
Bacteria, Actinobacteria, Actinobacteria, Frankiales, Geodermatophilaceae, Blastococcus	5.66	CK	5.15	0.05
Bacteria, Proteobacteria, Deltaproteobacteria, Myxococcales, Myxococcaceae	3.93			-
Bacteria, Proteobacteria, Deltaproteobacteria, Myxococcales, Phaselicystidaceae, Phaselicystis	4.16			-
Bacteria, Bacteroidetes, Cytophagia, Cytophagales, Cytophagaceae, Chryseolinea	5.44	OS	5.02	0.05
Bacteria, Acidobacteria, Acidobacteria, Acidobacteriales, Acidobacteriaceae__Subgroup_1, Candidatus_Koribacter	5.37	CK	4.91	0.05
Bacteria, Proteobacteria, Gammaproteobacteria, Pseudomonadales, Moraxellaceae, Perlucidibaca	4.28			-
Bacteria, Proteobacteria, Alphaproteobacteria, Rhizobiales, Beijerinckiaceae, Methylorosula	3.68			-
Bacteria, Firmicutes, Bacilli, Bacillales, Planococcaceae, Lysinibacillus	5.42			-
Bacteria, Proteobacteria, Alphaproteobacteria, Rhizobiales, Rhizobiales_Incertae_Sedis, Agaricicola	5.33			-
Bacteria, Firmicutes, Bacilli, Lactobacillales, Carnobacteriaceae, Carnobacterium	4.41			-
Bacteria, Proteobacteria, Betaproteobacteria, Burkholderiales, Alcaligenaceae, Eoetvoesia	5.26	CK	4.99	0.05

Bacteria, Actinobacteria, Actinobacteria, Streptosporangiales, Streptosporangiaceae, Nonomuraea	4.61			-
Bacteria, Bacteroidetes, Sphingobacteriia, Sphingobacteriales, Sphingobacteriaceae, Pedobacter	5.82			-
Bacteria, Gemmatimonadetes	7.35			-
Bacteria, Parcubacteria	6.41			-
Bacteria, Acidobacteria, Acidobacteria, Blastocatellales	6.16			-
Bacteria, Proteobacteria, Deltaproteobacteria	3.71			-
Bacteria, Proteobacteria, Alphaproteobacteria, Rhizobiales, Bradyrhizobiaceae, Bradyrhizobium	6.60	CK	6.05	0.05
Bacteria, Actinobacteria, Actinobacteria, Solirubrobacterales, FFCH11085	4.52			-
Bacteria, Actinobacteria, Actinobacteria, Frankiales, Acidothermaceae	6.18			-
Bacteria, Proteobacteria, Deltaproteobacteria, Bdellovibrionales, Bdellovibrionaceae	5.77			-
Bacteria, FCPU426	5.00			-
Bacteria, Firmicutes, Bacilli, Bacillales, Paenibacillaceae, Aneurinibacillus	4.19			-
Bacteria, Actinobacteria, Actinobacteria, Acidimicrobiales, Iamiaceae, Iamia	5.17			-
Bacteria, Actinobacteria, Actinobacteria, Acidimicrobiales, OM1_clade	3.59			-
Bacteria, Proteobacteria, Betaproteobacteria, Nitrosomonadales, Nitrosomonadaceae	6.68			-
Bacteria, Proteobacteria, Gammaproteobacteria, Legionellales, Coxiellaceae, Rickettsiella	4.89			-
Bacteria, Proteobacteria, Alphaproteobacteria, Rhizobiales, Rhizobiaceae, Kaistia	4.43			-

Bacteria, Bacteroidetes, Cytophagia, Cytophagales	6.66	OS	6.23	0.05
Bacteria, Chloroflexi, Thermomicrobia, JG30_KF_CM45	6.04			-
Bacteria, Bacteroidetes, Sphingobacteriia, Sphingobacteriales, Chitinophagaceae, Parasegetibacter	4.03			-
Bacteria, Proteobacteria, Alphaproteobacteria, Caulobacterales	6.40			-
Bacteria, Bacteroidetes, Cytophagia, Cytophagales, Cytophagaceae	6.59	OS	6.23	0.05
Bacteria, Proteobacteria, Alphaproteobacteria, Rhodospirillales, MND8	4.16			-
Bacteria, Proteobacteria, Deltaproteobacteria, NB1_j	4.44	CK	4.59	0.04
Bacteria, Proteobacteria, Betaproteobacteria, Burkholderiales, Alcaligenaceae, Paralcaligenes	5.26			-
Bacteria, Proteobacteria, Alphaproteobacteria, Rhizobiales, Rhizobiales_Incertae_Sedis, Nordella	5.16			-
Bacteria, Firmicutes, Bacilli, Bacillales, Thermoactinomycetaceae	4.28			-
Bacteria, Peregrinibacteria	4.11			-
Bacteria, Proteobacteria, Alphaproteobacteria, Rhizobiales, Bradyrhizobiaceae, Bosea	4.23			-
Bacteria, Proteobacteria, Betaproteobacteria, Burkholderiales, Comamonadaceae, Rhizobacter	4.79			-
Bacteria, Proteobacteria, Gammaproteobacteria, Cellvibrionales, Cellvibrionaceae, Simiduia	4.75	OS	4.65	0.04
Bacteria, Proteobacteria, Deltaproteobacteria, Myxococcales, Blfdi19	4.82			-
Bacteria, Actinobacteria, Actinobacteria, Acidimicrobiales	6.19			-
Bacteria, Armatimonadetes, Chthonomonadetes, Chthonomonadales	4.28			-

Bacteria, Proteobacteria, Betaproteobacteria, Methylophilales, Methylophilaceae, Methylotenera	4.88			-
Bacteria, Proteobacteria, Deltaproteobacteria, Oligoflexales	5.96			-
Bacteria, Proteobacteria, Betaproteobacteria, Rhodocyclales	4.72			-
Bacteria, Fibrobacteres, Fibrobacteria	5.11	OS	4.80	0.05
Bacteria, Proteobacteria, Gammaproteobacteria, Xanthomonadales, Xanthomonadaceae, Rhodanobacter	7.84	CK	7.44	0.05
Bacteria, Proteobacteria, Betaproteobacteria, Nitrosomonadales, Nitrosomonadaceae, Nitrosospira	5.78			-
Bacteria, Proteobacteria, Deltaproteobacteria, Desulfuromonadales, Geobacteraceae, Geobacter	3.59			-
Bacteria, Bacteroidetes, Sphingobacteriia, Sphingobacteriales, Chitinophagaceae, Flavihumibacter	4.34			-
Bacteria, Chlamydiae, Chlamydiae, Chlamydiales, cvE6	4.94			-
Bacteria, Firmicutes, Limnochordia, Limnochordales, Limnochordaceae	4.98	OS	4.63	0.05
Bacteria, Proteobacteria, Gammaproteobacteria	6.51			-
Bacteria, Actinobacteria, Actinobacteria	5.78			-
Bacteria, Chloroflexi, SBR2076	5.52	CK	4.84	0.05
Bacteria, Parcubacteria, Candidatus_Jorgensenbacteria	4.26			-
Bacteria, Proteobacteria, Alphaproteobacteria, Rhodospirillales, Rhodospirillaceae, Dongia	5.20			-
Bacteria, Acidobacteria	7.53			-
Bacteria, Chloroflexi, Chloroflexia, Chloroflexales, FFCH7168	3.96			-

Bacteria, Actinobacteria, Actinobacteria, Acidimicrobiales, Acidimicrobiales_Incertae_Sedis	3.59			-
Bacteria, Proteobacteria, Betaproteobacteria, Hydrogenophilales, Hydrogenophilaceae	4.75			-
Bacteria, Actinobacteria, Actinobacteria, Micromonosporales, Micromonosporaceae, Dactylosporangium	4.54			-
Bacteria, Cyanobacteria, Cyanobacteria, SubsectionIII, FamilyI_o__SubsectionIII	4.63			-
Bacteria, Actinobacteria, Actinobacteria, Micrococcales, Intrasporangiaceae	6.28			-
Bacteria, Proteobacteria, Gammaproteobacteria, Oceanospirillales, Oceanospirillaceae	5.62	OS	5.25	0.05
Bacteria, Bacteroidetes, Flavobacteriia, Flavobacteriales, Flavobacteriaceae, Aequorivita	5.85	OS	5.49	0.05
Bacteria, Actinobacteria, Actinobacteria, Solirubrobacterales	5.44	OS	5.11	0.05
Bacteria, Actinobacteria, Actinobacteria, Micrococcales, Microbacteriaceae, Leifsonia	4.70			-
Bacteria, Proteobacteria, Gammaproteobacteria, Xanthomonadales, Xanthomonadaceae, Thermomonas	5.02	OS	4.69	0.05
Bacteria, Ignavibacteriae, Ignavibacteria	4.96	OS	4.64	0.05
Bacteria, Proteobacteria, Deltaproteobacteria, Oligoflexales, Oligoflexaceae	5.30			-
Bacteria, Proteobacteria, Deltaproteobacteria, Myxococcales, 27F_1492R	4.36			-
Bacteria, Fibrobacteres, Fibrobacteria, Fibrobacterales, Fibrobacteraceae	4.77	OS	4.74	0.04
Bacteria, Actinobacteria, Actinobacteria, Propionibacteriales, Nocardioidaceae, Marmoricola	6.07			-
Bacteria, Actinobacteria, Actinobacteria, Micrococcales, Microbacteriaceae, Leucobacter	3.58			-
Bacteria, Chloroflexi, Ktedonobacteria, B10_SB3A	5.20	CK	4.80	0.05

Bacteria, Chloroflexi, Caldilineae, Caldilineales, Caldilineaceae	4.52			-
Bacteria, Bacteroidetes, Sphingobacteriia, Sphingobacteriales, Sphingobacteriaceae, Pseudopedobacter	0.00			-
Bacteria, Saccharibacteria, Candidatus_Saccharimonas	3.59			-
Bacteria, Actinobacteria, Actinobacteria, Pseudonocardiales, Pseudonocardiaceae, Amycolatopsis	5.29			-
Bacteria, Acidobacteria, Acidobacteria, Acidobacteriales, Acidobacteriaceae__Subgroup_1	7.12	CK	6.54	0.05
Bacteria, Firmicutes, Clostridia, Clostridiales, Family_XIII	4.20			-
Bacteria, Proteobacteria, Alphaproteobacteria, Rhizobiales, Hyphomicrobiaceae, Rhodomicrobium	3.71			-
Bacteria, Actinobacteria, Actinobacteria, Kineosporiales, Kineosporiaceae	5.64			-
Bacteria, Actinobacteria, Actinobacteria, Pseudonocardiales, Pseudonocardiaceae	5.55			-
Bacteria, Actinobacteria, Actinobacteria, Micromonosporales, Micromonosporaceae, Hamadaea	4.78			-
Bacteria, Acidobacteria, Acidobacteria, Blastocatellales, Blastocatellaceae__Subgroup_4, RB41	5.10			-
Bacteria, Acidobacteria, Acidobacteria, Acidobacteriales	7.22	CK	6.59	0.05
Bacteria, Nitrospirae, Nitrospira	6.12			-
Bacteria, Armatimonadetes, Chthonomonadetes, Chthonomonadales, Chthonomonadaceae, Chthonomonas	4.28			-
Bacteria, Actinobacteria, Actinobacteria, Micrococcales, Microbacteriaceae, Humibacter	5.67	CK	5.29	0.05
Bacteria, Acidobacteria, Acidobacteria, Solibacterales, Solibacteraceae__Subgroup_3_, Paludibaculum	4.53			-
Bacteria, Fibrobacteres	5.11	OS	4.82	0.05

Bacteria, Verrucomicrobia, Verrucomicrobiae, Verrucomicrobiales, Verrucomicrobiaceae, Verrucomicrobium	0.00			-
Bacteria, Proteobacteria, Gammaproteobacteria, Xanthomonadales, Xanthomonadaceae, Frateuria	6.30	CK	5.94	0.05
Bacteria, Proteobacteria, Deltaproteobacteria, Bradymonadales, Bradymonadaceae, Bradymonas	3.71			-
Bacteria, Spirochaetae, Spirochaetes, Spirochaetales, Leptospiraceae, Turneriella	4.46			-
Bacteria, Cyanobacteria	5.91			-
Bacteria, Acidobacteria, Acidobacteria, Holophagales, Holophagaceae, Geothrix	4.92			-
Bacteria, Proteobacteria, Betaproteobacteria, Burkholderiales, Comamonadaceae, Ottowia	5.69	CK	5.27	0.05
Bacteria, Proteobacteria, Gammaproteobacteria, Thiotrichales, Thiotrichaceae, Beggiatoa	0.00			-
Bacteria, Bacteroidetes, Flavobacteriia, Flavobacteriales, Flavobacteriaceae, Moheibacter	4.82			-
Bacteria, Proteobacteria, Alphaproteobacteria, Sphingomonadales, Sphingomonadaceae	7.35			-
Bacteria, Proteobacteria, Alphaproteobacteria, Rhizobiales, Bradyrhizobiaceae, Rhodopseudomonas	5.33			-
Bacteria, Proteobacteria, Alphaproteobacteria, Rhodospirillales, Rhodospirillaceae, Ferrovibrio	4.72	OS	4.93	0.04
Bacteria, Chloroflexi, Ktedonobacteria, Ktedonobacterales	5.32	CK	4.91	0.05
Bacteria, Bacteroidetes, Flavobacteriia	6.54			-
Bacteria, Proteobacteria, Alphaproteobacteria, Rhodospirillales, Rhodospirillaceae, Inquilinus	4.72			-
Bacteria, Proteobacteria, Betaproteobacteria, Nitrosomonadales, Nitrosomonadaceae, Nitrosomonas	4.26			-
Bacteria, Actinobacteria, Actinobacteria, Micromonosporales	5.88			-

Bacteria, Firmicutes, Bacilli, Bacillales, Alicyclobacillaceae, Alicyclobacillus	4.96			-
Bacteria, Bacteroidetes, Flavobacteriia, Flavobacteriales, Flavobacteriaceae, Flavobacterium	5.83			-
Bacteria, Proteobacteria, Alphaproteobacteria, Sphingomonadales, DSSF69	4.69			-
Bacteria, Proteobacteria, Gammaproteobacteria, Enterobacteriales, Enterobacteriaceae	5.15			-
Bacteria, Firmicutes, Clostridia, Clostridiales, Family_XVII	4.59			-
Bacteria, Actinobacteria, Actinobacteria, Solirubrobacterales, TM146	5.25			-
Bacteria, Bacteroidetes, Bacteroidia, Bacteroidales	5.09			-
Bacteria, Elusimicrobia	5.46			-
Bacteria, Proteobacteria, Betaproteobacteria, B1_7BS	3.93			-
Bacteria, Proteobacteria, Alphaproteobacteria, Rhizobiales, Hyphomicrobiaceae, Pedomicrobium	4.46			-
Bacteria, Actinobacteria, Actinobacteria, Solirubrobacterales, Solirubrobacteraceae	3.93			-
Bacteria, Proteobacteria, Gammaproteobacteria, Enterobacteriales, Enterobacteriaceae, Serratia	3.59			-
Bacteria, Chloroflexi, JG30_KF_CM66	5.76			-
Bacteria, Actinobacteria, Actinobacteria, Streptosporangiales, Streptosporangiaceae, Sphaerisporangium	4.98			-
Bacteria, BRC1	4.62			-
Bacteria, Planctomycetes	6.46	CK	5.94	0.05
Bacteria, Proteobacteria, Alphaproteobacteria, Rhodospirillales, Acetobacteraceae, Acidicaldus	5.02	CK	4.66	0.05

Bacteria, Chloroflexi, Thermomicrobia, Sphaerobacterales, Sphaerobacteraceae	5.86	CK	5.39	0.05
Bacteria, Actinobacteria, Actinobacteria, Micrococcales, Demequinaceae	4.73	OS	4.53	0.04
Bacteria, Actinobacteria, Actinobacteria, Frankiales, Nakamurellaceae, Nakamurella	5.31			-
Bacteria, Proteobacteria, JTB23	4.91	OS	4.67	0.04
Bacteria, Proteobacteria, Deltaproteobacteria, Myxococcales, Haliangiaceae, Haliangium	5.22	OS	4.83	0.05
Bacteria, Proteobacteria, Gammaproteobacteria, HTA4	4.67			-
Bacteria, Proteobacteria, Alphaproteobacteria, Rhizobiales, Roseiarcaceae	5.91	CK	5.43	0.05
Bacteria, Proteobacteria, Gammaproteobacteria, Legionellales	5.87			-
Bacteria, Chloroflexi, KD4_96	6.32			-
Bacteria, Firmicutes, Bacilli, Lactobacillales, Enterococcaceae	6.03			-
Bacteria, Actinobacteria, Actinobacteria, Streptosporangiales, Thermomonosporaceae, Actinomadura	5.57	CK	5.21	0.05
Bacteria, Actinobacteria, Actinobacteria, Micrococcales	6.89			-
Bacteria, Proteobacteria, Alphaproteobacteria, Caulobacterales, Caulobacteraceae, Phenylobacterium	5.89			-
Bacteria, Armatimonadetes, Fimbriimonadia	5.13			-
Bacteria, Actinobacteria, Actinobacteria, Acidimicrobiales, Acidimicrobiaceae, Ilumatobacter	4.49			-
Bacteria, Chloroflexi, Ktedonobacteria, Ktedonobacterales, HSB_OF53_F07	6.40	CK	5.93	0.05
Bacteria, Proteobacteria, Gammaproteobacteria, Oceanospirillales, Halomonadaceae, Halomonas	0.00			-

Bacteria, Proteobacteria, Alphaproteobacteria, Rhodospirillales, JG37_AG_20	5.77			-
Bacteria, Microgenomates	5.46			-
Bacteria, Cyanobacteria, Cyanobacteria, SubsectionIII, FamilyI_o__SubsectionIII, Phormidium	0.00			-
Bacteria, Planctomycetes, Planctomycetacia, Brocadiales, Brocadiaceae, Candidatus_Kuenenia	6.16			-
Bacteria, Parcubacteria, Candidatus_Magasanikbacteria	4.75			-
Bacteria, Verrucomicrobia, Spartobacteria, Chthoniobacterales, Chthoniobacteraceae, Chthoniobacter	4.98			-
Bacteria, Actinobacteria, Actinobacteria, Streptomycetales, Streptomycetaceae	6.09			-
Bacteria, Firmicutes, Bacilli	3.96			-
Bacteria, Proteobacteria, Gammaproteobacteria, Xanthomonadales, Solimonadaceae, Solimonas	3.88			-
Bacteria, Proteobacteria, Deltaproteobacteria, Desulfuromonadales	3.59			-
Bacteria, Proteobacteria, Betaproteobacteria, Burkholderiales, Alcaligenaceae, Candidimonas	6.40	CK	6.09	0.05
Bacteria, Proteobacteria, Gammaproteobacteria, Cellvibrionales, Cellvibrionaceae	5.40	OS	5.05	0.05
Bacteria, Bacteroidetes, Sphingobacteriia, Sphingobacteriales, Chitinophagaceae, Taibaiella	6.76			-
Bacteria, Actinobacteria, Actinobacteria, Micrococcales, Dermacoccaceae, Flexivirga	5.63	CK	5.29	0.05
Bacteria, Actinobacteria, Actinobacteria, Micrococcales, Cellulomonadaceae, Actinotalea	4.32			-
Bacteria, Proteobacteria, Alphaproteobacteria, Rhizobiales, Rhizobiaceae, Rhizobium	5.78			-
Bacteria, Proteobacteria, Alphaproteobacteria, Sphingomonadales	5.39			-

Bacteria, Chloroflexi, Chloroflexia	5.48			-
Bacteria, Verrucomicrobia, Opitutae, Opitutales, Opitutaceae	5.74			-
Bacteria, Gemmatimonadetes, Gemmatimonadetes, Gemmatimonadales, Gemmatimonadaceae, Gemmatimonas	0.00			-
Bacteria, Proteobacteria, Alphaproteobacteria, Sphingomonadales, Sphingomonadaceae, Novosphingobium	5.20			-
Bacteria, Acidobacteria, Acidobacteria, Blastocatellales, Blastocatellaceae__Subgroup_4	6.09			-
Bacteria, Spirochaetae	4.84			-
Bacteria, Proteobacteria, Deltaproteobacteria, Myxococcales	5.90			-
Bacteria, Actinobacteria, Actinobacteria, Micrococcales, Dermacoccaceae	5.63	CK	5.32	0.05
Bacteria, Proteobacteria, Gammaproteobacteria, Xanthomonadales, Xanthomonadales_Incertae_Sedis	6.37			-
Bacteria, Planctomycetes, BD7_11	4.18			-
Bacteria, Proteobacteria, Betaproteobacteria, Burkholderiales, Comamonadaceae, Ramlibacter	6.00			-
Bacteria, Chloroflexi, Ktedonobacteria, Ktedonobacterales, Thermosporotrichaceae	5.56			-
Bacteria, Proteobacteria, Betaproteobacteria, Burkholderiales, Comamonadaceae, Comamonas	3.59			-
Bacteria, Actinobacteria, Actinobacteria, Gaiellales, Gaiellaceae, Gaiella	6.00			-
Bacteria, Proteobacteria, Gammaproteobacteria, Oceanospirillales	5.96			-
Bacteria, Firmicutes, Clostridia, Clostridiales	3.98			-
Bacteria, Verrucomicrobia, Verrucomicrobiae, Verrucomicrobiales, Verrucomicrobiaceae	4.28			-

Bacteria, Proteobacteria	6.04			-
Bacteria, Proteobacteria, Betaproteobacteria, Burkholderiales, Alcaligenaceae, Castellaniella	7.06			-
Bacteria, Elusimicrobia, Elusimicrobia, Lineage_IV	5.36			-
Bacteria, Proteobacteria, Alphaproteobacteria, Rhizobiales, Phyllobacteriaceae, Aquamicrobium	5.42			-
Bacteria, Proteobacteria, Deltaproteobacteria, Bradymonadales, Bradymonadaceae	3.71			-
Bacteria, Actinobacteria, Actinobacteria, Micromonosporales, Micromonosporaceae, Luedemannella	4.99			-
Bacteria, Proteobacteria, Deltaproteobacteria, Myxococcales, Sandaracinaceae, Sandaracinus	4.40			-
Bacteria, Planctomycetes, Planctomycetacia, Planctomycetales, Planctomycetaceae	6.00	CK	5.52	0.05
Bacteria, Gemmatimonadetes, Gemmatimonadetes, Gemmatimonadales, Gemmatimonadaceae	7.01			-
Bacteria, Firmicutes, Bacilli, Lactobacillales, Streptococcaceae, Lactococcus	6.02			-
Bacteria, Chlorobi, Chlorobia, Chlorobiales, SJA_28	4.38	OS	4.81	0.04
Bacteria, Actinobacteria, Actinobacteria, Solirubrobacterales, Solirubrobacteraceae, Solirubrobacter	3.93			-
Bacteria, Verrucomicrobia, Spartobacteria, Chthoniobacterales, DA101_soil_group	6.35	CK	5.90	0.05
Bacteria, Actinobacteria, Actinobacteria, Propionibacteriales, Nocardioidaceae, Kribbella	5.20			-
Bacteria, Bacteroidetes, Sphingobacteriia, Sphingobacteriales, PHOS_HE51	3.86			-
Bacteria, Bacteroidetes, Sphingobacteriia, Sphingobacteriales, Chitinophagaceae, Sediminibacterium	5.23			-
Bacteria, Actinobacteria, Actinobacteria, Micrococcales, Cellulomonadaceae	4.32			-

Bacteria, Spirochaetae, Spirochaetes, Spirochaetales	6.18			-
Bacteria, Gemmatimonadetes, Gemmatimonadetes, Gemmatimonadales	7.35			-
Bacteria, Chloroflexi, Ktedonobacteria, Ktedonobacterales, 1921_3	5.66			-
Bacteria, Bacteroidetes, Cytophagia	6.66	OS	6.25	0.05
Bacteria, Bacteroidetes, Flavobacteriia, Flavobacteriales, Cryomorphaceae, Wandonia	5.52			-
Bacteria, Proteobacteria, Alphaproteobacteria, Caulobacterales, Caulobacteraceae, Asticcacaulis	5.27			-
Bacteria, Proteobacteria, Gammaproteobacteria, Xanthomonadales, Nevskiaceae, Nevskia	4.16			-
Bacteria, Proteobacteria, Gammaproteobacteria, Xanthomonadales, Solimonadaceae	5.02			-
Bacteria, Deinococcus_Thermus, Deinococci, Deinococcales, Trueperaceae, Truepera	5.29			-
Bacteria, Proteobacteria, Alphaproteobacteria, Caulobacterales, Caulobacteraceae, Brevundimonas	5.50			-
Bacteria, Chloroflexi, Chloroflexia, Chloroflexales, Roseiflexaceae	5.47			-
Bacteria, Bacteroidetes, Flavobacteriia, Flavobacteriales, Cryomorphaceae	0.00			-
Bacteria, Proteobacteria, Deltaproteobacteria, Myxococcales, Polyangiaceae, Sorangium	4.80			-
Bacteria, Acidobacteria, Acidobacteria, Holophagales, Holophagaceae, Holophaga	6.10			-
Bacteria, Chloroflexi, Ktedonobacteria, Ktedonobacterales, BacC_u_018	5.19	CK	4.88	0.05
Bacteria, Bacteroidetes, Flavobacteriia, Flavobacteriales	6.54			-
Bacteria, Proteobacteria, Gammaproteobacteria, Methylococcales, Methylococcaceae, Methylocaldum	4.84			-

Bacteria, Proteobacteria, Alphaproteobacteria, Rhodospirillales	6.09			-
Bacteria, Verrucomicrobia, Verrucomicrobiae, Verrucomicrobiales, Verrucomicrobiaceae, Luteolibacter	3.68			-
Bacteria, Proteobacteria, Alphaproteobacteria, Rhodospirillales, Rhodospirillales_Incertae_Sedis, Reyranella	6.18			-
Bacteria, FBP	5.80	CK	5.33	0.05
Bacteria, Armatimonadetes, Fimbriimonadia, Fimbriimonadales, Fimbriimonadaceae	5.13			-
Bacteria, Chlamydiae, Chlamydiae, Chlamydiales, Parachlamydiaceae	4.24			-
Bacteria, Verrucomicrobia, Opitutae, Opitutales, Opitutaceae, Opitutus	5.74			-
Bacteria, Planctomycetes, Planctomycetacia, Planctomycetales	6.20	CK	5.64	0.05
Bacteria, Proteobacteria, Gammaproteobacteria, Xanthomonadales, Xanthomonadaceae, Mizugakiibacter	7.65			-
Bacteria, Planctomycetes, Phycisphaerae, Phycisphaerales, Phycisphaeraceae, Phycisphaera	0.00			-
Bacteria, Actinobacteria, Actinobacteria, Propionibacteriales, Nocardioidaceae	6.48			-
Bacteria, Proteobacteria, Betaproteobacteria, Burkholderiales, Oxalobacteraceae, Duganella	0.00			-
Bacteria, Verrucomicrobia, Opitutae, Opitutales	5.74			-
Bacteria, Parcubacteria, Candidatus_Campbellbacteria	5.78	OS	5.51	0.04
Bacteria, Actinobacteria, Actinobacteria, Micrococcales, Promicromonosporaceae	4.29			-
Bacteria, Bacteroidetes, Sphingobacteriia, Sphingobacteriales, Chitinophagaceae, Parafilimonas	4.01			-
Bacteria, Proteobacteria, Alphaproteobacteria, Rhodobacterales	4.80			-

Bacteria, Proteobacteria, Deltaproteobacteria, Myxococcales, Archangiaceae, Anaeromyxobacter	5.55			-
Bacteria, Proteobacteria, Gammaproteobacteria, Legionellales, Coxiellaceae, Aquicella	5.77			-
Bacteria, Firmicutes, Bacilli, Bacillales, Sporolactobacillaceae	6.41	CK	6.14	0.05
Bacteria, Proteobacteria, Gammaproteobacteria, Xanthomonadales, Xanthomonadaceae, Rudaea	4.26			-
Bacteria, Firmicutes, Bacilli, Bacillales	7.07			-
Bacteria, Proteobacteria, Deltaproteobacteria, Myxococcales, BIrii41	4.94	OS	4.67	0.04
Bacteria, Proteobacteria, Betaproteobacteria, Burkholderiales, Alcaligenaceae, Achromobacter	5.06			-
Bacteria, Firmicutes, Bacilli, Bacillales, Paenibacillaceae, Brevibacillus	6.35			-
Bacteria, Actinobacteria, Actinobacteria, Micrococcales, Promicromonosporaceae, Cellulosimicrobium	4.29			-
Bacteria, Chloroflexi, Ktedonobacteria, Thermogemmatisporales	5.40			-
Bacteria, Proteobacteria, Betaproteobacteria, Rhodocyclales, Rhodocyclaceae	4.72			-
Bacteria, Proteobacteria, Deltaproteobacteria, Myxococcales, Nannocystaceae	4.25	OS	4.71	0.04
Bacteria, Acidobacteria, Acidobacteria, Blastocatellales, Blastocatellaceae__Subgroup_4, 11_24	5.00			-
Bacteria, Actinobacteria, Actinobacteria, Corynebacteriales, Mycobacteriaceae	6.10			-
Bacteria, Chlorobi	4.28			-
Bacteria, Bacteroidetes, Sphingobacteriia, Sphingobacteriales, Chitinophagaceae, Niastella	4.72			-
Bacteria, Actinobacteria, Actinobacteria, Solirubrobacterales, Patulibacteraceae	4.64			-

Bacteria, Chloroflexi, Chloroflexia, Chloroflexales	5.48			-
Bacteria, Bacteroidetes, Flavobacteriia, Flavobacteriales, Flavobacteriaceae, Pricia	5.89	OS	5.51	0.05
Bacteria, Proteobacteria, Alphaproteobacteria, Rickettsiales	5.91			-
Bacteria, Chloroflexi, Ktedonobacteria, Thermogemmatisporales, 1921_2	5.40			-
Bacteria, Actinobacteria, Actinobacteria, Streptosporangiales, Streptosporangiaceae, Microbispora	5.96	CK	5.63	0.05
Bacteria, Cyanobacteria, Cyanobacteria, Vampirovibrionales	4.74			-
Bacteria, Verrucomicrobia, Verrucomicrobiae	4.28			-
Bacteria, Actinobacteria, Actinobacteria, Corynebacteriales, Nocardiaceae	5.46			-
Bacteria, Planctomycetes, Planctomycetacia	6.20	CK	5.63	0.05
Bacteria, Bacteroidetes, Sphingobacteriia, Sphingobacteriales, Saprospiraceae	5.21	OS	4.86	0.05
Bacteria, Proteobacteria, Alphaproteobacteria, Rhodobacterales, Rhodobacteraceae, Rhodobacter	4.28			-
Bacteria, Chloroflexi, TK10	6.13			-
Bacteria, Proteobacteria, Betaproteobacteria, Burkholderiales, Comamonadaceae, Xenophilus	4.85			-
Bacteria, Bacteroidetes, Sphingobacteriia, Sphingobacteriales, NS11_12_marine_group	5.67			-
Bacteria, Proteobacteria, Gammaproteobacteria, Pseudomonadales, Pseudomonadaceae, Pseudomonas	5.57			-
Bacteria, Bacteroidetes, Bacteroidia	5.09			-
Bacteria, Actinobacteria, Actinobacteria, Micrococcales, Microbacteriaceae	6.08	CK	5.68	0.05

Bacteria, Gemmatimonadetes, Gemmatimonadetes, Gemmatimonadales, Gemmatimonadaceae, Gemmatirosa	5.69			-
Bacteria, Actinobacteria, Actinobacteria, Acidimicrobiales, Acidimicrobiales_Incertae_Sedis, Candidatus_Microthrix	3.59			-
*Bacteria, Proteobacteria, Deltaproteobacteria, SAR324_clade_Marine_group_B_*	4.40			-
Bacteria, Chloroflexi, Gitt_GS_136	5.14			-
Bacteria, Acidobacteria, Acidobacteria, Blastocatellales, Blastocatellaceae__Subgroup_4, DS_100	3.55			-
Bacteria, Proteobacteria, Gammaproteobacteria, Enterobacteriales, Enterobacteriaceae, Kluyvera	5.10			-
Bacteria, Proteobacteria, Betaproteobacteria, Neisseriales	3.96			-
Bacteria, Proteobacteria, Alphaproteobacteria, Rickettsiales, Rickettsiales_Incertae_Sedis, Candidatus_Odyssella	4.26			-
Bacteria, Deinococcus_Thermus, Deinococci, Deinococcales	5.29			-
Bacteria, Proteobacteria, Betaproteobacteria, Burkholderiales, Alcaligenaceae	5.95			-
Bacteria, Bacteroidetes, Sphingobacteriia, Sphingobacteriales, KD3_93	4.67			-
Bacteria, Chloroflexi, Ktedonobacteria, Ktedonobacterales, JG30a_KF_32	6.19	CK	5.72	0.05
Bacteria, Chlamydiae, Chlamydiae	5.12			-
Bacteria, Proteobacteria, Gammaproteobacteria, Xanthomonadales, Xanthomonadaceae, Pseudoxanthomonas	4.78	OS	4.63	0.05
Bacteria, Proteobacteria, Gammaproteobacteria, Xanthomonadales, Solimonadaceae, Polycyclovorans	4.99			-
Bacteria, Spirochaetae, Spirochaetes, Spirochaetales, Spirochaetaceae, Spirochaeta_2	4.61			-
Bacteria, Nitrospirae	6.12			-

Bacteria, Proteobacteria, Betaproteobacteria, Methylophilales, Methylophilaceae, Methylobacillus	5.06	OS	4.69	0.05
Bacteria, Firmicutes, Bacilli, Bacillales, Alicyclobacillaceae	5.34	CK	5.02	0.05
Bacteria, Verrucomicrobia, Spartobacteria, Chthoniobacterales, Xiphinematobacteraceae, Candidatus_Xiphinematobacter	4.92			-
Bacteria, Bacteroidetes, Sphingobacteriia, Sphingobacteriales, Sphingobacteriaceae, Parapedobacter	4.48	OS	4.80	0.04
Bacteria, Fibrobacteres, Fibrobacteria, Fibrobacterales, Fibrobacteraceae, possible_genus_04	4.84	OS	4.54	0.05
Bacteria, Cyanobacteria, Cyanobacteria, SubsectionIII, FamilyI_o__SubsectionIII, Microcoleus	0.00			-
Bacteria, Bacteroidetes, Cytophagia, Cytophagales, Cytophagaceae, Emticicia	4.16			-
Bacteria, Firmicutes, Clostridia, Clostridiales, Clostridiaceae_1, Clostridium_sensu_stricto_1	5.62	CK	5.13	0.05
Bacteria, Firmicutes, Bacilli, Bacillales, Sporolactobacillaceae, Sporolactobacillus	6.32	CK	6.05	0.04
Bacteria, Firmicutes, Clostridia, Clostridiales, Clostridiaceae_1, Clostridium_sensu_stricto_9	4.06			-
Bacteria, Proteobacteria, Alphaproteobacteria, Sphingomonadales, Sphingomonadaceae, Rhizorhapis	4.67			-
Bacteria, Bacteroidetes, Flavobacteriia, Flavobacteriales, Flavobacteriaceae, Empedobacter	5.08			-
Bacteria, Proteobacteria, Alphaproteobacteria, Rhodospirillales, Acetobacteraceae, Acidisoma	4.26			-
Bacteria, Proteobacteria, Alphaproteobacteria, Rhizobiales, Phyllobacteriaceae, Aminobacter	5.60			-
Bacteria, Bacteroidetes, Bacteroidia, Bacteroidales, Porphyromonadaceae, Dysgonomonas	0.00			-
Bacteria, Chloroflexi, Anaerolineae, Anaerolineales	5.91			-
Bacteria, Bacteroidetes, Sphingobacteriia, Sphingobacteriales, Chitinophagaceae, Flavisolibacter	5.96			-

Bacteria, Chlamydiae, Chlamydiae, Chlamydiales, Simkaniaceae	4.73	OS	4.56	0.05
Bacteria, Chloroflexi, Anaerolineae, Anaerolineales, Anaerolineaceae	5.91			-
Bacteria, Actinobacteria, Actinobacteria, Solirubrobacterales, Elev_16S_1332	4.53			-
Bacteria, Bacteroidetes, Sphingobacteriia, Sphingobacteriales, Chitinophagaceae, Ferruginibacter	4.59			-
Bacteria, Chloroflexi	5.33			-
Bacteria, Acidobacteria, Acidobacteria, Holophagales	6.40			-
Bacteria, Actinobacteria, Actinobacteria, Solirubrobacterales, 0319_6M6	4.90			-
Bacteria, Proteobacteria, Betaproteobacteria, Hydrogenophilales	4.75			-
Bacteria, Proteobacteria, Alphaproteobacteria, Rhizobiales, Rhodobiaceae	5.50			-
Bacteria, Firmicutes, Bacilli, Bacillales, Planococcaceae, Sporosarcina	5.30			-
Bacteria, Saccharibacteria	4.38			-
Bacteria, Firmicutes, Bacilli, Bacillales, Family_XII_o__Bacillales	4.76			-
Bacteria, Proteobacteria, Alphaproteobacteria, Caulobacterales, Hyphomonadaceae	4.16			-
Bacteria, Chloroflexi, JG37_AG_4	6.86			-
Bacteria, Firmicutes, Clostridia	4.93			-
Bacteria, Chloroflexi, SHA_26	4.63			-
Bacteria, Chloroflexi, Caldilineae	4.52			-

Bacteria, Bacteroidetes, Sphingobacteriia, Sphingobacteriales, Chitinophagaceae, Heliimonas	4.16			-
Bacteria, Planctomycetes, Planctomycetacia, Brocadiales, Brocadiaceae	0.00			-
Bacteria, Omnitrophica	3.58			-
Bacteria, Proteobacteria, Betaproteobacteria, Burkholderiales, Oxalobacteraceae, Paucimonas	4.47			-
Bacteria, Chloroflexi, Ktedonobacteria, C0119	6.27	CK	5.69	0.05
Bacteria, Actinobacteria, Actinobacteria, Acidimicrobiales, Iamiaceae	5.17			-
Bacteria, Actinobacteria, Actinobacteria, Frankiales, Acidothermaceae, Acidothermus	6.18			-
Bacteria, Chloroflexi, Ktedonobacteria, JG30_KF_AS9	6.99	CK	6.57	0.05
Bacteria, Firmicutes, Clostridia, Clostridiales, Christensenellaceae, Christensenellaceae_R_7_group	3.58			-
Bacteria, Proteobacteria, Alphaproteobacteria, Rhizobiales, Rhizobiales_Incertae_Sedis, Bauldia	7.11			-
Bacteria, Proteobacteria, Gammaproteobacteria, Legionellales, Legionellaceae, Legionella	4.59			-
Bacteria, Bacteroidetes, Cytophagia, Cytophagales, Cyclobacteriaceae	4.24			-
Bacteria, Proteobacteria, Gammaproteobacteria, Cellvibrionales, Porticoccaceae, C1_B045	4.26			-
Bacteria, Actinobacteria, Actinobacteria, Micrococcales, Micrococcaceae, Sinomonas	4.34			-
Bacteria, Proteobacteria, Alphaproteobacteria, Rickettsiales, RB446	4.16			-
Bacteria, Proteobacteria, Gammaproteobacteria, Aeromonadales	0.00			-
Bacteria, Proteobacteria, Deltaproteobacteria, Desulfuromonadales, Geobacteraceae	3.59			-

Bacteria, Firmicutes, Bacilli, Lactobacillales, Enterococcaceae, Enterococcus	6.03			-
Bacteria, Firmicutes, Bacilli, Bacillales, Paenibacillaceae, Cohnella	6.32			-
Bacteria, Proteobacteria, Gammaproteobacteria, Methylococcales, Methylococcaceae	4.01			-
Bacteria, Deinococcus_Thermus, Deinococci, Deinococcales, Trueperaceae	5.29			-
Bacteria, Firmicutes, Bacilli, Bacillales, Paenibacillaceae	6.84			-
Bacteria, Proteobacteria, Gammaproteobacteria, Xanthomonadales, Nevskiaceae	6.18			-
Bacteria, Firmicutes, Clostridia, Halanaerobiales, ODP1230B8_23	6.62	CK	6.15	0.05
Bacteria, Actinobacteria, Actinobacteria, Pseudonocardiales, Pseudonocardiaceae, Pseudonocardia	4.91			-
Bacteria, Verrucomicrobia, Verrucomicrobiae, Verrucomicrobiales	5.13	CK	4.77	0.05
Bacteria, Proteobacteria, Deltaproteobacteria, Bdellovibrionales, Bdellovibrionaceae, Bdellovibrio	5.76			-
Bacteria, Verrucomicrobia, Spartobacteria, Chthoniobacterales, Xiphinematobacteraceae	4.92			-
Bacteria, Proteobacteria, Gammaproteobacteria, Oceanospirillales, Alcanivoracaceae, Alcanivorax	5.92	CK	5.67	0.05
Bacteria, Proteobacteria, Betaproteobacteria, SC_I_84	6.74	CK	6.31	0.05
Bacteria, Actinobacteria, Actinobacteria, Micromonosporales, Micromonosporaceae	5.31			-
Bacteria, Cyanobacteria, Cyanobacteria, SubsectionIII	4.63			-
Bacteria, Actinobacteria, Actinobacteria, Propionibacteriales, Nocardioidaceae, Nocardioides	6.05			-
Bacteria, Actinobacteria, Actinobacteria, Streptosporangiales, Thermomonosporaceae	5.57	CK	5.23	0.05

Bacteria, Proteobacteria, Deltaproteobacteria, Desulfurellales, Desulfurellaceae, H16	6.06			-
Bacteria, Acidobacteria, Acidobacteria, Acidobacteriales, Acidobacteriaceae__Subgroup_1, Granulicella	5.99			-
Bacteria, Acidobacteria, Acidobacteria, Solibacterales, Solibacteraceae__Subgroup_3_, PAUC26f	4.89	OS	4.86	0.04
Bacteria, Proteobacteria, Gammaproteobacteria, Thiotrichales, Thiotrichaceae	0.00			-
Bacteria, Actinobacteria, Actinobacteria, Catenulisporales, Actinospicaceae	5.46			-
Bacteria, Firmicutes, Limnochordia, Limnochordales	4.70			-
Bacteria, Gemmatimonadetes, Gemmatimonadetes	4.93			-
Bacteria, Proteobacteria, Alphaproteobacteria, Rickettsiales, Rickettsiales_Incertae_Sedis	4.28			-
Bacteria, Chlorobi, Chlorobia, Chlorobiales	5.67			-
Bacteria, Proteobacteria, Alphaproteobacteria, Rhodospirillales, KCM_B_15	4.48			-
Bacteria, Proteobacteria, Gammaproteobacteria, Aeromonadales, Aeromonadaceae	0.00			-
Bacteria, Actinobacteria, Actinobacteria, Pseudonocardiales	5.55			-
Bacteria, Proteobacteria, Betaproteobacteria, Burkholderiales, Oxalobacteraceae, Massilia	5.90			-
Bacteria, Bacteroidetes, Sphingobacteriia, Sphingobacteriales	5.89	OS	5.44	0.05
Bacteria, Chloroflexi, Ktedonobacteria, Ktedonobacterales, FCPS473	5.31			-
Bacteria, Planctomycetes, Phycisphaerae, Phycisphaerales, Phycisphaeraceae	3.55			-
Bacteria, Proteobacteria, Alphaproteobacteria, Rhizobiales, Methylobacteriaceae, Microvirga	4.85			-

Bacteria, Proteobacteria, Alphaproteobacteria, Sphingomonadales, Sphingomonadaceae, Sphingobium	4.71			-
Bacteria, Firmicutes, Clostridia, Clostridiales, Family_XVII, Sulfobacillus	4.59			-
Bacteria, Proteobacteria, Gammaproteobacteria, Oceanospirillales, Alcanivoracaceae	5.92	CK	5.62	0.05
Bacteria, Nitrospirae, Nitrospira, Nitrospira	6.12			-
Bacteria, Proteobacteria, Alphaproteobacteria, Rhizobiales, Xanthobacteraceae, Variibacter	5.48			-
Bacteria	6.00			-
Bacteria, Actinobacteria, Actinobacteria, Corynebacteriales, Mycobacteriaceae, Mycobacterium	4.26			-
Bacteria, Bacteroidetes, Sphingobacteriia, Sphingobacteriales, CWT_CU03_E12	5.46			-
Bacteria, Chloroflexi, Ktedonobacteria	5.33	CK	4.96	0.05
Bacteria, Proteobacteria, Alphaproteobacteria, Rhodospirillales, Acetobacteraceae, Acidiphilium	4.85			-
Bacteria, Proteobacteria, Alphaproteobacteria, Rhodospirillales, I_10	5.02			-
Bacteria, Chloroflexi, Caldilineae, Caldilineales	4.52			-
Bacteria, Proteobacteria, Deltaproteobacteria, Desulfurellales, Desulfurellaceae	6.06			-
Bacteria, Proteobacteria, Alphaproteobacteria, Rickettsiales, Holosporaceae	4.52			-
Bacteria, Actinobacteria, Actinobacteria, Pseudonocardiales, Pseudonocardiaceae, Lechevalieria	4.68	OS	4.52	0.05
Bacteria, Verrucomicrobia, Verrucomicrobiae, Verrucomicrobiales, Verrucomicrobiaceae, Haloferula	4.16			-
Bacteria, Bacteroidetes, Sphingobacteriia	7.51			-

Bacteria, Proteobacteria, Betaproteobacteria, Burkholderiales, Burkholderiaceae, Ralstonia	5.58			-
Bacteria, Actinobacteria, Actinobacteria, Corynebacteriales, Mycobacteriaceae, Mycobacterium	6.10			-
Bacteria, Chloroflexi, Anaerolineae	5.91			-
Bacteria, Bacteroidetes, Bacteroidia, Bacteroidales, Porphyromonadaceae	5.09			-
Bacteria, Actinobacteria	7.50			-
Bacteria, Proteobacteria, Alphaproteobacteria, Alphaproteobacteria_Incertae_Sedis	4.76			-
Bacteria, Actinobacteria, Actinobacteria, Acidimicrobiales, Acidimicrobiaceae, CL500_29_marine_group	4.55			-
Bacteria, Acidobacteria, Acidobacteria, Subgroup_10	5.25			-
Bacteria, Cyanobacteria, Cyanobacteria	5.90			-
Bacteria, Acidobacteria, Acidobacteria, Subgroup_7	5.88			-
Bacteria	6.00			-
